# Phase I clinical trial repurposing all-trans retinoic acid as a stromal targeting agent for pancreatic cancer

**DOI:** 10.1038/s41467-020-18636-w

**Published:** 2020-09-24

**Authors:** Hemant M. Kocher, Bristi Basu, Fieke E. M. Froeling, Debashis Sarker, Sarah Slater, Dominic Carlin, Nandita M. deSouza, Katja N. De Paepe, Michelle R. Goulart, Christine Hughes, Ahmet Imrali, Rhiannon Roberts, Maria Pawula, Richard Houghton, Cheryl Lawrence, Yathushan Yogeswaran, Kelly Mousa, Carike Coetzee, Peter Sasieni, Aaron Prendergast, David J. Propper

**Affiliations:** 1 Centre for Tumour Biology, Barts Cancer Institute—A CRUK Centre of Excellence, Queen Mary University London, London, EC1M 6BQ UK; 2 Centre for Experimental Cancer Medicine, Barts Cancer Institute—A CRUK Centre of Excellence, Queen Mary University of London, London, EC1M 6BQ UK; 3 Barts and the London HPB Centre, The Royal London Hospital, Barts Health NHS Trust, Whitechapel, London, E1 1FR UK; 4 Barts Pancreas Tissue Bank, Barts Cancer Institute—A CRUK Centre of Excellence, Queen Mary University London, London, EC1M 6BQ UK; 5 Department of Oncology, University of Cambridge and Cambridge University Hospitals NHS Foundation Trust—Addenbrooke’s Hospital, Cambridge, CB2 0QQ UK; 6 Department of Surgery and Cancer, Imperial College London—Hammersmith Hospital, London, W12 0HS UK; 7 School of Cancer and Pharmaceutical Sciences, King’s College London, Guy’s Hospital Campus, London, SE1 9RT UK; 8 Division of Radiotherapy and Imaging, The Institute of Cancer Research, London, SW7 3RP UK; 9 PK/Bioanalytics Core Facility, Cancer Research UK Cambridge Institute, University of Cambridge, Li Ka Shing Centre, Robinson Way, Cambridge, CB2 0RE UK; 10 Cancer Prevention Trials Unit, Wolfson Institute of Preventive Medicine, Queen Mary University of London, London, EC1M 6BQ UK; 11 Centre for Cancer and Inflammation, Barts Cancer Institute—A CRUK Centre of Excellence, Queen Mary University London, London, EC1M 6BQ UK; 12 Present Address: Cold Spring Harbor Laboratory, 1 Bungtown Road, Cold Spring Harbor, NY 11724 USA; 13 Present Address: School of Cancer & Pharmaceutical Sciences, and King’s Clinical Trials Unit, King’s College London, London, SE1 9RT UK

**Keywords:** Pancreatic cancer, Phase I trials, Pancreatic cancer

## Abstract

Pre-clinical models have shown that targeting pancreatic stellate cells with all-trans-retinoic-acid (ATRA) reprograms pancreatic stroma to suppress pancreatic ductal adenocarcinoma (PDAC) growth. Here, in a phase Ib, dose escalation and expansion, trial for patients with advanced, unresectable PDAC (n = 27), ATRA is re-purposed as a stromal-targeting agent in combination with gemcitabine-nab-paclitaxel chemotherapy using a two-step adaptive continual re-assessment method trial design. The maximum tolerated dose (MTD) and recommended phase 2 dose (RP2D, primary outcome) is the FDA/EMEA approved dose of gemcitabine-nab-paclitaxel along-with ATRA (45 mg/m^2^ orally, days 1–15/cycle). Dose limiting toxicity (DLT) is grade 4 thrombocytopenia (n = 2). Secondary outcomes show no detriment to ATRA pharmacokinetics.. Median overall survival for RP2D treated evaluable population, is 11.7 months (95%CI 8.6–15.7 m, n = 15, locally advanced (2) and metastatic (13)). Exploratory pharmacodynamics studies including changes in diffusion-weighted (DW)-MRI measured apparent diffusion coefficient after one cycle, and, modulation of cycle-specific serum pentraxin 3 levels over various cycles indicate stromal modulation. Baseline stromal-specific retinoid transport protein (FABP5, CRABP2) expression may be predicitve of response. Re-purposing ATRA as a stromal-targeting agent with gemcitabine-nab-paclitaxel is safe and tolerable. This combination will be evaluated in a phase II randomized controlled trial for locally advanced PDAC. Clinical trial numbers: EudraCT: 2015-002662-23; NCT03307148. Trial acronym: STARPAC.

## Abstract (web summary)

All-trans retinoic acid - ATRA- is known to remodulate the stroma of pancreatic cancer in mice. Here, the authors carried out a Phase Ib trial in pancreatic patients and show that ATRA in combination with chemotherapy is a safe potential treatment for patients with advanced pancreatic cancer, and demonstrate a stromal modulatory effect.

## Introduction

Advanced pancreatic ductal adenocarcinoma (PDAC) has a dismal prognosis with modestly effective treatment options. Desmoplastic stroma and hypo-vascularity, distinctive features of PDAC, impede successful delivery of chemotherapeutic drugs. Pancreatic stellate cells (PSC), critical components and instigators of desmoplasia, mediate cancer cell pro-survival and pro-invasive capabilities through multiple signaling cascades^1^. This tumor–stroma cross-talk is unlikely to be blocked effectively by merely targeting a single pathway. Targeting the multi-faceted tumor-promoting cancer–stromal cell interactions (i.e., normalizing the desmoplastic stroma) may, however, enhance the effectiveness of conventional chemotherapy.

Patients with PDAC display fat-soluble vitamin deficiencies due to impaired biliary and pancreatic secretions. Although vitamin K deficiency is manifested and treated clinically, the lack of vitamin A^2^, which is not recognized clinically, may perpetuate PSC activation. In a healthy pancreas, PSC store a metabolite [retinoic acid (RA)] of vitamin A (retinol). When activated, in cancer or inflammation, PSC lose RA stores and assume an activated myofibroblast phenotype^1^. Furthermore, RA also is a vital molecule regulating key signaling pathways guiding embryonic pancreas development^3,4^; signaling cascades that are hijacked during pancreatic carcinogenesis.

Based on these observations, we demonstrated, using various PDAC models, that restoring RA depots within PSC, using all-*trans* retinoic acid (ATRA), limited the desmoplasia and suppressed cancer growth^1,5–7^. Furthermore, we established that activated PSC impede the migration of immune cells, such as CD8^+^ T cells, natural killer, and B cells, into the immediate PDAC microenvironment; a process that was reversed by ATRA^5^. ATRA is an ideal agent to dampen multiple, amplified, embryonic, context-specific signaling cascades activated in PDAC^7,8^. ATRA, but not 9-*cis*- or 13-*cis*-retinoic acid, reduces PSC proliferation by G1 cell-cycle arrest with accumulation of lipid droplets, thus restoring their normal physiological role. Specificity of retinoid (RAR) and rexinoid (RXR) receptor isoforms, distinctly used and regulated by various RA, is vital in pancreatic embryogenesis and PSC biology^9^. Our data suggest a specific upregulation of RARβ isoform by ATRA^7^. This is relevant, since 13-*cis-*RA has previously been found to be ineffective, in combination with either gemcitabine^10^ or interferon^11^ in patients with PDAC.

Since only ATRA is relevant to PSC physiology and embryonic development of the pancreas, here we re-purpose ATRA as a stromal-targeting agent, in combination with one of the widely used standard-of-care chemotherapy^12^ in a phase Ib clinical trial. We demonstrate that ATRA is a stromal targeting agent by conducting pharmacokinetic and pharmacodynamic studies to discover specific biomarkers while determining recommended phase 2 dose (RP2D).

## Results

### Trial design and enrollment

**Fig. 1:**
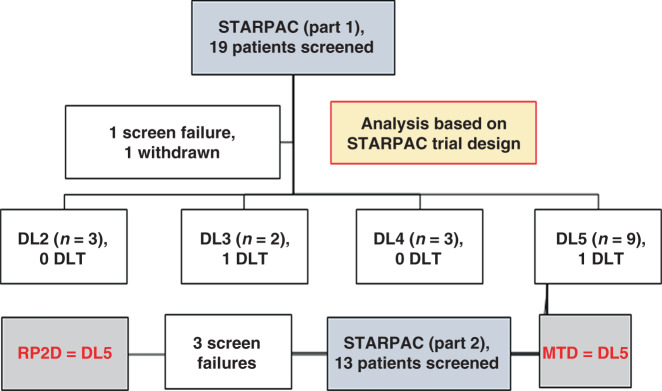
CONSORT diagram for STARPAC clinical trial. Number of patients at all dose levels (DL) in dose escalation, part 1 (using the STARPAC adaptive trial design^13^) for maximum tolerated dose (MTD) estimation, showing dose-limiting toxicity (DLT) and dose expansion (part 2) of the trial for optimal biological dose (OBD) estimation leading to recommended phase 2 dose (RP2D).

We used an innovative two-step, adaptive, Bayesian continual reassessment method using five potential dose levels (DL) which appears to have advantages over standard 3 + 3 and titeCRM designs in accurately predicting RP2D, based on priors of toxicity data^13^ (Fig. 1, Supplementary Fig. 1, and Supplementary Table 1). A total of 32 patients were screened to enroll 28 of whom 27 received any treatment from February 2016 to February 2018. Final data collection cut-off for clinical parameters was 1 April 2019.

### Primary and secondary outcomes

**Fig. 2:**
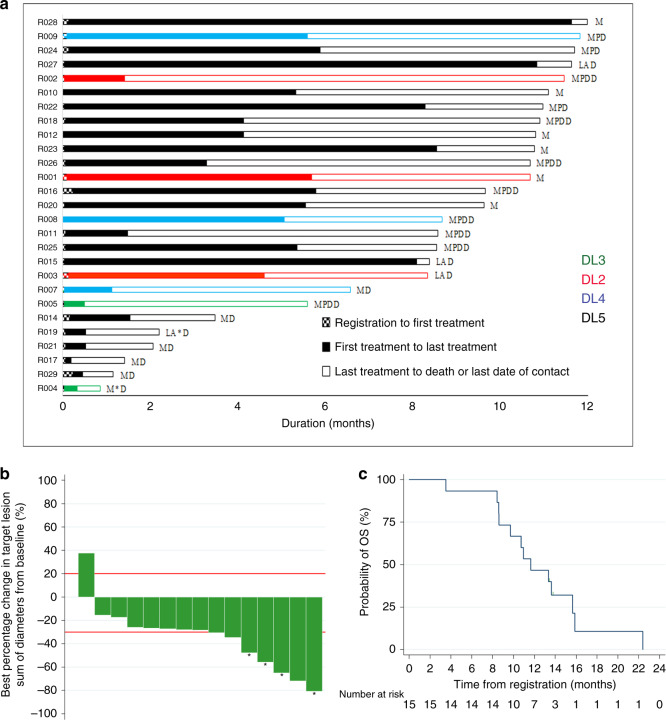
Primary and secondary endpoints for STARPAC clinical trial. **a** Swimmer’s plot with color code for different dose levels (DL) and duration (months) on *X*-axis along with type of disease: locally advanced (LA) and metastatic (M), those who experienced DLT (*) and disease status (Death (D), progressive disease (PD)) censored at the pre-specified 12 months of starting on the trial. **b** Waterfall plot of best percentage change of sum of diameters in target lesion from baseline in RP2D treated patients based on an evaluable population. A positive change denotes an increase in the target lesion sum of diameters over time and, likewise, a negative change denotes a decrease in the target lesion sum of diameters over time. Reference lines added for response (−30% change in target lesion sum of diameters) and progression (20% change in target lesion sum of diameters). RECIST responses are marked with asterisk (*). There was progression for 6.7% (95% CI: 0.2–31.9%) and response in 46.7% (95% CI: 21.3–73.4%) of patients. **c** Post hoc (including data from beyond 12 months) estimated median overall survival in 15 patients receiving RP2D on evaluable population basis. Number of events = 13. Kaplan–Meier plot.

We demonstrated that the FDA/EMEA-approved doses of gemcitabine (G, 1000 mg/m^2^ iv) and nab-paclitaxel (nP, 125 mg/m^2^ iv), both on days 1, 8, and 15 of each 28-day cycle (PDAC^12^), can be combined safely with the recommended dose of ATRA (for acute promyelocytic leukemia, APML^14^) at 45 mg/m^2^ orally in two divided doses from days 1 to 15 of each cycle in patients with PDAC, resulting in an acceptable toxicity and side-effect profile (DL5, Table 1, Figs. 1 and 2, and Supplementary Tables 1–5). Thus, the primary outcome of maximum tolerated dose (MTD) and RP2D was DL5. Two patients had dose-limiting toxicities of grade 4 thrombocytopenia (one patient each at DLs 3 and 5). Among secondary outcomes on safety and tolerability, neurotoxicity, characteristically seen with nab-paclitaxel treatment, appeared to be reduced by ATRA, in frequency and intensity, an aspect to be explored in larger randomized studies. This feature was previously reported in the context of lung cancer^15^, although with no underlying mechanistic explanation^16^.

**Table 1:** Adverse events for STARPAC clinical trial.

Adverse events (AE) summary		
	DL5 patients	All patients
Total patients (*N*)	19	27
AEs reported, *n*	470	638
Patients with at least one AE, *n*	19	27
AEs per patient^a^, median (range)	23 (8–71)	23 (8–71)
≥Grade 3 AEs^a^ reported, *n*	33	55
Patients with at least one ≥Grade 3 AE, *n*	11	17
≥Grade 3 AEs per patient^a^, median (range)	3 (1–5)	3 (1–9)

Distribution of AE. AE ≥ grade 3 and DLTs in all patients and those receiving DL5 (RP2D) according to SOC term, whether attributable or not to treatment. *N* = number of patients in the Safety Set population for the specified group of patients.

^a^Counts of each instance, e.g., if one patient has the same term three times this is counted as three instances.

Furthermore, patients treated at the MTD demonstrated encouraging evidence of response when assessed by best response of change in the target lesion sum of diameters compared to baseline (Fig. 2b). Median progression-free survival (PFS) was 6.4 months (95% CI, 3.5 months–not reached (NR)) and median overall survival (OS) was 10.9 months (95% CI, 8.6 months–NR) (Supplementary Table 4) in the evaluable population (receiving at least two cycles of this combination or progressing within the first two cycles, *n* = 15) analysis restricted to pre-specified follow-up for 12 months only for RP2D. Post hoc analysis of these patients (*n* = 15), of data beyond 12 months, showed that the median OS of 11.7 months (95% CI, 8.6–15.7 months) for RP2D may be superior to the reported (8.5 months, 95% CI: 7.9–9.5 months) for metastatic PDAC in the phase III clinical trial with gemcitabine–nab-paclitaxel^12^. Additionally, four of these patients (27%) went on to have second-line treatment (FOLFIRINOX, FOLFIRI, FOLFOX, 5FU + liposomal irinotecan (*n* = 1 for each)), which was a lower proportion when compared to the pivotal phase III trial (38–42%)^12^. Accepting that this is an early phase I trial, these are promising results.

### ATRA pharmacokinetics

**Fig. 3:**
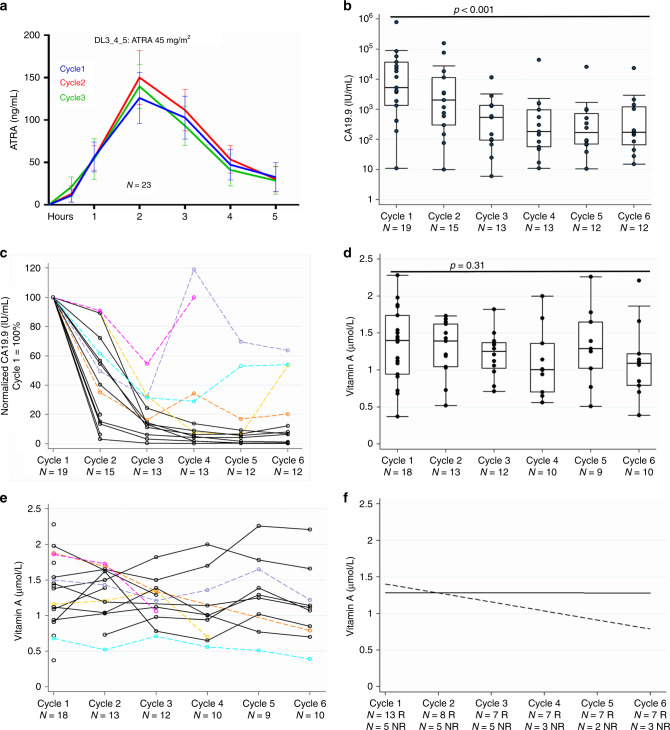
ATRA pharmacokinetics, biochemical CA19-9 response and vitamin A levels. **a** Serum ATRA levels for the first three cycles are summarized as mean (SEM) for the first 5 h after co-administration of ATRA at 45 mg/m^2^ with chemotherapy drugs. **b** Absolute CA19-9 levels on logarithmic *Y*-axis for patients on dose level 5 at start of each cycle. Summary statistics represented by box (median ± interquartile range) and whisker (range: LQR−(1.5 × IQR) and UQR + (1.5 × IQR)). Two-sided Skilling–Mack test, statistic 39.21, *p* < 0.001. **c** Normalized CA19-9 levels for each patient on dose level 5 with baseline being 100%. There were 14 biochemical responders (black) compared to 5 non-responders (unique colors). Responders are defined as those who show >30% reduction of CA19-9 from baseline with a sustained response (no greater than 20% rise from previous reading at any time). **d** Vitamin A on dose level 5 at start of each cycle. Summary statistics represented by box (median ± interquartile range) and whisker (range: LQR−(1.5 × IQR) and UQR + (1.5 × IQR)). Two-sided Skilling–Mack test, statistic 5.95, *p* = 0.31. **e** Individual values for vitamin A for patients with biochemical non-responders (CA19-9) highlighted in corresponding colors as in panel **c**. **f** Linear regression trend lines comparing biochemical responders (solid line) to non-responders (dashed line) demonstrate that a drop in serum vitamin A levels may indicate non-responders. *N* = *X* R: *X* is the number of responders at the stated cycle. *N* = *X* NR: *X* is the number of non-responders at the stated cycle.

The addition of chemotherapy did not reduce plasma levels of ATRA (Fig. 3a, Supplementary Figs. 2 and 3, and Supplementary Tables 6 and 7) when compared to historical data of single-agent ATRA at similar doses in patients without PDAC^17,18^. The ATRA regimen at RP2D (at a dosing and schedule optimized for APML^19^) resulted in consistent plasma ATRA concentrations (AUC and *C*_max_) during successive cycles, strongly suggesting lack of CYP26 enzyme induction, a key factor limiting continued dosing with ATRA^20^. Patient compliance with scheduling was excellent, with better median dose intensities of both cytotoxic agents than previously reported in the phase III trial for gemcitabine–nab-paclitaxel (Supplementary Table 5)^12^.

### Biochemical response and vitamin A levels

CA19-9 biochemical responses are a reliable predictor of long-term survival in PDAC^12,21^. While most patients (14/19 at RP2D) showed significant early and sustained CA19-9 responses, we identified five patients who had a poor CA19-9 response (Fig. 3b, c and Supplementary Fig. 4). Plasma vitamin A levels for most patients were maintained throughout all cycles, implying no induction of CYP26 enzyme-mediated clearance. The five patients who had poor CA19-9 responses exhibited either a lower starting or a decline in levels of plasma vitamin A during course of therapy, which upon linear regression analysis demonstrated a downward trend as opposed to steady levels for patients with a biochemical response (Fig. 3d–f and Supplementary Fig. 5). This implies that plasma vitamin A levels could potentially be a surrogate pharmacodynamic marker as a composite readout of absorption and metabolism of retinol which is upstream of, and therefore distinct from, ATRA absorption and metabolism. An added benefit is the convenience of a routine assay in hospital laboratories. Optimal biological dose (OBD) using vitamin A levels ≥1 and ≤2.5 µmol/L, on a exploratory basis^22^, was achieved in 67–82% of patients in each of the cycles (cycle 1 (72%, *n* = 18), cycle 2 (82%, *n* = 11), cycle 3 (82%, *n* = 11), cycle 4 (67%, *n* = 9), cycle 5 (75%, *n* = 8), and cycle 6 (67%, *n* = 9)) for DL5 patients whom vitamin A levels were available and excluding any patients who had dose modifications from the point of modification onwards (Supplementary Table 8). These data led to ATRA dosing, as described in DL5, to be taken forward as RP2D.

### Exploratory biomarkers: diffusion-weighted magnetic resonance imaging (DW-MRI)

**Fig. 4:**
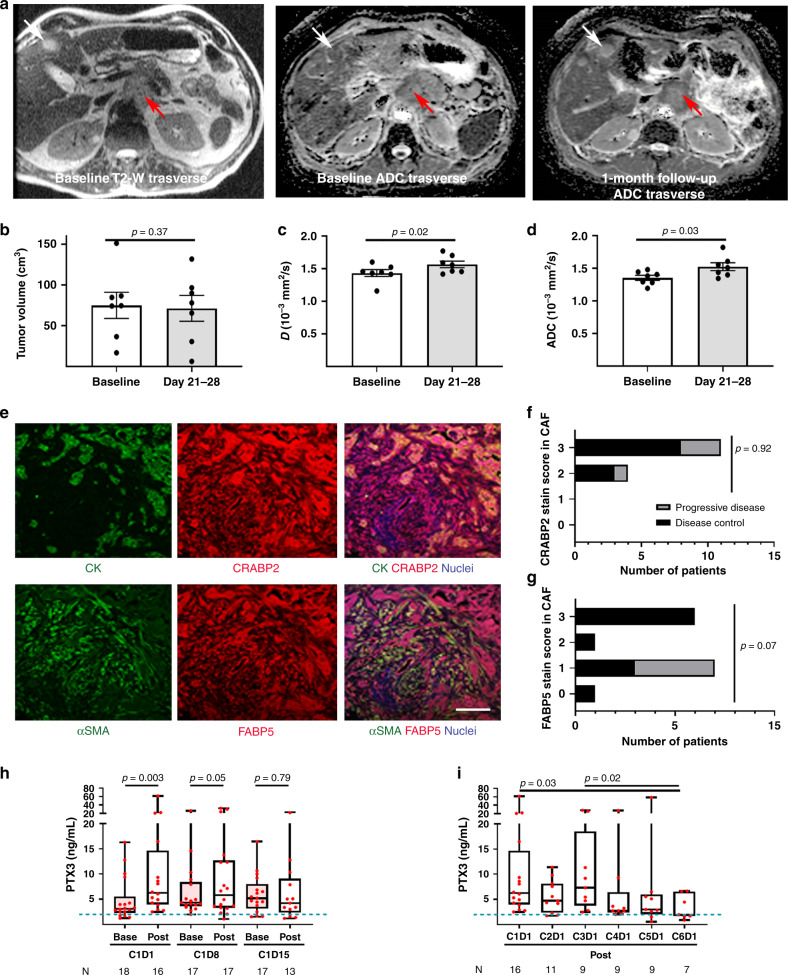
Biomarkers for STARPAC clinical trial. **a** MRI sequences as indicated with primary pancreatic tumor (red arrow) and liver metastasis (white arrow) in T2-weighted images for localization, both lesions demonstrating change in the apparent diffusion coefficient (ADC) within these tumors, after just 1 month of treatment, indicative of an increased mobile water content due to reduction in dense cellularity with a rim of peripheral restricted tissue represents residual tumor. Summary statistics of changes in tumor volume (**b**), ADC values (**c**), and *D* values (**d**) between pre-treatment (baseline) and post-first-cycle (days 21–28) of treatment. Summary data as mean ± SEM. Data points represent values from individual patients. Two-tailed Wilcoxon matched-pairs sign-rank test. **e** Representative images of co-immuno-fluorescent images of pancreatic cancer biopsies prior to commencement of treatment, to assess prevalence of cellular retinoic acid-binding protein 2 (CRABP2) and fatty acid-binding protein 5 (FABP5), as indicated with co-staining with either cytokeratin (CK) or alpha-smooth muscle actin (αSMA) respectively, to demonstrate a 3+ stain for both CRABP2 and FABP5 in cancer cells and cancer-associated fibroblasts (CAF). Scale bar: 100 µm. **f**, **g** These quantifications (range 0 to 3+) were then assessed for all evaluable biopsies (*n* = 15) from single representative image using appropriate tissue controls and categorized according to disease control/progressive disease. Chi-square test. d.f. = 3. **h** Measurement of serum PTX3 by ELISA (GCLP standards, CV 0.17) in patients before (Base) and 5  h after (Post) taking ATRA in the first cycle (C1) on days 1, 8, and 15. **i** Measurement of serum PTX3 5 h after taking ATRA on the first day of each cycle (1–6). Each point represents an individual patient. **h**, **i** Box (median ± interquartile range) and whisker (full range). Individual measurements per patient represented as a dot derived from mean of two readings. Two-tailed Wilcoxon matched-pairs signed-rank test.

Several potential biomarkers have emerged from this phase I study which might be employed in future studies. Since repeated biopsies of primary tumors are not practical and may be ethically demanding, we used an imaging biomarker as a surrogate for stromal activity. The dense cellular stroma reduces tissue water content. DW-MRI allows derivation of an apparent diffusion coefficient (ADC), which reflects extra- and intra-cellular water mobility^23^, and can be a robust imaging biomarker for response assessment in human tumors, if appropriately protocoled for cross-platform analysis (Supplementary Tables 9 and 10 and Supplementary Fig. 6)^24^. Hence we evaluated ATRA’s stromal effect using the true diffusion (*D*) component of ADC. True diffusion (*D*) values demonstrated a consistent increase as early as 1 month after treatment, indicating stromal modulation, where there is no change in tumor volume, indicating stromal modulation^25^, as observed in our preclinical models^7^ (Fig. 4a–d).

### Exploratory biomarkers: tissue assays

The baseline biopsies assessed for RA transport molecules in cancer and stromal cell compartments, demonstrated a differential distribution of fatty acid-binding protein 5 (FABP5) and cellular RA-binding protein 2 (CRABP2)^26^, using a well-validated method to distinguish stromal and epithelial compartments^27^, such that patients with increased stromal expression of FABP5 were more likely to achieve disease control (Fig. 4e–g, Supplementary Figs. 7 and 8, and Supplementary Table 11). Thus the stromal expression of FABP5 can be explored as a potential predictive biomarker.

### Exploratory biomarkers: serum assays

Since we demonstrated previously that pentraxin 3 gene (*PTX3*) is upregulated in activated PSC, and can be used as a potential diagnostic biomarker for pancreatic cancer^28^, we explored whether serum PTX3 could act as a stromal-response biomarker. An upregulation of serum PTX3 within 5 h of administering ATRA on days 1 and 8 of the first cycle, an effect lost by day 15, indicated that continuous administration of ATRA may not have a sustained stromal effect (Fig. 4h and Supplementary Fig. 9). Upregulation of serum PTX3 was not demonstrable by cycle 6 of the treatment when the median serum PTX3 was at the upper limit of normal, as seen in healthy subjects, perhaps indicating the maximum duration of ATRA therapy should be 6 months (Fig. 4i). Serum PTX3 will be explored further in the context of a planned randomized controlled trial where comparisons can be made with non-ATRA treated patients.

## Discussion

The promising results of this phase I study support stromal normalization as a valid approach in chemotherapeutically intractable cancers such as PDAC. We demonstrate that repurposing ATRA as a stromal-targeting agent, with gemcitabine–nab-paclitaxel, is safe and tolerable with an exciting potential to enhance delivered chemotherapy dose intensity, and mitigating some of the expected adverse events, such as neurotoxicity, with evidence of putative pharmacodynamic readouts. These features are in contrast to recently publicized negative results of HALO 109-031 trial targeting stroma using pegvorhyaluronidase alfa (PEGPH20), an agent which potentially increases adverse events^29^.

This translation of preclinical work to a clinical application, based on clinical observations and repurposing existing drugs, should be tested in other diseases where stromal normalization could impact clinical outcome. Based on the encouraging response and survival data seen here, the efficacy of this regimen will be evaluated in a phase II randomized clinical trial in locally advanced PDAC (NCT04241276), incorporating pharmacodynamic biomarkers for ATRA and stromal targeting, and genomic readouts of tumoural^30^ and stromal^31^ heterogeneity, which may play role in differential response.

## Methods

### Trial design and patient population

STARPAC was an open-label, multicenter, phase Ib study of ATRA administered with gemcitabine and nab-paclitaxel in patients with locally advanced or metastatic pancreatic cancer, who had not received prior systemic therapy for their disease. Additional eligibility criteria included World Health Organization (WHO) performance status 0 or 1, life expectancy ≥12 weeks, and adequate hematologic and end-organ function within 14 days prior to the first study treatment. Major exclusion criteria were known brain metastases, pre-existing sensory neuropathy (>grade 1) and serious medical risk factors involving any major organ systems, or serious psychiatric disorders, which could compromise the patient’s safety or the study data integrity.

There were two parts to this study. In Part 1, a dose-escalation strategy using the two-step adaptive Bayesian continual reassessment method (CRM)^13^ was used to determine the MTD and the recommended dose to be taken forward in Part 2, a dose expansion phase, to explore the OBD. OBD initially defined by vitamin A levels between 1.5 and 2.5 μM (both inclusive) at each cycle was post-trial closure modified to levels of 1 and 2.5 μM (both inclusive) in line with National Institute of Health’s Office of Dietary Supplements’ recommended levels^22^. OBD was estimated at 80% of patients achieving serum vitamin A levels.

All patients provided written informed consent. Ethical approval for STARPAC clinical trial: South Central-Berkshire Research Ethics Committee (REC); 15/SC/0548 dated 13 October 2015 (Supplementary Note 1: Trial Protocol). STARPAC trial was prospectively registered with EudraCT (2015-002662-23) on 11 June 2015 and clinical trial.gov (NCT03307148) on 11 October 2017. Trial opened to recruitment on 20 January 2016. Three substantial amendments were made to clinical trial protocol, and details are available on EudraCT. Permission for post hoc analysis for data beyond 12 months of study was obtained from South Central-Berkshire REC on 24 July 2019. All clinical data were collected on an in-house built electronic Case Report Form (eCRF) designed using ORACLE v11.2.0. The study was sponsored by Barts Health NHS Trust. The Centre for Experimental Cancer Medicine (CECM), Barts Cancer Institute, Queen Mary University of London had overall responsibility for trial management. The Trial Management Group (TMG) was responsible for day-to-day running of the trial. Safety data were reviewed regularly by the Safety Review Committee (SRC).

### Statistical analysis

It was expected that a maximum of 24 evaluable patients would be enrolled into Part 1 of the study based on CRM^13^. For Part 1, the primary objective was to determine the MTD of the combination of gemcitabine–nab-paclitaxel and ATRA, measured by the occurrence of DLTs during the first 28 days of treatment that were attributed as possibly, probably, or definitely related to the study treatment. For Part 2, a sample size of 10 was considered reasonable to provide indicative data on OBD.

Secondary endpoints included analyses of PK parameters, response rates, PFS, OS, and safety. For all time-to-event analyses performed, patients who did not have an event were right censored: PFS censored on the last date the patient was known to be progression free; OS censored at the date of last contact within 12 months of enrollment into trial. Post hoc OS analysis was carried out for data beyond 12 months after REC approval to include data as there were exceptional survivors. Survival endpoints were shown graphically with Kaplan–Meier plots.

All efficacy analyses were performed on the evaluable population which included all patients receiving at least two cycles of the combination or progressing within the first two cycles, regardless of whether they were later found to be ineligible or a protocol violator. Safety analyses included all patients who received at least one dose of study treatment. The worst grade of each adverse event (AE) for each patient during study treatment was reported. Cumulative dose intensity over the first six cycles was calculated as the actual amount of study drug received over the first six cycles divided by the expected amount of study drug received over the first six cycles. The expected amount of study drug was calculated based on the dose and schedule specified in the study protocol.

Sample size calculations were performed using the software package PASS version 12.0. All clinical efficacy endpoints were analyzed using STATA version 13.1. Laboratory data were analyzed using PRISM (GraphPad Inc) version 8. Statistical tests are described as used.

### Sample storage and traceability

All samples had a valid chain of custody throughout procurement, temporary storage at site, shipping, and permanent storage at the Barts Pancreas Tissue Bank (BPTB, REC Ref: 13/SC/0592, HTA License number: 12199), and were given to laboratory staff via a traceable database, in a blinded, anonymized manner.

### Pharmacokinetic assays

ATRA, 9-*cis*-RA, and 13-*cis*-RA were purchased from Sigma Aldrich (Poole, UK), and ATRA-d5 from Toronto Research Chemicals (North York, Ontario, Canada). Liquid chromatography mobile phase solvents (water, acetonitrile, and formic acid) were Optima grade, purchased from Fisher Scientific (Loughborough, UK). An analytical method, using liquid chromatography tandem mass spectrometry (LC-MS/MS), was established for the measurement of ATRA concentrations in plasma. The method was subject to EMEA validation procedures^32^. Validation of this method included precision and accuracy, selectivity, specificity, matrix effects (including hemolytic and hyperlipidemic plasma), effect of co-medications, carryover, re-injectability, stability in whole blood, stability of stock and working solutions and stability assessments in plasma (24 h room temperature, 4 freeze/thaw cycles, and long-term frozen storage at both −20 and −80 °C).

All analyses were done using an AB Sciex 6500 mass spectrometer (Warrington, UK) equipped with a Nexera 2 LC-system (Shimadzu, MA, USA). Internal standard ATRA-d5 was added to 10 µL of patient plasma sample. The plasma proteins were precipitated using acetonitrile, followed by vortexing and centrifugation. Supernatants (150 µL) were transferred to clean wells in a 96-well plate, followed by the addition of 50 µL water and vortex mixing. Calibration standards (calibration range 50–5000 ng/mL) and QC samples (100, 300, 800, and 4000 ng/mL) were prepared by the addition of ATRA to blank human plasma, and then processed in the same manner as patient samples (Supplementary Fig. 2 and Supplementary Table 7).

The extracts were analyzed by reversed phase chromatography (Acquity BEH C18 UPLC column, Waters Corp., MA, USA), using gradient elution with acetonitrile and 0.1% formic acid, at a flow rate 0.4 mL/min, total run duration 10 min. This was coupled to the MS/MS detector, operating in positive ion atmospheric pressure chemical ionization mode. The MS/MS transitions for ATRA and internal standard ATRA-d5 were *m*/*z* 301 > 205 and *m*/*z* 306 > 206, respectively. The declustering potential, collision energy, and collision exit potential were 40, 20, and 12 V, respectively, both for ATRA and ATRA-d5. A minimum of six quality control samples were included in each LC-MS/MS run. ATRA calibration standards were prepared in the calibration range 50–5000 ng/mL using pooled human plasma. Standards and samples were assayed in the same manner using the internal standard ATRA-d5. Quality control samples at 100, 300, 800, and 4000 ng/mL were used to determine accuracy and precision (Supplementary Fig. 2 and Supplementary Table 7).

Pharmacokinetic data were calculated using Prism software (GraphPad) and validated against PCModfit software (http://pcmodfit.co.uk/nca.html) with substitution of all “below limits of quantification” levels of ATRA at zero (range could be zero to 62.5 ng/mL).

### Diffusion-weighted magnetic resonance imaging

MRI was performed at two institutions on multivendor platforms (Supplementary Table 9). Longitudinal studies on the same patient were undertaken on the same scanner. The protocols were developed to maximize signal-to-noise ratio and minimize ghosting and distortion using a well-validated test object^33^.

Scans were done at baseline and at days 22–28 after treatment. Test object measurements for quality assurance of quantitative metrics were undertaken regularly to ensure quality control. The coefficient of variation (CV) for ADC across multiple time-points was 0.4% and 1.4% (Philips and GE, respectively). ADC CV between the two sites was 3.9%. MRI examinations consisted of DW-MRI of the abdomen and pelvis as per the protocols below, followed by T_1_-weighted and T_2_-weighted imaging in matched positions.

### DW-MRI analysis

On the pre- and post-treatment scans, regions-of-interest (ROI) were drawn around the tumor on the high *b*-value (*b* = 800 s/mm^2^) diffusion-weighted images by a board-certified radiologist in OsiriX version 9.0. ROIs were then copied onto the corresponding ADC maps which were generated on a voxel-by-voxel basis from a mono-exponential fit to the data as described by *S*(*b*) = *S*(*0*) exp(−*b* × ADC)^24^.

### Tissue CRABP2, FABP5

Hematoxylin and eosin quality control of remaining tissue from diagnostic material by a board-certified pathologist for histological/cytological confirmation demonstrate that there was either inadequate tissue (*n* = 7) or only cancer cells without stroma (*n* = 5) to carry further analysis. Hence formalin-fixed paraffin-embedded sections from 15 patients were dewaxed and rehydrated, antigen retrieved (0.1 M citrate buffer, pH 6, microwave, 20 m), blocked (1 h, RT, 2% bovine serum albumin, 0.02% fish skin gelatin, 10% FBS, 5% goat serum) before use of primary antibodies at 4 °C overnight, followed by appropriate fluorescent-labeled secondary antibodies^27^. The nuclei were then counterstained with DAPI. Organotypic sections, from as previous experiments^7^, were used for positive and negative staining controls (Supplementary Table 11). Controls were uniformly negative with appropriate isotype-specific immunoglobulin at matching dilutions.

Immunofluorescent images were taken using the Zeiss Confocal LSM510 microscope at ×20 magnification, and images were visualized using Zeiss Zen 2.3 software. The green channel represented either α-smooth muscle actin or cytokeratin; the red channel represented FABP5 or CRABP2. The intensity of fluorescence in the green/red channel for respective molecules was given a semi-quantitative value, ranging from negative “−” through to strongly positive “+++”. There were four categories of fluorescence intensity: −, +, ++, +++. The threshold gain and offset was set according to the intensity of the green/red channel in the organotypic cultures, and ensured minimal inter-day variability (Supplementary Figs. 7 and 8).

### Plasma PTX3

PTX3 levels were quantified with a sandwich enzyme-linked immunosorbent assay (ELISA) using in-house validated protocol based on a monoclonal antibody MNB4 (Enzo Life Sciences ALX-804-464-C100)^34^. Plasma PTX3 concentrations were quantified using the sandwich ELISA as follows: 96-well-ELISA plates were coated with MNB4 anti-human PTX3 antibody (100 ng/well) diluted in coating buffer (15 mM carbonate buffer, pH 9.6, overnight, 4 °C), washed, blocked (5% dry milk in washing buffer, 2 h, room temperature), washed, and incubated with either 50 μL of diluted plasma (1:3 dilution in PBS without Ca^2+^ Mg^2+^ and 2% BSA) or 50 μL recombinant human PTX3 standards (0.31–20 ng/mL), all in duplicates for 2 h at 37 °C. After two washes, 50 ng/mL of biotinylated PTX3 (Enzo Life Sciences, cat. ALX-210-365B) antibody was added in each well for 1 h at RT, washes and color realized by 100 μL/well streptavidin-horseradish peroxidase (Amersham, cat. RPN4401V) diluted 1:4000 for 1 h at RT. After further washes 100 μL of chromogen substrate (ThermoFisher cat. 34028B) was added and plates were read after 15 min at 450 nm in a plate-reader. Polynomial regression graphs were constructed for standard curves. Plasma samples of each patient time-point were thawed only once and assayed, maintaining a chain of custody, in duplicate. ELISA was conducted in a blinded manner, and inter-day variability standard patient samples were used with CV 0.17 (Supplementary Fig. 9). Patient variables were unblinded after submission of readouts.

### Reporting summary

Further information on research design is available in the Nature Research Reporting Summary linked to this article.

## Supplementary information

Supplementary Information

Peer Review File

Reporting Summary

## Data Availability

The data supporting this Article are available within the Article, Supplementary Information, or available from the authors upon request.
