# Supplementary material for: Phase I clinical trial repurposing all-trans retinoic acid as a stromal targeting agent for pancreatic cancer
Source: Nat Commun. 2020 Sep 24;11:4841. doi: 10.1038/s41467-020-18636-w (PMC7518421; doi:10.1038/s41467-020-18636-w)
Supplement: Supplementary file 1 — Supplementary Information [file 41467_2020_18636_MOESM1_ESM.pdf]

Phase I clinical trial repurposing all-trans retinoic acid (ATRA) as a stromal targeting agent for pancreatic cancer (STARPAC).

Kocher et al

Supplementary Figures and Tables
and Note (Trial Protocol)

Phase I clinical trial repurposing all-trans retinoic acid (ATRA) as a stromal targeting agent for pancreatic cancer (STARPAC).

Kocher et al

Supplementary Figures

Supplementary Figure 1.

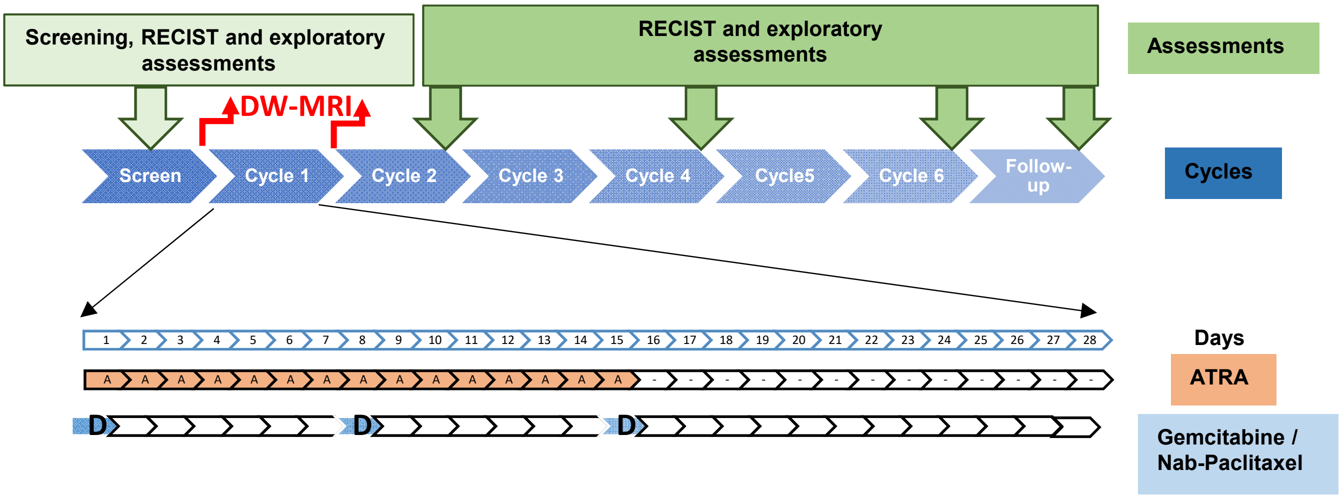

Figure 1: Schema of screening, assessment and treatment schedule for STARPAC trial.
Timeline of screening, treatments and assessments at each cycle which was of 28 days (4 weeks) duration with ATRA self-administered daily for days 1-15 (orange), and gemcitabine-nab-paclitaxel was administered in day-case hospital setting on days 1,8 and 15 of each cycle (blue). First dose of ATRA was administered under supervision at each of the first three cycles at time of start of gemcitabine infusion to obtain serial blood samples. Six such cycles were administered unless there was disease progression. Beyond six cycles ATRA was omitted, but gemcitabine-nab-paclitaxel could be continued till disease progression.

Supplementary Figure 2

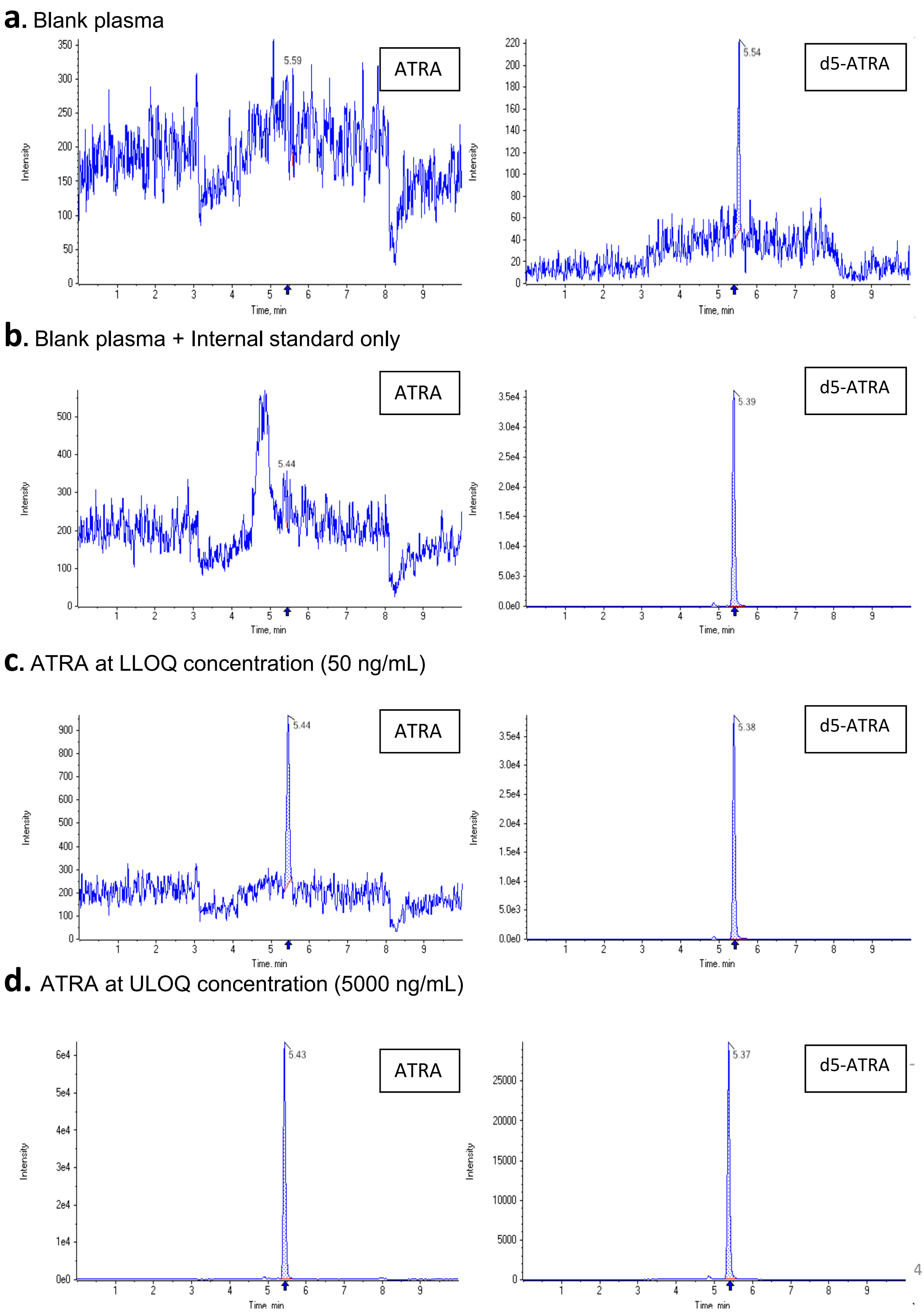

Figure 2: Chromotagrams

Representative multiple reaction monitoring (MRM) chromatograms for ATRA along with D5-ATRA (positive control) with comparisons at the same concentration as at a: Blank plasma. b: Internal standard only. c: ATRA at Lower limit of quantification (LLOQ): 50 ng/mL. d: ATRA at Upper limit of quantification (ULOQ): 5000 ng/mL.

Supplementary Figure 3

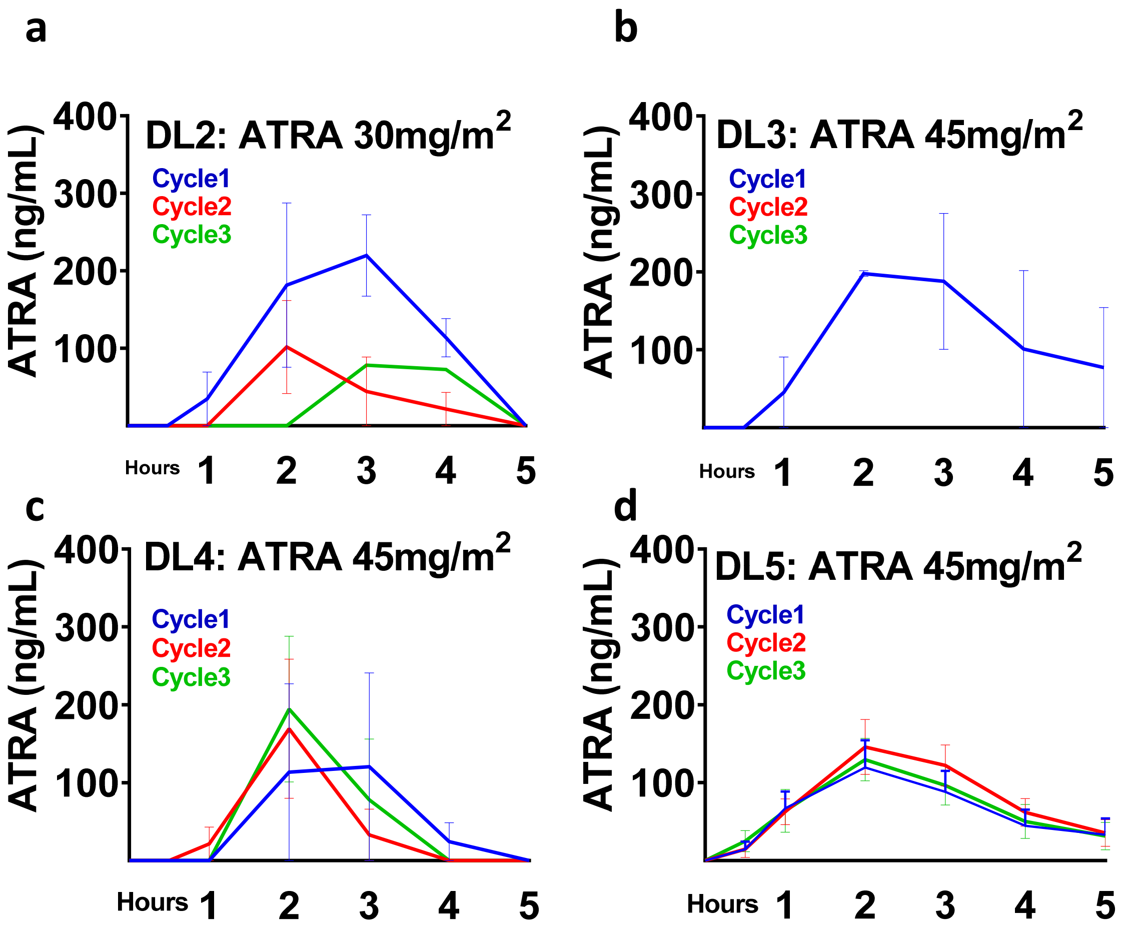

Figure 3: ATRA Pharmacokinetics

ATRA pharmacokinetics for the first three cycles (cycle 1: blue; Cycle 2: red; Cycle 3: green) on Day 1 of each cycle for the first 5 hours after ATRA administration at

a: 30mg/m^2 (Dose Level 2)
b: 45mg/m^2 (Dose Level 3)
c: 45mg/m^2 (Dose Level 4)
d: 45mg/m^2 (Dose Level 5)

Supplementary Figure 4

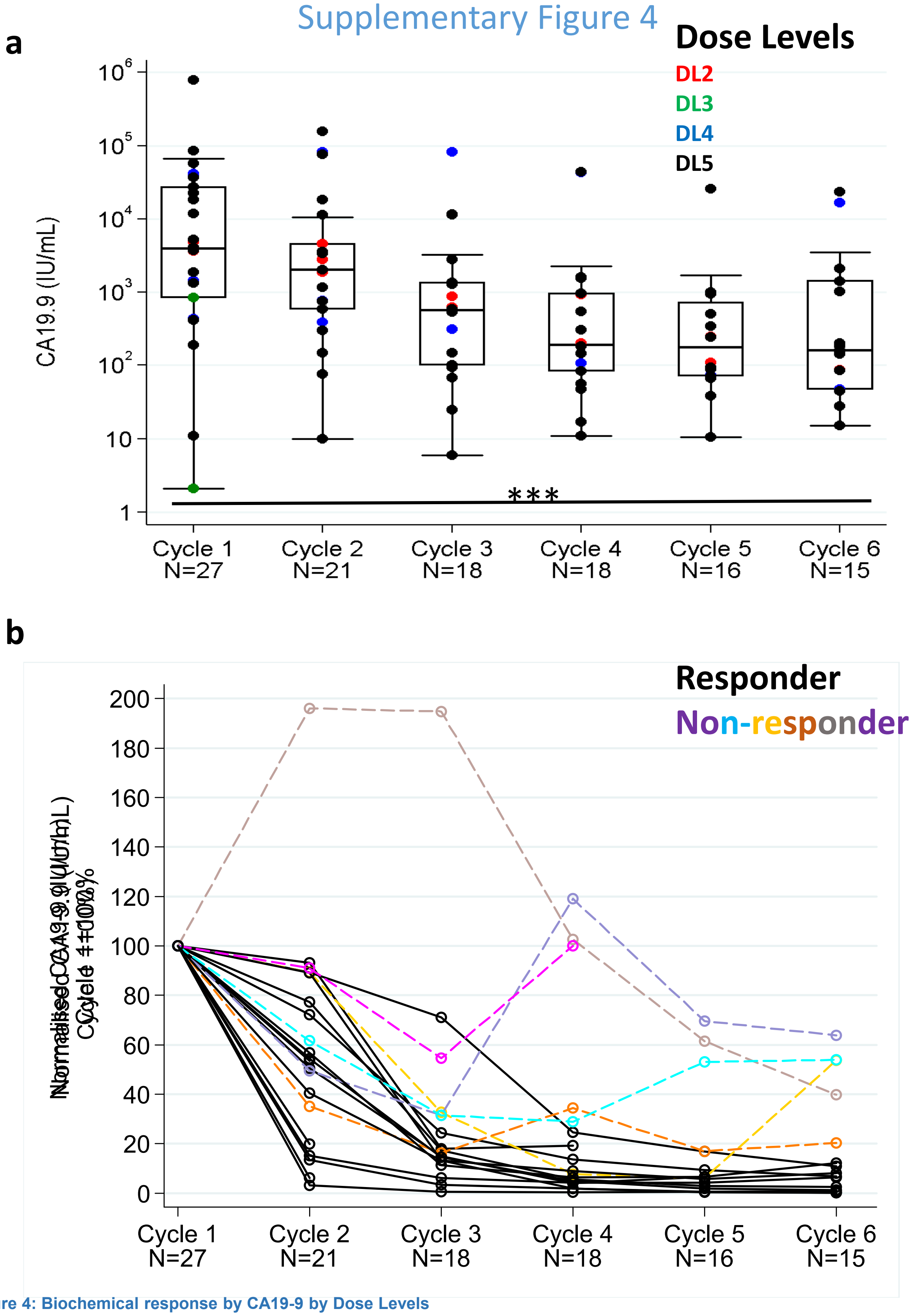

Figure 4: Biochemical response by CA19-9 by Dose Levels

a: Absolute CA19-9 levels on logarithmic Y-axis for patients on Dose Level 2 (red), 3 (green), 4 (blue), 5 (black) at start of each cycle. Summary statistics represented by box (median ± interquartile range) and whisker (range: LQR-(1.5xIQR) and UQR+(1.5xIQR)). One-sided Skilling-Mack test, statistic 52.99, $p < 0.001$.

b: Normalized CA19-9 levels for each patient with baseline being 100%. Biochemical responders are defined as those who show >30% reduction of CA19-9 from baseline with a sustained response (no greater than 20% rise from previous reading at any time). Patients who had an increase in normalized CA19.9 which goes above a value of 20% have been flagged with colored lines. Consequently, there were 15 biochemical responders (black) compared to 6 non-responder (unique colors) of the 21 patients who had data available for more than one cycle.

Supplementary Figure 5

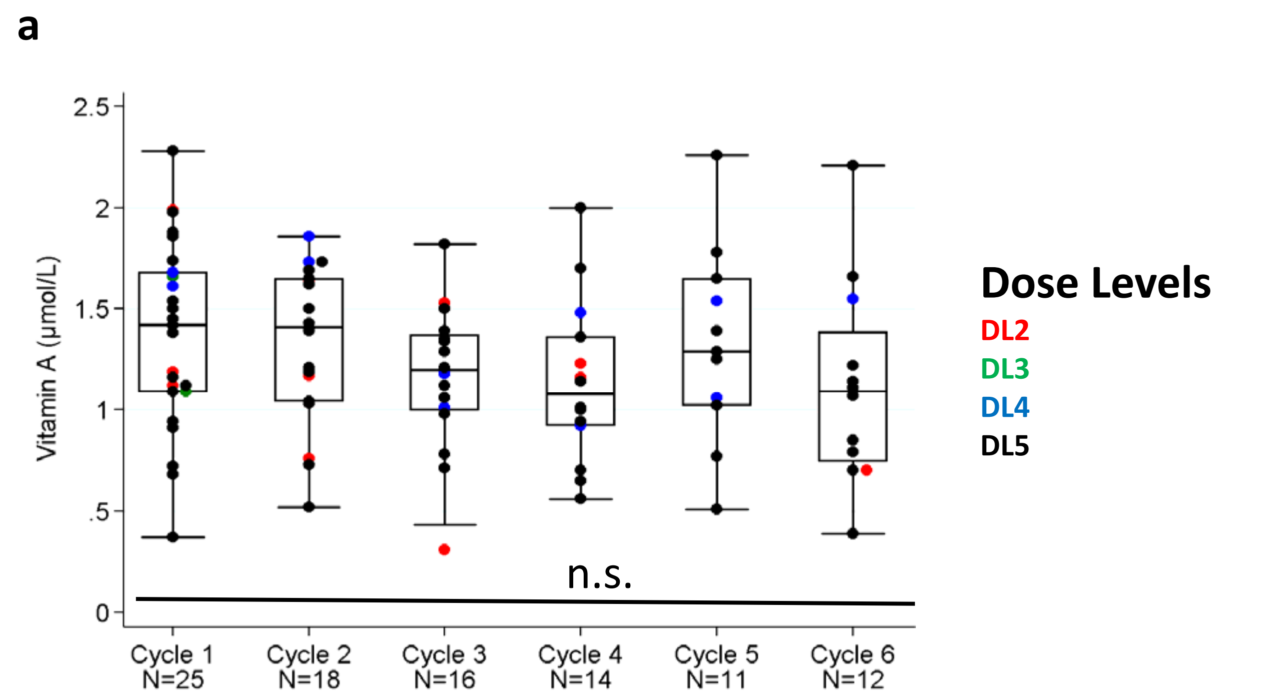

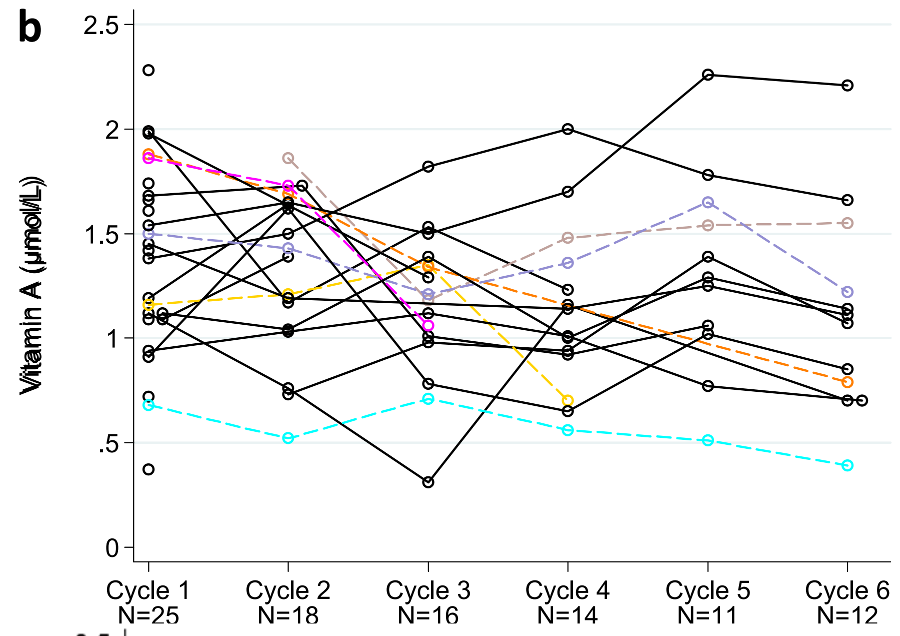

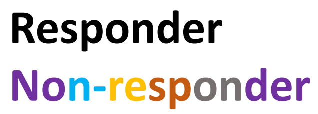

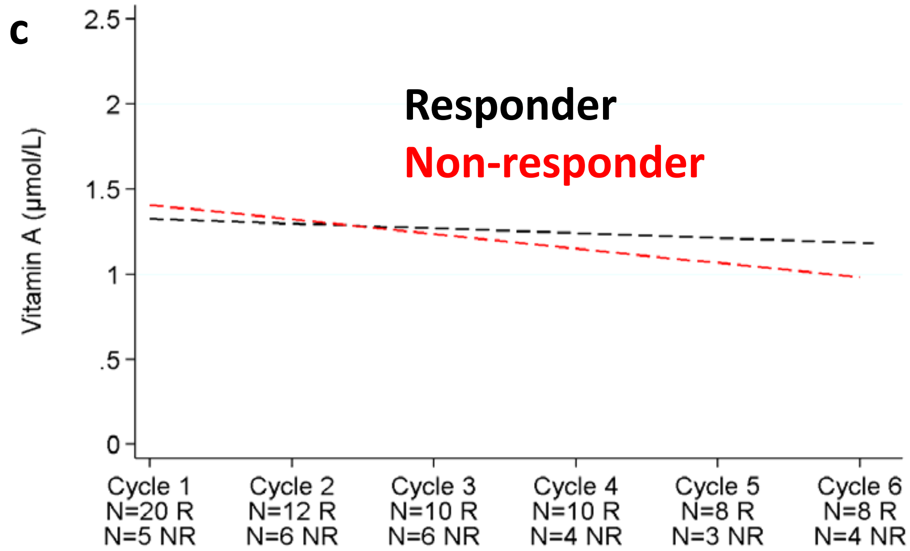

Figure 5: Changes in vitamin A

a: Serum vitamin A levels on Dose Level 2 (red), 3 (green), 4 (blue), 5 (black) at the beginning of each cycle. Summary statistics represented by box (median ± interquartile range) and whisker (range: LQR-(1.5xIQR) and UQR+(1.5xIQR)). One-sided Skilling-Mack test, statistic 6.61, p = 0.25.

b: Individual values for patients with biochemical (CA19-9) non-responders highlighted in corresponding colors as in Supplementary Figure 4b. Responders are defined as those who show >30% reduction of CA19-9 from baseline with a sustained response (no greater than 20% rise from previous reading at any time).

c: Linear regression trend lines comparing biochemical (CA19-9) responders (black) to non-responders (red).

N=X R: X is the number of responders at the stated cycle.

N=X NR: X is the number of non-responders at the stated cycle.

a

Supplementary Figure 6

b

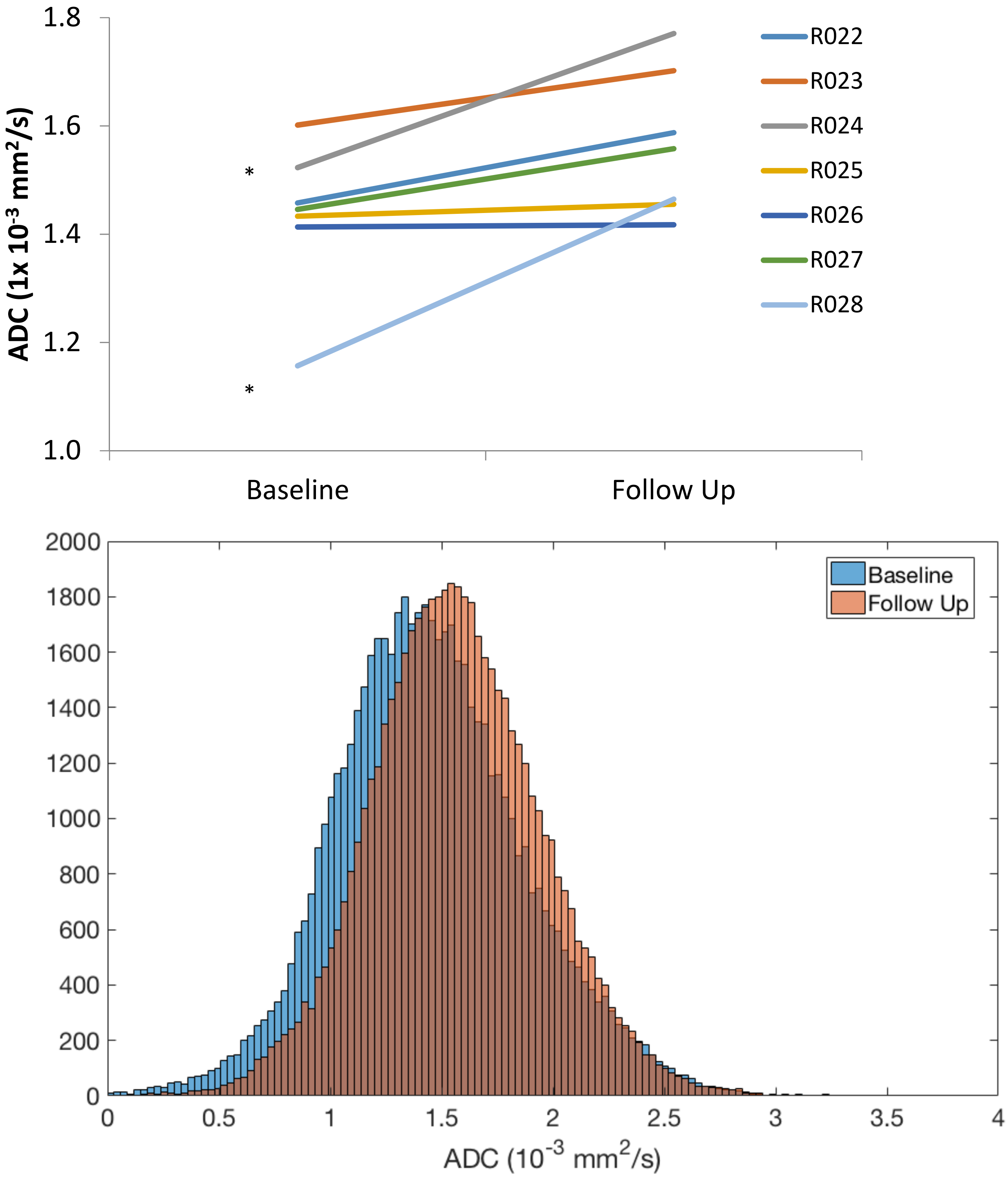

Figure 6: Changes in the ADC values in seven patients with complete data set.

a: Change in ADC values for each individual patient from baseline to follow up (day 21-28). The ADC of 2 patients (R024 and R028) had percentage increases greater than the 95% limits of agreement between them.

b: Histogram plots of ADC showed a right shift of the distribution of histogram parameters towards higher ADC at day 22-28 when the ADC voxel values for all patients were pooled at each time-point. When the ADC voxel values for all patients were pooled to create histograms for ADC at baseline and follow up, the 25th percentile, mean/median and 75th percentile ADC all increased following treatment.

Supplementary Figure 7

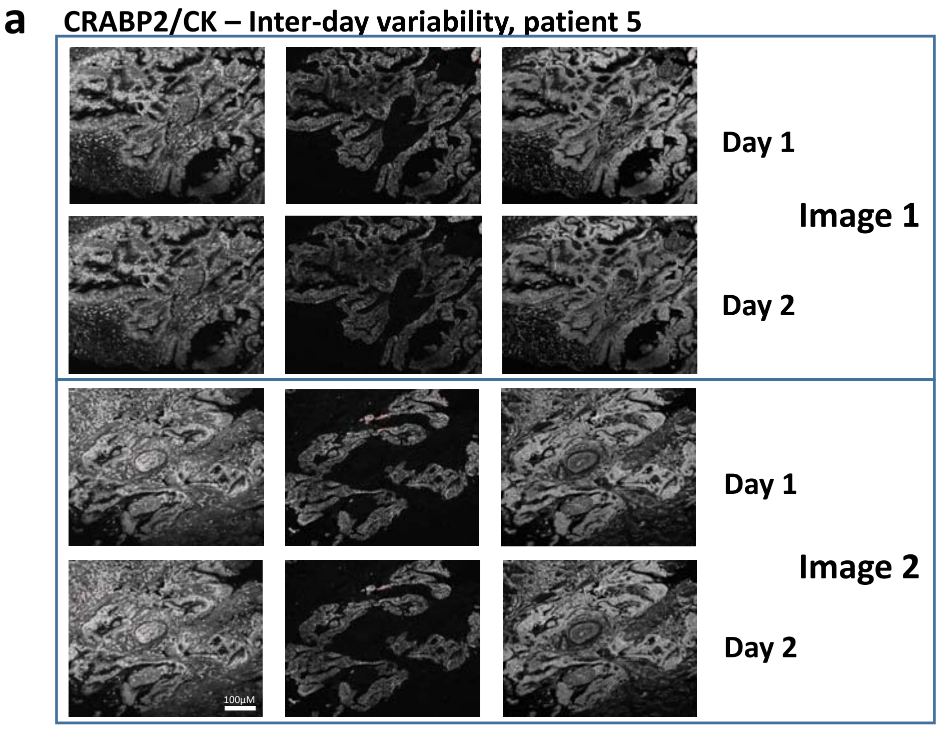

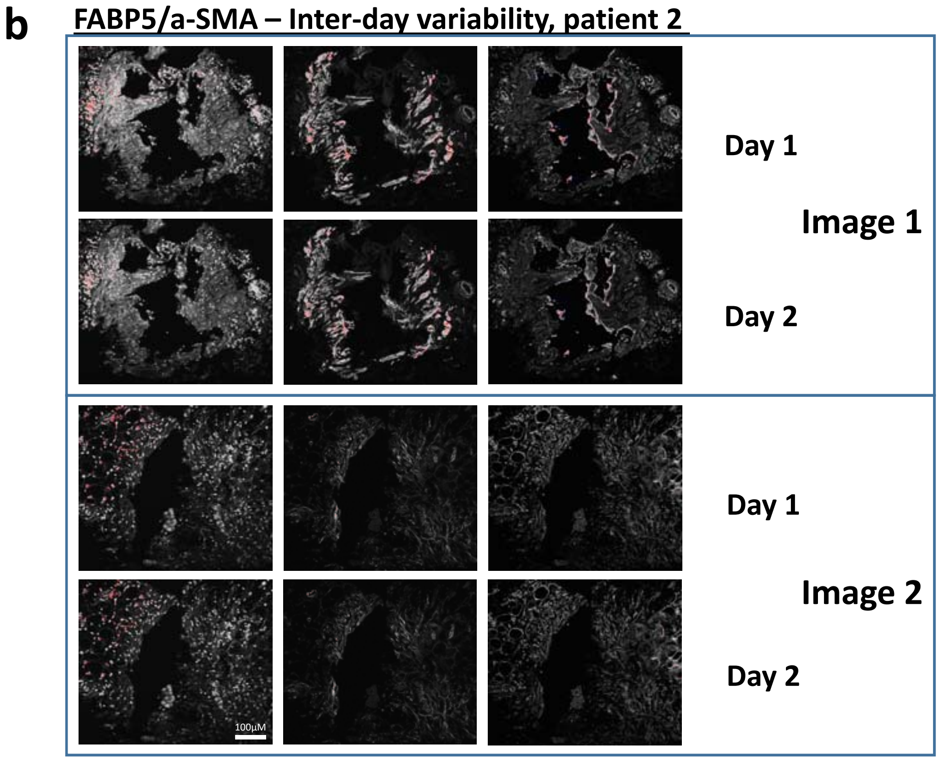

Figure 7: Inter-day calibration for staining and scoring.
a: Control sections were used to calibrate the gain and offset for stained tissues to allow inter-day comparison of staining intensity for CRABP2 and Cytokeratin (CK) showing at least 2 images for calibration.
b: Control sections were used to calibrate the gain and offset for stained tissues to allow inter-day comparison of staining intensity for FABP5 and αSMA showing at least 2 images for calibration. Scale bar: 100µm.

Supplementary Figure 8

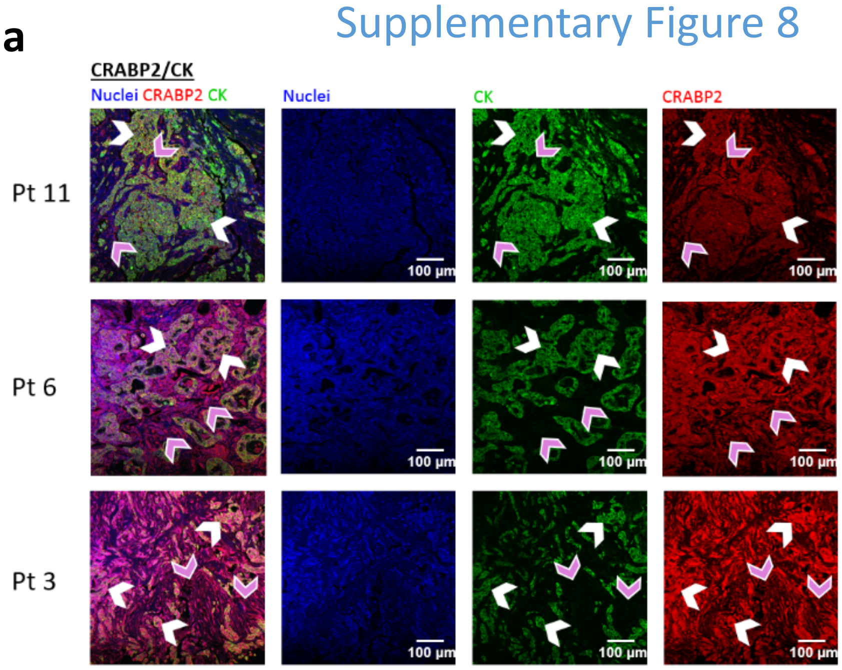

CRABP2 in CAF	CRABP2 in cancer
++	+
+++	++
+++	+++

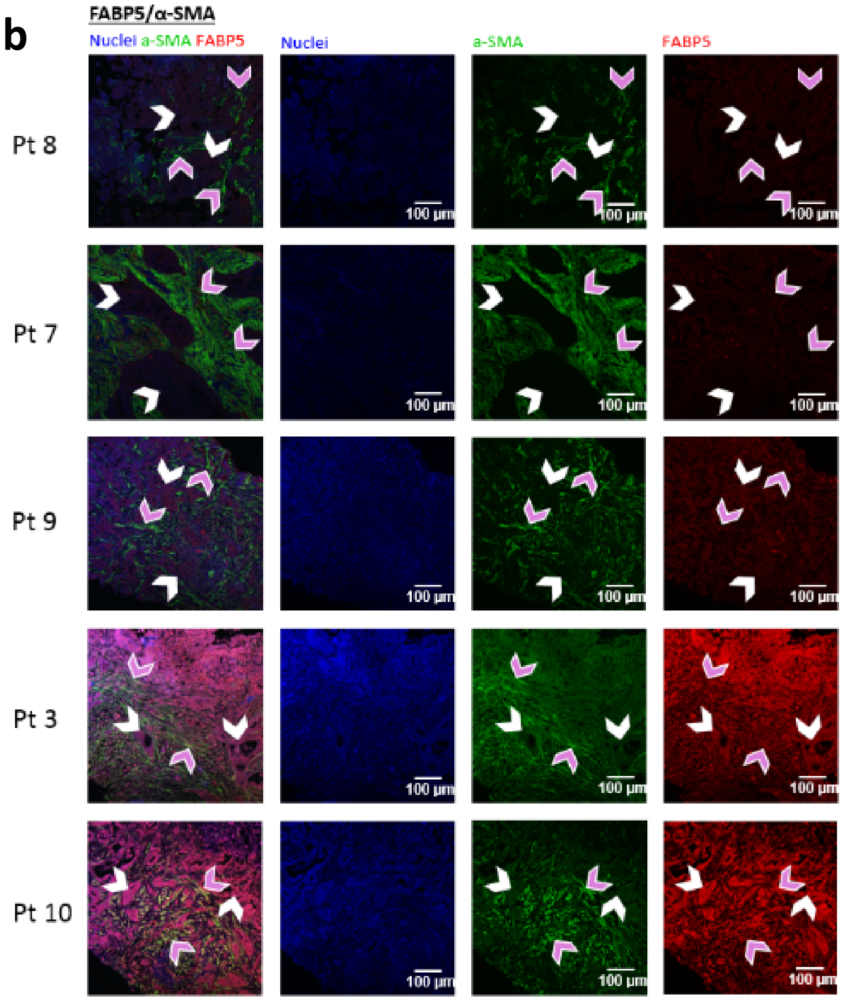

FABP5 in CAF	FABP5 in cancer
-	+/-
+/-	-
+	+
+++	++
+++	+++

Figure 8: Scoring for FABP5 and CRABP2 staining

a: Immuno-fluorescent staining demonstrating varying degrees of CRABP2 intensity, in cytokeratin (CK) stained (cancer) and unstained areas (stroma, based on nuclear morphology). White arrows indicate cancer cells, pink arrows indicate stromal cells. All settings were kept identical across all images taken. Semi-quantitative CRABP2 immunofluorescent intensity is displayed in table on right.

b: Immuno-fluorescent staining demonstrating varying degrees of FABP5 intensity, in α-SMA stained (CAF) and unstained areas (cancer, based on ductal morphology). White arrows indicate cancer cells; pink arrows indicate CAF (stellate) cells. All settings were kept identical across all images taken. Semi-quantitative FABP5 immuno-fluorescent intensity is displayed in table on right. Scale bar: 100µm

Supplementary Figure 9

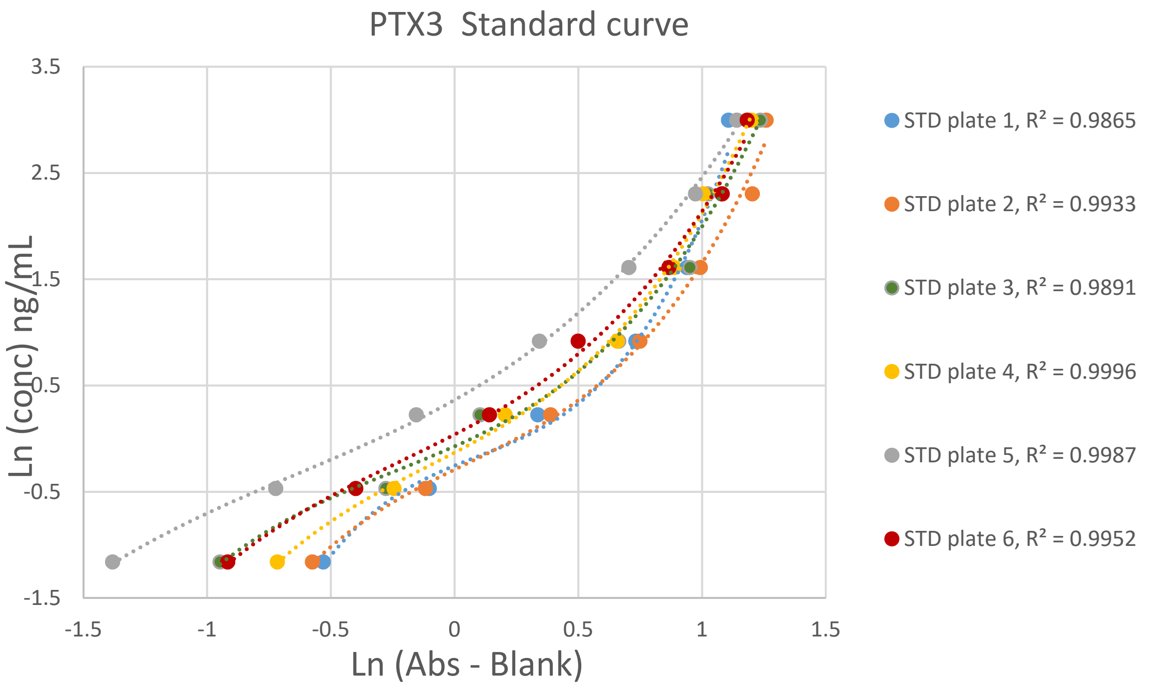

Figure 9: PTX3 assay calibration curves for standards.
Calibration curves for standards for plates on different days.

Phase I clinical trial repurposing all-trans retinoic acid (ATRA) as a stromal targeting agent for pancreatic cancer (STARPAC).

Kocher et al

Supplementary Tables

Supplementary Table 1

Dose level	Gemcitabine		Nab-Paclitaxel		ATRA	
DL1	80%	800 mg/m^2	80%	100 mg/m^2	33%	15 mg/m^2
DL2	80%	800 mg/m^2	80%	100 mg/m^2	66%	30 mg/m^2
DL3	80%	800 mg/m^2	80%	100 mg/m^2	100%	45 mg/m^2
DL4	100%	1000 mg/m^2	80%	100 mg/m^2	100%	45 mg/m^2
DL5	100%	1000 mg/m^2	100%	125 mg/m^2	100%	45 mg/m^2

Table 1: Dose Levels
Table shows dosing at various Dose Levels (DL) along with % of target Recommended Phase 2 Dose (RP2D) or Federal Drug Administration (FDA, USA) approved dosing for gemcitabine-nab-paclitaxel.

Supplementary Table 2

Characteristic	DL5 patients (N=19)	All Patients (N=27)
Age (years) Median (range)	65 (27 – 78)	66 (27 – 78)
Gender (M:F)	12:7	16:11
Race (White: Asian: Black: other)	13:3:0:3	20:3:1:3
Differentiation (well: moderate: poor: not defined)	0:6:6:7	0:9:7:11
T stage (Tx:T2:T3:T4)	1:2:3:13	2:2:6:17
N stage (Nx:N0:N1)	6:4:9	8:4:15
M stage (M0:M1)	3:16	4:23
ECOG status (0:1)	10:9	16:11

Table 2: Baseline patient features
Patient demographics and disease characteristics at baseline for all patients and those in Dose Level 5 (RP2D). ECOG: Eastern Co-operative Oncology Group. Male (M) and Female (F) patients

Supplementary Table 3

a

A. Adverse events (AEs) by system organ class (SOC): All Patients (N=27)						
**CTCAE v4.03 SOC term / Grade**	1	2	3	4	5	Total
**General disorders and administration site conditions**	8	11	7	0	0	26
**Gastrointestinal disorders**	9	10	7	0	0	26
**Nervous system disorders**	12	5	2	0	1	20
**Infections and infestations**	8	3	6	1	0	18
**Metabolism and nutrition disorders**	7	4	4	2	0	17
**Skin and subcutaneous tissue disorders**	7	10	0	0	0	17
**Blood and lymphatic system disorders**	1	8	4	2	0	15
**Respiratory, thoracic and mediastinal disorders**	10	2	1	1	0	14
**Investigations**	4	5	2	0	0	11
**Vascular disorders**	2	2	1	1	1	7
**Psychiatric disorders**	6	1	0	0	0	7
**Renal and urinary disorders**	3	1	0	0	0	4
**Musculoskeletal and connective tissue disorders**	4	0	0	0	0	4
**Injury, poisoning and procedural complications**	3	0	0	0	0	3
**Ear and labyrinth disorders**	0	1	0	0	0	1
**Cardiac disorders**	1	0	0	0	0	1

b

B. Adverse events (AEs) by system organ class (SOC): DL5 Patients (N= 19)						
**CTCAE v4.03 SOC term / Grade**	1	2	3	4	5	Total
**General disorders and administration site conditions**	5	7	6	0	0	18
**Gastrointestinal disorders**	7	9	2	0	0	18
**Nervous system disorders**	10	4	2	0	0	16
**Metabolism and nutrition disorders**	6	4	2	2	0	14
**Skin and subcutaneous tissue disorders**	4	8	0	0	0	12
**Blood and lymphatic system disorders**	1	6	3	1	0	11
**Infections and infestations**	7	2	2	0	0	11
**Respiratory, thoracic and mediastinal disorders**	6	2	1	1	0	10
**Investigations**	2	5	1	0	0	8
**Vascular disorders**	2	1	0	1	1	5
**Psychiatric disorders**	3	1	0	0	0	4
**Renal and urinary disorders**	3	1	0	0	0	4
**Musculoskeletal and connective tissue disorders**	4	0	0	0	0	4
**Injury, poisoning and procedural complications**	3	0	0	0	0	3

Table 3: Adverse events
Adverse events in the STARPAC clinical trial according to System Organ Class (SOC), where the worst toxicity for each SOC has been reported for

a. All patients (n=27)
b. Dose Level 5 patients (n=19)

Supplementary Table 4

	DL2	DL3	DL4	DL5	All
N (total)	3	2	3	19	27
N (evaluable)	3	0	2	15	20
Median PFS in months [a] (95% CI) [b]	NR (1.4-NR)	NA (NA)	7.4 (7.4-NR)	6.4 (3.5-NR)	7.4 (3.5-11.7)
Median OS in months (pre-specified, 95% CI) [b]	11.5 (8.4-NR)	NA (NA)	8.7 (8.7-NR)	10.9 (8.6-NR)	11.5 (8.6-NR)
Median OS in months (post-hoc, 95% CI) [b]	11.5 (8.4-NR)	NA (NA)	8.7 (8.7-NR)	11.7 (8.6-15.7)	11.5 (8.6-15.7)
ORR [c]	33%	NA	0%	27%	25%
DCR [d]	67%	NA	100%	60%	65%

Table 4: Secondary endpoints
Median PFS and OS, as well as RR and DCR for all patients and those on Dose Levels 2, 3, 4 and 5 on an evaluable population.
CI = Confidence interval. N/A = Not applicable. NR = Not reached.
N (total) = Number of patients in the safety set population for the specified group of patients.
N (Evaluable) = Number of patients in the evaluable population for the specified group of patients.
[a] RECIST version 1.1 progression is based on overall visit response, and thus not programmatically derived from Target, Non Target and New Lesions.
[b] Calculated using the Kaplan-Meier technique. Evaluable population analysis uses patient data up to 12 months from registration into trial and post-hoc analysis uses data beyond 12 months.
[c] Objective Response Rate (ORR): Response is defined as the number of patients who have measurable disease at baseline with at least one confirmed response of complete response (CR) or partial response (PR).
[d] Disease Control Rate (DCR): Disease control is defined as the number of patients with at least one confirmed response of CR or PR or maintained stable disease (SD) for at least 8 weeks.

Supplementary Table 5

Treatment compliance and exposure (Safety Set population)					
	DL2	DL3	DL4	DL5	All
	(N=3)	(N=2)	(N=3)	(N=19)	(N=27)
**Nab-Paclitaxel**					
**Received their full allocated dose over all cycles, n (%)**	0	1 (50)	0	4 (21)	5 (19)
**≥1 dose reduction over all cycles, n (%)**	1 (33)	0	2 (67)	10 (53)	13 (48)
**≥1 dose interruption over all cycles, n (%)**	0	0	0	1 (5)	1 (4)
**≥1 dose modification or interruption not in protocol over all cycles, n (%)**	0	0	0	0	0
**Total treatment duration (months), median (range)**	4.2 (1.4 – 5.6)	0.4 (0.3 – 0.5)	5.1 (1.0 – 5.3)	5.3 (0.03 – 11.5)	4.2 (0.03 – 11.5)
**Cumulative dose intensity (%) over the first 6 cycles, median (range)**	61 (33 – 75)	14 (11 – 17)	74 (17 – 86)	72 (6 – 100)	67 (6 – 100)
**Gemcitabine**					
**Received their full allocated dose over all cycles, n (%)**	0	1 (50)	0	7 (37)	8 (30)
**≥1 dose reduction over all cycles, n (%)**	1 (33)	0	1 (33)	6 (32)	8 (30)
**≥1 dose interruption over all cycles, n (%)**	0	0	0	0	0
**≥1 dose modification or interruption not in protocol over all cycles, n (%)**	0	0	0	0	0
**Total treatment duration (months), median (range)**	4.2 (1.4 – 5.6)	0.4 (0.3 – 0.5)	5.1 (1.0 – 5.3)	5.3 (0.03 – 11.5)	4.2 (0.03 – 11.5)
**Cumulative dose intensity (%) over the first 6 cycles, median (range)**	61 (33 – 81)	14 (11 – 17)	84 (17 – 94)	79 (6 – 100)	72 (6 – 100)
**ATRA**					
**Dispensed their full allocated dose over all cycles, n (%)**	2 (67)	2 (100)	2 (67)	17 (89)	23 (85)
**≥1 dose reduction over all cycles, n (%)**	0	0	1 (33)	2 (11)	3 (11)
**≥1 dose modification or interruption not in protocol over all cycles, n (%)**	0	0	0	0	0
**≥1 missed or returned dose over all cycles, n (%)**	3 (100)	0	3 (100)	10 (53)	16 (59)
**Total treatment duration (months), median (range)**	4.5 (1.4 – 5.6)	0.4 (0.3 – 0.5)	5.1 (1.1 – 5.5)	5.3 (0.2 – 6.2)	4.5 (0.2 – 6.2)
**Cumulative dose intensity (%) over the first 6 cycles, median (range)**	79 (34 – 94)	17 (16 – 17)	94 (28 – 98)	82 (16 – 105)	79 (16 – 105)

Table 5: Treatment compliance and exposure
Treatment compliance and exposure for gemcitabine, nab-paclitaxel and ATRA.
N = Number of patients in the Safety Set population for the specified group of patients.
Cumulative dose intensity over the first 6 cycles is the actual amount of study drug received over the first 6 cycles divided by the expected amount of study drug received over the first 6 cycles. The expected amount of study drug will be calculated based on the dose and schedule specified in the protocol. Total treatment duration (months) = (last dose date - first dose date +1) / (365.25/12). Note: ATRA is administered for 6 cycles whereas gemcitabine/nab-paclitaxel is administered until disease progression.

Supplementary Table 6

		DL2	DL3	DL4	DL5	DL3_4_5
	**n**	**3**	**2**	**3**	**18**	**23**
Cycle 1	**lin AUC**	484 (203-763)	559 (252-866)	246 (0-576)	326 (163-489)	340 (199-482)
	**Cmax**	220	198	121	119	126
	**Tmax**	3	2	3	2	2
Cycle 2	**lin AUC**	157 (32-282)	NA	202 (80-324)	402 (270-534)	378 (260-496)
	**Cmax**	102	NA	169	146	150
	**Tmax**	2	NA	2	2	2
Cycle 3	**lin AUC**	114	NA	234 (15-452)	352 (213-491)	339 (215-463)
	**Cmax**	78	NA	195	130	140
	**Tmax**	3	NA	2	2	2

Table 6: ATRA pharmacokinetics
Summary statistics on C_{max} (ng/ml), T_{max} (h) and Area Under Curve (linear AUC, h*ng/ml, 95% confidence interval) for each dose level.

Supplementary Table 7

a

Calibration Standard Level	Nominal ATRA concentration (ng/mL)	Mean measured ATRA concentration (ng/mL)	SD	%CV	%RE	n
**S1**	50	50.2	4.00	8.0	0.4	18[a]
**S1A**	62.5	62.9	2.2	3.5	0.6	6[b]
**S2**	100	99.5	5.8	5.8	-0.5	18[a]
**S2A**	125	121	6.8	5.6	-3.2	6[b]
**S3**	250	249	12.2	4.9	-0.6	24[c]
**S4**	500	513	16.2	3.2	2.6	24[c]
**S5**	1000	992	60.6	6.1	-0.8	24[c]
**S6**	2000	2013	80.4	4.0	0.6	24[c]
**S7**	4000	4022	112	2.8	0.5	24[c]

b

QC Level	Nominal ATRA concentration (ng/mL)	Mean measured ATRA concentration (ng/mL)	SD	%CV	%RE	n
**LOW**	100	102	8.3	8.1	2.0	24[a]
**MID 1**	300	295	16.6	5.6	-1.7	18[b]
**MID 2**	800	810	45.8	5.7	1.3	24[a]
**HIGH**	4000	4080	274	6.7	2.0	24[a]

c

Nominal ATRA		Calculated ATRA QC concentration (ng/mL)							
		Intra-day (n=6)*				Inter-day (n=18)**			
**QC Conc. (ng/mL)**		Mean	SD	%CV	%RE	Mean	SD	%CV	%RE
**LLOQ**	50	53.6	6.7	12.5	7.2	49.8	5.4	10.9	-0.4
**LOW**	100	99.8	6.7	6.7	-0.2	97.2	7.4	7.6	-2.8
**HIGH**	800	759	22.9	3.0	-5.1	780	41.8	5.4	-2.5
**HIGH**	4000	4130	112	2.7	3.3	4060	128	3.2	1.5

d

Stability test	Nominal ATRA concentration (ng/mL)	Calculated ATRA concentration (ng/mL) Mean	± SD	%CV	%RE
**Short term (24 h, RT)**	100	89.4	7.01	7.8	-10.6
	4000	3630	66.5	1.8	-9.3
**Freeze/thaw (-80˚C to RT)**	100	112	2.68	2.4	112
	4000	4070	57.1	1.4	102
**Long term (534 days*, -80˚C)**	100	97.7	3.30	3.4	97.7
	4000	3980	89.3	2.2	99.5

Table 7: Calibration and standardization of ATRA measurements.

a. Calibration curve precision and accuracy data summary for ATRA measurements by LC MS/MS. Samples were analyzed over 12 batches. Initial three batches analyzed with a slightly higher lower limit of quantification (LLOQ =62.5 ng/mL ATRA). Method had been fully validated using an LLOQ of 50 ng/mL. [a]n = 2 x 9, [b]n = 2 x 3, [c]n = 2 x 12

b. Inter-run precision and accuracy QC data summary (during clinical study). Samples were analyzed over 12 batches. An extra mid-level QC was introduced, when it was observed all the study sample concentrations were at the lower end of the calibration curve. [a]n = 2 x 12, [b]n = 2 x 9.

c. Intra-day and Inter-day precision and accuracy QC data summary (from validation study). *Intra-day statistics are presented from the 1st intra-day run only - intra-day criteria were met for all analytes in all three intra-day runs. *Inter-day n=18 (3 days x 6 replicates).

d. Stability of ATRA in human plasma (heparinized).

SD: standard deviation, reported to 3 significant figures; CV: Coefficient of variation; RE: Relative error.

Supplementary Table 8

a

Vitamin A ≥1.5µmol/L and ≤2.5µmol/L					
	DL2	DL3	DL4	DL5	All
	(N=3)	(N=2)	(N=3)	(N=19)	(N=27)
**Cycle 1**	1 / 3 (33)	1 / 2 (50)	2 / 2 (100)	7 / 18 (39)	11 / 25 (44)
**Cycle 2**	1 / 3 (33)	0 / 0	2 / 2 (100)	4 / 11 (36)	7 / 16 (44)
**Cycle 3**	1 / 2 (50)	0 / 0	0 / 2 (0)	2 / 11 (18)	3 / 15 (20)
**Cycle 4**	0 / 2 (0)	0 / 0	0 / 2 (0)	2 / 9 (22)	2 / 13 (15)
**Cycle 5**	0 / 0	0 / 0	1 / 2 (50)	3 / 8 (38)	4 / 10 (40)
**Cycle 6**	0 / 1 (0)	0 / 0	1 / 1 (100)	2 / 9 (22)	3 / 11 (27)

b

Vitamin A ≥1µmol/L and ≤2.5µmol/L					
	DL2	DL3	DL4	DL5	All
	(N=3)	(N=2)	(N=3)	(N=19)	(N=27)
**Cycle 1**	3 / 3 (100)	2 / 2 (100)	2 / 2 (100)	13 / 18 (72)	20 / 25 (80)
**Cycle 2**	2 / 3 (67)	0 / 0	2 / 2 (100)	9 / 11 (82)	13 / 16 (81)
**Cycle 3**	1 / 2 (50)	0 / 0	2 / 2 (100)	9 / 11 (82)	12 / 15 (80)
**Cycle 4**	2 / 2 (100)	0 / 0	1 / 2 (50)	6 / 9 (67)	9 / 13 (69)
**Cycle 5**	0 / 0	0 / 0	2 / 2 (100)	6 / 8 (75)	8 / 10 (80)
**Cycle 6**	0 / 1 (0)	0 / 0	1 / 1 (100)	6 / 9 (67)	7 / 11 (64)

Table 8: Optimal biological dose (OBD): Vitamin A levels (Safety Set population)
Results are presented as: Number of patients with Vitamin A at end of specified cycle / Number of patients with Vitamin A results at end of specified cycle (%). Patients who had ATRA dose reductions during a cycle were removed from the "Cycle" summaries for that dose reduction cycle and all subsequent cycles. N = Number of patients in the Safety Set population for the specified group of patients

a. 5µmol/L and ≤2.5µmol/L
b. ≥1µmol/L and ≤2.5µmol/L

Supplementary Table 9

Manufacturer	GE Healthcare, Waukesha, WI, USA	Philips Healthcare, Best, The Netherlands
Model	Discovery MR450	Achieva
Field strength / T	1.5	1.5
Receive coil(s)	HD BodyFull	SENSE XL torso coil
Patient position	Feet first supine	Head first supine
Sequence	Single-shot EPI	Single-shot EPI
Slice orientation	Axial	Axial
FOV (read) / mm	256	288
FOV (phase) / mm	256	288
Slice thickness / mm	5	5
Slices per station	30	30
Phase-encode direction	AP	AP
Acquired matrix (read)	128	128
Reconstructed matrix (read)	128	126
Acquired pixel size / mm x mm	3.0 x 3.0	3.0 x 3.0
Reconstructed pixel size / mm x mm	1.5 x 1.5	1.5 x 1.5
Echo time (TE) / ms	59	64
Repetition time (TR) / ms	3500	3500
Number of signal averages (NSA)	8	3
Parallel imaging	ASSET, reduction factor 2	SENSE, reduction factor 2
Partial Fourier	yes	no
Fat suppression	Water-selective excitation	SPAIR, delay time 110 ms
Diffusion gradient scheme	DSE	monopolar
Number of diffusion directions	3	3
Diffusion encoding scheme	ALL	Gradient overplus
Diffusion-weighted images	Trace	Trace
Acquired b-values / s mm^{-2}	0, 100, 400, 800	0, 100, 400, 800
Breathing instructions	Free breathing	Free breathing
Navigator / gating	none	none
Acquisition time	4 mins 39 s	4 mins 53 s

Table 9: Cross-platform protocols for DW-MRI.

Supplementary Table 10

Mean ± SD	Tumour Volume on DWI (cm^3)	D (10^{-3} mm^2/s)
Baseline	74.9 ± 42.7	1.35 ± 0.10
Follow Up (Day 22-28)	71.2 ± 42.1	1.52 ± 0.16
P value	0.19	0.01*
Median [LQ, UQ]<br>Δ (%)	-11.7<br>[-14.6, 5.4]	11.2<br>[9.5, 15.3]

Table 10: Changes in tumor volume and ADC as measured by DW-MRI
Wilcoxon matched-pairs signed rank test

Supplementary Table 11

Primary/Secondary	Antibody	Catalogue reference	IF dilution
	Rat FABP5	R&D Systems MAB3077	1:200
	Mouse CRABP2	Santa Cruz Biotechnology sc-159411	1:100
	Rabbit Cytokeratin	Dako Z0622	1:100
**Primary**	Mouse α-SMA	Sigma Aldrich F3777	1:500
	Rabbit IgG - 488	Thermofisher A11034	1:500
	Rat IgG - 546	Thermofisher A11081	1:500
**Secondary**	Mouse IgG - 546	Thermofisher A11030	1:500

Table 11: Antibodies used for immunoflourescent staining.

Phase I clinical trial repurposing all-trans retinoic acid (ATRA) as a stromal targeting agent for pancreatic cancer (STARPAC).

Kocher et al

Supplementary Note (Trial Protocol)

Barts Health NHS Trust

Protocol v2.0, dated 28 September 2015

TITLE OF THE PROTOCOL:

A Phase 1B study repurposing ATRA as stromal targeting agent along with gemcitabine and nab-Paclitaxel for pancreatic cancer (STAR_PAC)

EudraCT Number: 2015-002662-23
Sponsor Number: 010432QM
Ethics Committee Ref: 15/SC/0548

Sponsor:	Barts Health NHS Trust<br>Representative of Sponsor:<br>Dr Sally Burtles<br>Director of Research Services & Business Development<br>Joint Research Management Office (JRMO)<br>QM Innovation Building<br>5 Walden Street<br>London, E1 2EF, UK<br><br>Tel: +44(0)20 7882 7260<br>Fax: +44(0)20 7882 7276<br>Email: sponsorsrep@bartshealth.nhs.uk
Chief Investigator:	Dr David Propper<br>Barts Cancer Institute<br>Queen Mary University of London<br>Old Anatomy Building<br>Charterhouse Square<br>London, EC1M 6BQ, UK<br><br>Tel: +44(0)20 7882 8272<br>Fax: +44(0)20 7882 8409<br>Enquiries: d.j.propper@qmul.ac.uk
Trial Chairman & Co-Chief Investigator	Prof Hemant Kocher<br>Professor of Liver and Pancreas Surgery<br>Centre for Tumour Biology<br>Barts Cancer Institute<br>Queen Mary University of London<br>John Vane Science Centre<br>Charterhouse Square<br>London, EC1M 6BQ, UK.<br><br>Tel: +44(0)20 7882 3579<br>Fax: +44(0)20 7882 3884<br>Email: h.kocher@qmul.ac.uk

STUDY CONTACTS

Funders

Medical Research Council (Development Pathway Funding Scheme), 14th floor, One Kemble Street, London,C2B 4AN

CELGENE International II Sàrl, Rue des Moulins 4, 2108 Couvet, Switzerland.

Chief Clinical Collaborators

Dr Debashis Sarker, Kings College London; Dr Roopinder Gillmore, Royal Free Hospital NHS Foundation Trust; Dr Harpreet Wasan, Imperial Healthcare NHS Trust; Dr Fieke Froeling, Imperial College London; Prof Brian Davidson, Royal Free Hospital NHS Foundation Trust.

Study Statistician

Dr Bernard North, Senior Statistician, Wolfson Institute, Queen Mary University of London, Old Anatomy Building, Charterhouse Square, London, EC1M 6BQ, UK.

Tel: +44(0)20 7882 3494, Fax:+44(0)20 7882 3890, Email: b.v.north@qmul.ac.uk.

Pharmacodynamic and translational endpoint analysis

Prof Hemant Kocher, Barts Cancer Institute, Queen Mary University of London, Charterhouse Square, London, EC1M 6BQ, UK. Tel: +44(0)20 7882 3579; Fax: +44(0)20 7882 3884; Email: h.kocher@qmul.ac.uk

Pharmacokinetic Analysis

Prof Duncan Jodrell, CRUK Cambridge Institute, Li Ka Shing Centre, Robinson Way, Cambridge, CB2 0RE.

Tel: +44(0)1223 769750; F: +44(0)1223 769510; Email: duncan.jodrell@cruk.cam.ac.uk

Imaging sub-study

Prof Nandita DeSouza, Consultant Radiologist, Department of Diagnostic Radiology, The Royal Marsden Hospital, London. Tel: +44(0)20 8661 3289; Email: Nandita.Desouza@icr.ac.uk

ATRA specific considerations and independent opinion

Prof David Grimwade, Department of Medical and Molecular Genetics, 8th Floor, Tower Wing, Guy's Hospital, London SE1 9RT. Tel: +44(0)20 7188 3699. Email: david.grimwade@kcl.ac.uk

Tissue Banking

Barts Pancreas Tissue Bank, (REC Ref: 13/SC/0592, HTA Licensing number: 12199),Barts Cancer Institute, Queen Mary University of London, John Vane Science Centre, Charterhouse Square, London, EC1M 6BQ, UK. Tel: +44 (0)20 7882 3573 | Fax: +44 (0)20 7882 3884 | Email: a.ambily@qmul.ac.uk

Trial Coordination

STARPAC Clinical Trial Coordinator, Centre for Experimental Cancer Medicine, Barts Cancer Institute, Queen Mary University of London, Old Anatomy Building, Charterhouse Square, London, EC1M 6BQ, UK

Tel: +44(0)20 7882 8272, Fax: +44(0)207 882 8409, Email: bci-STARPAC@qmul.ac.uk

IMP Liaison

Cheryl Lawrence, Senior Research Pharmacist, Centre for Experimental Cancer Medicine, Barts Cancer Institute, Queen Mary University of London, Old Anatomy Building, Charterhouse Square, London, EC1M 6BQ, UK. Tel: +44(0)207 882 8492, Fax: +44(0)207 882 5958, Email: Cheryl.Lawrence@bartshealth.nhs.uk

Participating Sites

A list of participating sites can be obtained from the STARPAC Trial Coordinator.

PROTOCOL SIGNATURE PAGE

Signature of the Chief Investigator and Trial Chairman:

The clinical study as detailed within this research protocol (Version 2.0, Dated 28 September 2015), and any subsequent amendments, involves the use of an investigational medicinal product and will be conducted in accordance with the Research Governance Framework for Health & Social Care (2005), the World Medical Association Declaration of Helsinki (1996), Principles of ICH-GCP, and the current regulatory requirements, as detailed in the Medicines for Human Use (Clinical Trials) Regulations 2004 (UK S.I. 2004/1031) and any subsequent amendments of the clinical trial regulations.

Chief Investigator Name: Dr David Propper

Signature and Date: ______________________ __ __ / __ __ __ / __ __ __ __

Trial Chairman & Co-Chief Investigator Name: Prof Hemant Kocher

Signature and Date: ______________________ __ __ / __ __ __ / __ __ __ __

Signature of the Statistician:

The clinical study as detailed within this research protocol (Version 2.0, Dated 28 September 2015), or any subsequent amendments, involves the use of an investigational medicinal product and will be conducted in accordance with the Research Governance Framework for Health & Social Care (2005), the World Medical Association Declaration of Helsinki (1996), Principles of ICH E6-GCP, ICH E9 - Statistical principles for Clinical Trials, ICH E10 - Choice of Control Groups and the current regulatory requirements, as detailed in the Medicines for Human Use (Clinical Trials) Regulations 2004 (UK S.I. 2004/1031) and any subsequent amendments of the clinical trial regulations.

Statistician Name: Dr Bernard North

Signature and Date: ______________________ __ __ / __ __ __ / __ __ __ __

Signature of the Principal Investigator:

The clinical study as detailed within this research protocol (Version 2.0, Dated 28 September 2015), and any subsequent amendments, involves the use of an investigational medicinal product and will be conducted in accordance with the Research Governance Framework for Health & Social Care (2005), the World Medical Association Declaration of Helsinki (1996), Principles of ICH-GCP, and the current regulatory requirements, as detailed in the Medicines for Human Use (Clinical Trials) Regulations 2004 (UK S.I. 2004/1031) and any subsequent amendments of the clinical trial regulations.

Principal Investigator Name: ____

Principal Investigator Site: ____

Signature and Date: ______________________ __ __ / __ __ __ / __ __ __ __

TABLE OF CONTENTS

STUDY SYNOPSIS 9
1 INTRODUCTION 13
1.1 Background 13
1.2 Overview of ATRA 17
1.3 Pre-clinical work on ATRA as a stromal targeting agent in pancreatic cancer18
1.4 Rationale 20
1.5 Benefit/risk and ethical assessment 22
2 STUDY OBJECTIVES 23
2.1 Primary Objectives 23
2.2 Secondary Objectives 24
2.3 Tertiary/Exploratory Objectives 24
3 INVESTIGATIONAL PLAN 24
3.1 Overall design 24
3.2 Target Accrual 25
4 PATIENT SELECTION 26
4.1 Inclusion Criteria 26
4.2 Exclusion Criteria 27
5 STUDY PROCEDURES AND SCHEDULE OF ASSESSMENTS 28
5.1 Patient Identification 28
5.2 Informed consent procedure 28
5.3 Patient Registration 29
5.4 Withdrawal of Patients / Discontinuation of Treatment 29
5.5 Study Assessments 30
5.6 Exploratory Research 38
5.7 Follow-up, Survival and Anti-cancer therapy 41
6 INVESTIGATIONAL MEDICINAL PRODUCT 41
6.1 Definition of the IMP 41
6.2 Product Sourcing, Manufacture and Supply 41
6.3 Packaging and Labelling 41
6.4 Receipt of IMP Supplies 42
6.5 Drug Storage 42
6.6 Drug Accountability 42
6.7 Dispensing of IMP 43
6.8 Treatment regimens and dosing levels 43
6.9 Study Drug Administration 44
6.10 Return or Destruction of IMP 45
6.11 Concomitant Therapies 45

6.12 Dose Modification and Delay 47
7 PHARMACOVIGILANCE 52
7.1 Definition of an AE 52
7.2 Recording of Adverse Events 52
7.3 Severity of Adverse Events 52
7.4 Causality of Adverse Events 52
7.5 Abnormal Laboratory Test Results 52
7.6 Serious Adverse Event (SAE) or Serious Adverse Reaction (SAR) 53
7.7 Reporting of SAEs 53
7.8 Non-Reportable Events 53
7.9 Definition of an Adverse Reaction (AR) 54
7.10 Serious Adverse Reaction (SAR) 54
7.11 Suspected Unexpected Serious Adverse Reaction (SUSAR) 54
7.12 Reporting of SUSARs 54
7.13 Pregnancy 54
7.14 Annual Reporting 55
7.15 Urgent Safety Measures 55
8 STATISTICAL CONSIDERATIONS 56
8.1 Definition of study endpoints 56
8.2 Determination of sample size 56
8.3 Populations for Analyses 57
8.4 Endpoints 57
8.5 End of Study Definition 61
8.6 Statistical presentation 61
9 DATA HANDLING AND RECORD KEEPING 62
9.1 Confidentiality 62
9.2 Study Documents 62
9.3 Case Report Forms (CRFs) 62
9.4 Record Retention and Archiving 63
9.5 Compliance 63
10 STUDY MANAGEMENT 63
10.1 Trial Management Group (TMG) 63
10.2 Safety Review Committee 64
11 CLINICAL GOVERNANCE ISSUES 64
11.1 Ethical Considerations 64
11.2 Summary Monitoring Plan 64
11.3 Audit and Inspection 65
11.4 Reporting of Serious Breaches in GCP or the Trial Protocol 65
12 STUDY FINANCES 65

12.1 Funding Sources 65
12.2 Patient expenses / payments 65
13 SPONSORSHIP AND INDEMNITY 65
14 PUBLICATION POLICY 65
15 APPENDIX 1: RECIST CRITERIA (VERSION 1.1) 67
1.1 Introduction 67
1.2 Definition of measurable, non-measurable, target and non-target lesions 67
1.3 Methods of assessment 68
1.4 Tumour response evaluation 70
1.5 Specifications for radiological imaging 73
16 REFERENCES 76

LIST OF TABLES AND FIGURES IN THE TEXT

Tables

Table 1: Stromal targeting PDAC trials relevant to STARPAC(Feb 2015) 14
Table 2: Schedule of assessments. 31
Table 3: Additional assessments triggered by specific AEs 35
Table 4: Blood collection plan for PK profiling. 36
Table 5: Dose modification levels for ATRA and Gemcitabine and nab-paclitaxel 48
Table 6: Gemcitabine/Nab-Paclitacel dose modifications for Haematological Adverse Events (AE)s. 48
Table 7: Dose modification of Gemctiabine/Nab-Paclitaxel for Non-Haematological Adverse Events (AE)s as per SmPC. Please see table 5 for dose modification levels. 49
Table 8: Dose modifications of Gemcitabine/ Nab-Paclitaxel for Non-Haem AEs not listed on SmPC on Day 1 of a cycle 49
Table 9: Dose modifications of Gemcitabine/Nab-Paclitaxel for Non-Haem AEs not listed on SmPC within a Cycle 49
Table 10: Populations for analysis 57

GLOSSARY OF TERMS AND ABBREVIATIONS

9-cis RA	9-cis retinoic acid
13-cis RA	13-cis retinoic acid
AE	Adverse event
ALT	Alanine aminotransferase
ANC	Absolute neutrophil count
ALP	Alkaline phosphatase
APR	Annual Progress Report
AST	Aspartate aminotransferase
ATRA	All trans-retinoic acid
BSA	Body surface area
cCR	Clinical complete response
CECM	Centre for Experimental Cancer Medicine (Coordinating Centre)
CI	Chief Investigator
cPR	Clinical partial response
CRF	Case Report Form
CT	Computed tomography
CTA	Clinical trials authorisation
CTCAE	(National Cancer Institute) Common Toxicity Criteria For Adverse Events
CXR	Chest X-Ray
DNA	Deoxyribonucleic acid
DSUR	Development Safety Update Report
DW_MRI	Diffusion weighted magnetic resonance imaging
ECG	Electrocardiogram
ECOG	Eastern Cooperative Oncology Group
EU	European Union
FBC	Full Blood Count
FISH	Fluorescence in situ hybridization
GCP	Good clinical practice
GLP	Good laboratory practice
HTA	Human tissue authority
IB	Investigator's Brochure
ICF	Informed Consent Form
ICH	International conference on harmonisation
IHC	Immunohistochemistry
IMP	Investigational medicinal product
ISF	Investigator Site File
IV	Intravenous
LDH	Lactate dehydrogenase
LFTs	Liver function tests
LLN	Lower limit of normal
MHRA	Medicines & healthcare products regulatory agency
MRI	Magnetic Resonance Imaging
MTD	Maximum tolerated dose
NCI	National Cancer Institute
NCRI	National Cancer Research Institute
NCRN	National Cancer Research Network
NIHR	National Institute for Health Research
PA	Posteroranterior
PCR	Polymerase chain reaction
PD	Progressive Disease
PI	Principal Investigator
PIS	Patient Information Sheet

PR	Partial Response
PS	Performance Status
PSF	Pharmacy Site File
RBC	Red blood cell count
REC	Research Ethics Committee
RNA	Ribonucleic Acids
SAE	Serious adverse event
SAR	Serious adverse reaction
SD	Stable disease
SDV	Source data verification
SPARC	Secreted protein acidic and rich in cysteine
SPC	Summary of Product Characteristics
SRC	Safety Review Committee
SUSAR	Suspected unexpected serious adverse reaction
TMA	Tissue micro array
TMF	Trial Master File
TMG	Trial management group
TSC	Trial steering committee
ULN	Upper limit of normal
WBC	White blood cell count

STUDY SYNOPSIS

**Title**	A Phase 1B study repurposing ATRA as stromal targeting agent along with gemcitabine and nab-Paclitaxel for pancreatic cancer (STAR_PAC)
**Main Objectives**	**Primary Objectives**<br>*Part 1:* To determine the Maximum Tolerated Dose (MTD) of the combination of Gemcitabine, nab-Paclitaxel and ATRA<br>*Part 2:* To determine the optimal biological dose (OBD) of ATRA when given in combination with Gemcitabine and nab/paclitaxel<br><br>**Secondary Objectives**<br>**Part 1**<br>• To investigate the PK of ATRA when given in combination with Gemcitabine and nab-Paclitaxel.<br>• To assess the change in serum Vitamin A levels relative to baseline upon various dosing levels of ATRA.<br>• To assess the safety and tolerability of ATRA when given in combination with Gemcitabine and nab-Paclitaxel.<br>• To assess the efficacy of ATRA when given in combination with Gemcitabine and nab-Paclitaxel based on objective response rate (ORR).<br>• To assess the efficacy of ATRA when given in combination with Gemcitabine and nab-Paclitaxel based on disease control rate (DCR).<br>• To assess the efficacy of ATRA when given in combination with Gemcitabine and nab-Paclitaxel based on progression free survival (PFS).<br>• To assess the efficacy of ATRA when given in combination with Gemcitabine and nab-Paclitaxel based on overall survival (OS).<br><br>**Part 2**<br>• To assess the change in serum Vitamin A levels relative to baseline upon various dosing levels of ATRA.<br>• To assess the safety and tolerability of ATRA when given in combination with Gemcitabine and nab-Paclitaxel.<br>• To assess the efficacy of ATRA when given in combination with Gemcitabine and nab-Paclitaxel based on objective response rate (ORR).<br>• To assess the efficacy of ATRA when given in combination with Gemcitabine and nab-Paclitaxel based on progression-free survival (PFS).<br>• To assess the efficacy of ATRA when given in combination with Gemcitabine and nab-Paclitaxel based on disease control rate (DCR).<br>• To assess the efficacy of ATRA when given in combination with Gemcitabine and nab-Paclitaxel based on overall survival (OS).<br><br>**Tertiary/Exploratory Objectives**<br>• To explore potential serum derived biomarkers that may help predict response to ATRA in combination with Gemcitabine and nab-Paclitaxel

	• To explore potential tumour derived biomarkers that may help predict response to ATRA in combination with Gemcitabine and nab-Paclitaxel<br>• To explore potential imaging biomarkers that may help predict response to ATRA in combination with Gemcitabine and nab-Paclitaxel
**Phase**	1B
**Number of Patients**	The maximum number of patients to be included in Part 1 of the trial is 24. Up to 6 patients may be recruited into each level.<br>The maximum number of patients to be included in Part 2 of the trial is 10.
**Inclusion Criteria**	1. Written informed consent prior to admission to this study<br>2. Age ≥18 years. No upper age limit.<br>3. WHO performance status 0 or 1<br>4. Life expectancy ≥12 weeks<br>5. Histologically proven Pancreatic ductal adenocarcinoma (PDAC). Formalin fixed, paraffin embedded tumour sample from the primary cancer must be available for central testing. If not available or sufficient patients will be asked to undergo an US or CT guided biopsy prior to study entry to satisfy this eligibility criterion.<br>6. Locally advanced or metastatic disease which is measurable according to the Response Evaluation Criteria in Solid Tumours (RECIST v1.1)<br>7. Received no prior systemic therapy for metastatic or locally advanced disease. Prior adjuvant chemotherapy (with Gemcitabine or any other drug/s) is allowed if completed at least 6 months previously.<br>8. Adequate hematologic and end organ function, defined by the following laboratory results obtained within 14 days prior to the first study treatment:<br>a. Absolute Neutrophil Count ≥ 1.5 x 10^9/l (without granulocyte colony-stimulating factor support within 2 weeks prior to the first study treatment)<br>b. Platelet count ≥ 100 x 10^9/l (without transfusion within 2 weeks prior to the first study treatment)<br>c. Haemoglobin ≥ 10 g/dl (transfusion permitted to establish target haemoglobin levels prior to the first study treatment)<br>d. Calculated creatinine clearance (e.g. Cockcroft-Gault) ≥ 50 ml/min<br>e. Bilirubin level ≤ 1.5 ULN (patients with known Gilbert disease who have bilirubin levels ≤ 3 x ULN may be enrolled). Patients must be able to undergo biliary stenting if required before or, if required, during the trial<br>f. AST or ALT <2.5 x ULN or <5 x ULN in the presence of liver metastases<br>g. Alkaline phosphatase (ALP) <2.5 x ULN or <5 x ULN in the presence of liver and/or bone metastases<br>h. INR and aPTT ≤1.5 x ULN; this applies only to patients who are not receiving therapeutic anticoagulation; patients receiving therapeutic anticoagulation should be on a stable dose.<br>9. Female patients of child-bearing potential are eligible, provided they have a negative serum or urine pregnancy test within 7 days prior to the first dose of study treatment, preferably as close to the first dose as possible. All patients with reproductive potential must agree to use a medically acceptable method

	of contraception throughout the treatment period and for 1 month after discontinuation of ATRA and /or Gemcitabine/nab-Paclitaxel (whichever is the latest) and for 6 months after discontinuation for male patients. Acceptable methods of contraception include IUD, oral contraceptive, sub-dermal implant and double barrier (condom with a contraceptive sponge or contraceptive pessary). Micro-dosed progesterone preparations ("mini-pill") are an inadequate method of contraception during treatment with ATRA. If patients are taking this pill they should be instructed to stop and another form of contraceptive should be prescribed instead.<br>10. Able to follow protocol requirements as assessed by the Principal Investigator.
**Exclusion Criteria**	1. Patient has known brain metastases.<br>2. Patient has experienced a significant reduction in performance status between the screening/ baseline visit and within 72 hours prior to commencement of treatment as per trial protocol, as per the Investigator's assessment<br>3. Patients with pre-existing sensory neuropathy >grade 1<br>4. History of malignancy in the last 5 years; with the exception of:<br>a. Patients with prior history of in situ cancer or basal or squamous cell skin cancer are eligible.<br>b. Patients with other malignancies are eligible if they were cured by surgery alone or surgery plus radiotherapy and have been continuously disease-free for at least 5 years.<br>5. Patient has active, uncontrolled bacterial, viral, or fungal infection(s) requiring systemic therapy.<br>6. Patient has HIV, or active hepatitis B or C infection.<br>7. Patient has undergone major surgery, other than diagnostic surgery (i.e., surgery done to obtain a biopsy for diagnosis without removal of an organ), within 4 weeks prior to Day 1 of treatment in this study.<br>8. Patient has a history of allergy (including soya bean or peanut allergies) or hypersensitivity to any of the study drugs or any of their excipients, or the patient exhibits any of the events outlined in the Contraindications or Special Warnings and Precautions sections of the products or comparator SmPC or Prescribing Information.<br>9. History of connective tissue disorders (e.g., lupus, scleroderma, arteritis nodosa).<br>10. Patient with a history of interstitial lung disease, history of slowly progressive dyspnoea and unproductive cough, sarcoidosis, silicosis, idiopathic pulmonary fibrosis, pulmonary hypersensitivity pneumonitis or multiple allergies.<br>11. Patient with high cardiovascular risk, including, but not limited to, recent coronary stenting or myocardial infarction in the past year.<br>12. History of Peripheral Artery Disease (e.g., claudication, Leo-Buerger's disease).<br>13. Patient has serious medical risk factors involving any of the major organ systems, or serious psychiatric disorders, which could compromise the patient's safety or the study data integrity.

	14. Concurrent treatment with other experimental drugs or participation in another clinical trial with any investigational drug ≤30 days prior to study entry depending on the half-life of the investigational drug and/or guidance issued by the IMP manufacturer. Please contact the STARPAC Coordinating team for further information.<br>15. Patient is taking any prohibited concurrent medication, including Vitamin A supplements and is unwilling to stop use prior to and during the trial. Refer to section 6.11.2.<br>16. Patient is pregnant, planning to become pregnant or breast feeding.<br>17. Patient has received a live vaccine within four weeks prior to receiving their first dose of study treatment.<br>18. Patient is unwilling or unable to comply with study procedures, as assessed by the Principal Investigator.

1 INTRODUCTION

1.1 Background

1.1.1 Pancreatic cancer

Pancreatic cancer (PDAC) is the fourth-highest cancer killer worldwide (~310,000 patients), responsible for 6% of cancer deaths (overall median survival ~3 months). With an ageing population, the Pancreatic Cancer Action Network anticipates this age-related disease becoming the second leading cause of cancer death by 2020.[1] The only potentially curative procedure, surgical excision, is feasible in a minority of patients (~20% resectable pancreatic ductal adenocarcinoma, rPDAC, or borderline resectable, brPDAC), but even of these patients the majority (~80%) die within 5 years.[2] Most patients present with advanced disease, with approximately 35% of patients having locally advanced (laPDAC), involving major vessels that precludes surgical excision, and approximately 45% are diagnosed with metastatic disease (mPDAC). Treatment options for these patients remain minimally effective with only recently the additional option of FOLFIRINOX (5-fluorouracil/leucovorin/irinotecan/oxaliplatin) or Nab-Paclitaxel/Gemcitabine giving an additional survival benefit of 4 and 2 months, respectively, when compared to Gemcitabine monotherapy.[3, 4] However, despite encouraging results, these regimens are associated with significant toxicities and the outlook for patients with pancreatic cancer remains poor. Thus, there is an urgent need for better therapeutic strategies. This early phase clinical trial will develop a neo-adjuvant regimen, to inform a phase II/ III clinical trial focusing on laPDAC and brPDAC, to enable surgical resection.

1.1.2 Stromal targeting in pancreatic cancer

One of the characteristics of pancreatic cancer is its intense desmoplastic stroma, which can account for up to 70% of the tumour volume and actively participates in tumour initation, progression, metastases and the response to therapy. Various studies have investigated the effects of agents targeting specific components of the stroma, including extracellular matrix components (enzymatic depletion of hyaluronic acid by a PEGylated human recombinant PH20 hyaluronidase [5, 6]), altered immune response (CXCL12 inhibition, CD40 agonists or blocking granulocyte-macrophage colony-stimulating factor[7-11]) or targeting of activated pancreatic stellate cells (hedgehog or connective tissue growth factor (CTGF) inhibition [12, 13]). Other stromal directed therapies target the characteristic hypovascular, hypoxic microenvironment with for example the hypoxia-activated prodrug TH-302, gamma secretase inhibitors or low dose of Cilengitide and Verapamil as a vascular promotion therapy.[14-16]

Although an obvious target in pancreatic cancer, the role of the tumour microenvironment in cancer progression and therapy response is dichotomous with some elements having a more tumour suppressive role.[17, 18] In pancreatic cancer, pancreatic stellate cells (PSCs) change from a quiescent, vitamin A storing phenotype to an activated myofibroblast like cell, which drives the formation of the dense desmoplastic stroma.[19] Normalising the tumour stroma by reprogramming PSCs to their quiescent phenotype by the VDR ligand calcipotriol [20] or all-trans retinoic acid (ATRA) [9, 21], and thereby restoring a more physiological secretome, is an attractive approach and one that will be explored with this trial.

ATRA will be used in combination with nab-paclitaxel, which has additional effects on the tumour stroma. In patient-derived xenografts, combination treatment of nab-paclitaxel and gemcitabine resulted in a less dense desmoplastic stroma with increased vascularity and a 2.8 fold increase in the intratumour concentration of gemcitabine.[22] These findings were confirmed in compound Elas-tTA/tetO-Cre;KRAS$^{+/LSLG12Vgeo}$;TP53$^{flox/flox}$

mice and in a clinical study investigating the clinical and biological effects of gemcitabine and nab-paclitaxel as neoadjuvant therapy in patients with rPDAC or brPDAC. Pre-operative EUS elastography, tumours demonstrated less stiffness, after treatment with gemcitabine and nab-paclitaxel and resected surgical specimens showed a disrupted tumour stroma with decreased type I collagen expression and less cancer associated fibroblasts, compared to samples of patients treated with neoadjuvant chemoradiotherapy or no neoadjuvant therapy.[23] However tumours from Pdx1-Cre;LSL-KRAS^{G12D};LSL-TP53^{R172H} mice, which also demonstrate a significant response to nab-paclitaxel, there was no change in the tumour stroma and its effects were attributed to a decrease in the gemcitabine metabolizing enzyme cytidine deaminase via paclitaxel-mediated prodution of reactive oxygen species, resulting in increased intratumoural gemcitabine levels.[24]

1.1.3 Summary of current international clinical trials focusing on pancreatic cancer stroma

Multiple approaches targeting the tumour microenvironment have been studied. Table 1 lists these that as of February 2015 are most relevant to the current study (i.e. targeting of the tumour stroma and stromal targets in combination with gemcitabine and nab-paclitaxel). As of February 2015, the only trials that target the tumour stroma in the UK involve immune therapy with agents that block immune checkpoints using the monoclonal antibodies to cytotoxic T-lymphocyte associated protein-4 (CTLA-4; Ipilimumab), and either the programmed cell death 1 receptor (PD-1; Nivolumab) or its ligand, programmed cell death ligand 1 (PD-L1; MEDI4736). However both trials require patients to have progressed, not tolerated or refused standard first-line treatment.

Table 1: Stromal targeting PDAC trials relevant to STARPAC(Feb 2015)

Study ID	Title	Disease group	Phase	Stromal target	Recruitment
**UK trials targeting stroma**					
UKCRN 15824	CheckMate032: Nivolumab + Ipilimumab in solid tumours	mPDAC	I / II	Immune system (PD-1 and CTLA-4)	Recruiting, UK, multicentre
UKCRN 15886	MEDI4736 in advanced solid tumours	mPDAC	I / II	Immune system (PD-L1)	Recruiting, internationally
UKCRN 15437	TG01+ GM-CSF + Gem	rPDAC	I / II	Immune system	Suspended
**Non-UK tials targeting stoma in combination with Gemcitabine (Gem) and Nab-Paclitaxel**					
NCT01803282	GS-5745 +/- chemotherapy for advanced solid tumours	mPDAC	I	MMP-9	Recruiting, US
NCT01431794	Gem + Nab-Paclitaxel + LDE-225	brPDAC	I / II	Hedgehog signalling	Recruiting, US
NCT02030860	Gem + Nab-Paclitaxel +/- Paricalcitol	rPDAC	I / II	Vit D receptor signalling	Recruiting, US
NCT01839487	Gem + Nab-Paclitaxel +/- PEGPH20	mPDAC	II	Hyaluronan	Recruiting, US
NCT02210559	Gem + Nab-paclitaxel +/- FG-3019	laPDAC	II	CTGF	Recruiting, US
NCT01088815	Gem + Nab-Paclitaxel + GDC-0449	mPDAC	II	Hedgehog signalling	Recruiting, US
**Non-UK imaging trials**					
NCT01995240	Reproducibility and comparison of in vivo imaging (DCE-MRI, T2* MRI and DWI) and ex vivo immunohistochemical measurements of tumour vascularity, hypoxia and stroma	rPDAC			Recruiting, single centre, Netherlands
NCT01989000	Predictive value of pre-treatment DWI, DCE-MRI, T2*, and 18F-HX4-PET/CT on overall survival	rPDAC			Recruiting, single centre, Netherlands
NCT01715142	NEOPAX-001: Effect on Tumour Perfusion of Gem + Nab-Paclitaxel				In set up, internationally

In the United States, the study of the 1,25-dihydroxyergocalciferol analogue Paricalcitrol (NCT02030860) is of particular interest given the pre-clinical data of the vitamin D receptor (VDR) ligand calcipotriol, which was found to trans-differentiate activated PSCs back into a quiescent phenotype resulting in a reduction in collagen fibres and tumour volume when combined with Gemcitabine.[20] The rationale for the use and effects of calcipotriol is similar to these of ATRA; normalisation of the tumour stroma and restoration of homeostasis. In the single centre, randomised pilot study in the US, patients with rPDAC will receive one cycle of Gemcitabine with Nab-paclitaxel with or without Paricalcitol prior to surgery.

In addition to the interventional studies, assessment of the stromal response via dynamic imaging may serve as a predictive and/ or a pharmacodynamic biomarker and various studies are evaluating these in more depth.

1.1.4 Gemcitabine and nab-Paclitaxel combination in pancreatic cancer

Nab-paclitaxel is an albumin-bound formulation of paclitaxel that has demonstrated increased response rates and better tolerability when compared to solvent-based formulations. The advantages of nab-paclitaxel have mainly been attributed to

- a better pharmacokinetic (PK) profile with a greater fraction of unbound paclitaxel,
- a higher uptake due to the advantage of the normal transport properties of albumin such as receptor-mediated transcytosis across endothelial cells,
- the enhanced permeation and retention (EPR) effect,
- sequestering of nab-paclitaxel by secreted protein acidic and rich in cysteine (SPARC), an albumin-binding matricellular glycoprotein which is overexpressed in various tumour types, resulting in an increase in intratumour drug concentration,[25]
- prodution of reactive oxygen species, resulting inhibition of cytidine deaminase which reduces Gemcitabine catabolism and thereby increases Gemcitabine potency [24].

Pancreatic cancer is known to overexpress SPARC, which formed the basis for a phase I-II study of nab-paclitaxel with gemcitabine in patients with metastatic PDAC.[22] Dose limiting toxicities were sepsis and neutropenia and the maximum tolerated dose was established at 1,000 mg/m^2 of gemcitabine and 125 mg/m^2 of nab-paclitaxel on days 1, 8 and 15 of a 28 day cycle. With this regimen the overall response rate (ORR) was 48% with a median progression free survival (PFS) of 7.9 months and overall survival (OS) 12.2 months. Stromal SPARC expression was proposed as a potential biomarker of response as OS in patients with high SPARC expression was 17.8 months versus 8.1 months for those with low SPARC expression.[22]

In the subsequent randomized phase III trial (MPACT), 861 metastatic PDAC patients were randomized to either receive nab-paclitaxel/gemcitabine (nab-paclitaxel at 125mg/m^2 immediately followed by gemcitabine at 1000 mg/m^2 on days 1, 8, 15 of a 28 day cycle; 431 patients) or gemcitabine alone (430 patients). Combination treatment resulted in an increase in median OS of 1.8 months (8.5 months versus 6.7 months, Hazard Ratio 0.72, p<0.001), with superior survival gain maintained over 2 years. Most common grade 3/4 toxicities included myelosuppression, with an overall (all cycles included) incidence of grade 3/4 neutropenia of 38% in the combination arm versus 27% in the gemcitabine group, fatigue (17% versus 7%) and peripheral neuropathy (17% versus 1%). The risk of grade ≥3 neuropathy increased over time but was reversible, with resolution of symptoms one month after interrupting treatment.[4] SPARC expression however was not associated with OS or predictive of response to treatment (Hidalgo et al, Annals of Oncology: 2014; 25 (2): ii105–ii117 abstract).

On balance, the nab-paclitaxel/gemcitabine regimen appears to be better tolerated than FOLFIRINOX combination therapy. Since its approval by the US Food and Drug Administration (FDA) and Cancer Drug Fund (CDF) in the UK it now often is the regimen of choice as first-line therapy for patients with laPDAC or mPDAC and serves as a backbone regimen in multiple clinical trials. Based on the response seen in mPDAC patients in the MPACT trial, subsequent studies are investigating whether similar responses can be seen in rPDAC (APACT trial), brPDAC (PRICKLE) or laPDAC (LAPACT trial). The APACT trial aimed to study the effects of nab-paclitaxel/gemcitabine as adjuvant therapy with a primary outcome disease free survival. The Pancreatic Resectability in Cancers With Known Limited Extension (PRICKLE) is a phase 2a, single arm study

of nab-paclitaxel and gemcitabine in the neoadjuvant setting with the aim to assess both safety and efficacy to downstage tumours to allow resection after up to 6 cycles of chemotherapy. The LAPACT trial will investigate the effects of nab-paclitaxel/gemcitabine in laPDAC, in which patients will receive 6 cycles of nab-paclitaxel/gemcitabine after which they will either proceed to surgery, chemoradiotherapy or continue with nab-paclitaxel/gemcitabine with time to treatment failure as the primary endpoint.

Table 2 lists the studies, as of February 2015, using gemcitabine and nab-paclitaxel in the UK. The Systemic Therapy and Chemoradiation in Advanced Localised Pancreatic Cancer (SCALOP-II) trial follows on from a previous randomized phase 2 trial comparing capecitabine-based consolidation chemoradiotherapy (CRT) to gemcitabine-based CRT in patients with laPDAC (SCALOP-I). Although, in SCALOP-I the difference in primary endpoint (9 months PFS) was non-significant, the preferred regimen might be capecitabine-based consolidation CRT, as increased toxicity was seen with gemcitabine with no clinical outcomes benefit.[26] The SCALOP-II trial aims to study the effect of radiotherapy dose escalation, the addition of the HIV protease inhibitor Nelfinavir and nab-paclitaxel/gemcitabine in the treatment of laPDAC, patients will first receive 3 cycles of induction chemotherapy with nab-paclitaxel and gemcitabine before randomization to either continue with nab-paclitaxel and gemcitabine or start capecitabine-based CRT at 50.4Gy or 60Gy, with or without Nelfinavir.

Although the combination of gemcitabine with nab-paclitaxel results in a better tumour response, the effects on the stroma (as described in 1.1.2) and the mechanisms of their synergy are not fully understood. Based on preclinical data describing different mechanisms of action (stromal depletion with decrease in tumour stiffness versus inhibition of cytidine deaminase (CDA), a key enzyme for Gemcitabine inactivation, which was most significant after several days of nab-paclitaxel), the Scheduling nab-paclitaxel with Gemcitabine (SIEGE) trial will compare two different schedules of nab-paclitaxel and gemcitabine as first line treatment for patients with mPDAC: concomitant (as in the MPACT trial) versus sequential treatment with nab-paclitaxel followed by gemcitabine.

Table 2: Current trials of relevance using gemcitabine-nab-Paclitaxel backbone to study other agents (Feb 2015)

Study name	Phase	Patient group	Intervention	Estimated enrolment	Study ID
APACT	III	rPDAC	nab-paclitaxel/gem vs gemcitabine as adjuvant therapy	800 (multi-centre)	NCT01964430
PRICKLE	IIa	brPDAC	nab-paclitaxel/gem as neoadjuvant therapy	17 (single centre)	NCT02124369
SCALOP-II	II/III	laPDAC	induction chemotherapy with nab-paclitaxel/gemcitabine (3x) followed by either 3x nab-paclitaxel/gem, CRT at high or standard dose +/-Nelfinavir	289 (multi-centre)	NCT02024009
SIEGE	II	mPDAC	concomitant nab-paclitaxel/gemcitabine vs sequential nab-paclitaxel/gemcitabine	120 (multi-centre)	

In addition to the listed trials, a multi-arm umbrella study, PRIMUS (Pancreatic canceR Individualised Multi-arm Umbrella Study), has been proposed and is envisaged to open in 2015 as part of a UK-wide initiative (Precision-Panc). Biomaterial will be molecularly characterized and incorporated into pre-clinical work with next generation model systems with the ultimate aim to define the molecular landscape of pancreatic cancer and identify clinically relevant biomarkers, novel therapies and mechanisms of resistance. Based on the molecular profile or, in the event of insufficient evidence based on an unselected or enrichment strategy, patients will be allocated to individual therapeutic intervention arms in PRIMUS. The current study may benefit from this platform, facilitating genetic/molecular characterization and biomarker development for further clinical development.

1.2 Overview of ATRA

1.2.1 Metabolism

Retinoic acid (RA) is derived from Vitamin A (retinol). It is a non-peptidic, lipophilic molecule. It is readily available in the precursor forms from diet (beta-carotene, retinol) but retinol cannot be synthesised *de novo* in the human body. The metabolism of RA is affected by synthesis using retinol (RDH5, 10) and retinaldehyde dehydrogenases (RALDH1,2,3) along with contribution from alcohol dehydrogenases (ADH1,5,7)) as well as degradation by cytochrome p450 (CYP26: various). This is summarised elsewhere in review articles [27-30].

1.2.2 Clinical studies of using retinoic acid for cancer (main focus on APML)

ATRA alone or in combination with arsenic tri-oxide and /or induction chemotherapy has been established as standard treatment for patients with acute promyelocyctic leukemia through a number of phase I trials studying the pharmacokinetics [31-48] as well as phase II/III clinical trials, many of the pivotal ones being carried out in the UK under the MRC AML banner [47, 48].

The current recommended dose for ATRA is 45 mg/m^2 for adults and children for this disease given in two equally divided doses and rounded to the nearest 10 mg increment. It is given in an induction schedule to complete remission (maximum of 60 days) and consolidation/maintenance schedule (two weeks on alternating with two weeks off for a maximum of seven cycles) aiming for cure.

1.2.3 Clinical studies of combining retinoic acid analogues with other agents in pancreatic cancer

There are, as of February 2015, five clinical studies combining retinoic acid analogues with other agents. Three trials are exclusive to pancreatic cancer patients and further two trials are aimed at all solid tumours. All trials used 13-cis Retinoic acid.

Brembeck et al reported a phase II pilot trial of combining 13-cis RA with interferon-alpha (IFN-α) [49]. Histologically confirmed, unresectable pancreatic adenocarcinoma (Stage III (5 patients) or IV (17 patients)) received 1 mg/kg 13-cis RA orally and 6 million IU IFN-α subcutaneously daily. There were no patients with complete remission, 1 patient with partial remission (PR) (4.5%), and 14 patients (63.6%) demonstrated stable disease with a median duration of 5.0 months (range, 2.3-17.7+ months). Toxicity was mainly hematologic (13.6% WHO Grade 3: IFN-α) and non-hematologic toxicities did not exceed Grade 2 (skin and oral mucosa: related to 13-cis RA). The median OS was 7.7 months (range, 0.9-23.9+ months). The research group advocated further investigation in a phase III study, which has not taken place or has not been reported.

In contrast, Moore *et al* using the same combination, did not see any objective responses in six evaluable patients, to conclude that further exploration was not warranted for pancreatic adenocarcinoma [50]. Of note, toxicities were mild and reversible and a single patient had grade 3 fatigue.

In another phase II trial, 13-cisRA (1 mg/kg on days 1-14) was combined with Gemcitabine (1000 mg/m^2 on days 8, 15, 22) for a maximum of six cycles for 30 patients (median age 65 (44-79) years, median Karnofsky performance status 80% (range 60-100%)) laPDAC and mPDAC of which 20 were evaluable [51]. At a median follow-up of 21 months, there was 1 patient with partial remission, 7 patients with disease and 12 patients developed progressive disease. Toxicity was mainly haematological (8 grade 3 and 4 grade 4 neutropenia,

thrombocytopenia and anaemia). The median survival was 7.8 months (range 2.6-21.6 months). The combination of gemcitabine and 13-cis-RA was, thus, well tolerated but without significant improvement in RR or OS.

Two further trials included pancreatic cancer patients alongside other solid tumours. In a phase I trial, 19 patients with advanced solid tumours were treated with entinostat (start at 4 mg/m^2, and escalate at 1 mg/m^2) orally once weekly and with 13-cisRA orally twice daily (1mg/kg) x 3 weeks every 4 weeks. No objective responses were observed, however, prolonged stable disease occurred in patients with prostate, pancreatic, and kidney cancer [52].

54 patients with locally advanced, inoperable, or incompletely resected pancreatic and biliary tree adenocarcinomas were given 3 courses of cisplatin-gemcitabine induction chemotherapy. 38 progression-free patients were given consolidation radiotherapy with concurrent capecitabine of which 14 received immunotherapy: interleukin 2 and 13-cisRA (0.5 mg/kg) [53]. Median PF and OS for all 54 patients were 6.8 and 12.1 months, respectively. Patients treated with immunotherapy had a median PFS of 16.2 months, whereas median OS had not been reached after a median follow-up of 27.5 months. Grades 3 and 4 haematological and gastrointestinal toxicities were seen in 30% and 37% of all patients, respectively.

1.3 Pre-clinical work on ATRA as a stromal targeting agent in pancreatic cancer

1.3.1 Background of pancreas and retinoic acid

In a healthy pancreas, pancreatic stellate cells (PSC) store vitamin A or retinoic acid (RA). When activated, in cancer or inflammation, they lose their retinol stores and assume an activated myofibroblast phenotype. Activated PSC are the key cells responsible for the desmoplastic reaction [19, 21], a distinctive feature of pancreatic ductal adenocarcinoma (PDAC). Patients with PDAC lack biliary and pancreatic secretions, leading to deficiency of fat-soluble vitamins. Vitamin K deficiency is manifest and treated clinically. The lack of vitamin A is not clinically recognised in patients with PDAC [54, 55]. It is relevant to the patho-biology of PDAC as it may perpetuate PSC activation. RA is a vital signalling molecule regulating signalling pathways guiding embryonic pancreas development [56]. These embryonic signalling pathways are hijacked during pancreatic carcinogenesis [57].

1.3.2 ATRA as a therapeutic stromal targeting agent in PDAC (published data)

Based on this clinical and scientific rationale, we demonstrated, using organotypic cultures and a well-validated transgenic mouse model, that upon restoring vitamin A (All trans-retinoic acid: ATRA) depots within the PSC we could limit the desmoplastic reaction, and hinder cancer growth [21]. Experiments in *in vitro* and *in vivo* models and examination of human PDAC samples demonstrated that quiescent PSC are able to increase apoptosis, restrict proliferation and invasion/metastasis of cancer cells due to alteration of a multitude of signalling cascades utilised in the cross-talk between cancer cells and PSC.

Furthermore, we demonstrated that the activated PSC may hinder the migration of immune cells such as CD8+ T-cells, Natural Killer and B-cells into the immediate PDAC microenvironment [9]. This may explain the failure of immune therapy, such as the recent TELOVAC trial [58]. Treatment of transgenic mice with ATRA restores the immune infiltrate near the tumour cells thus enabling restoration of effective anti-tumour immunity [9].

1.3.3 ATRA as a therapeutic stromal targeting agent in PDAC (unpublished data)

Activated PSC may function like a fortress around tumour cells. Restoring quiescence in PSC using ATRA enables alteration of the tumour stroma in a multitude of ways [59]. Unpublished work also suggests that this reversal of PSC patho-biology causing stromal collapse improves the vascularity of the otherwise hypo-vascular PDAC, and, potentially increases the delivery of chemotherapeutic agents, such as Gemcitabine, to maximise cancer cell death and necrosis. Such combination therapy reduces tumour volume significantly whilst achieving therapeutic tissue and plasma levels for retinoic acid.

Modulating a single signalling cascade in isolation (eg., IPI-926 trial) is unlikely to be efficacious due to the inherent redundancy associated with signalling cascades [12, 18]. We believe ATRA is an agent which can normalise the desmoplastic stroma, influencing a multitude of signalling pathways between cancer-stellate, immune-stellate and endothelial-stellate cells creating far reaching implications for tumour behaviour, metastasis, angiogenesis, immune cell infiltrate. Thus, ATRA is an ideal agent to dampen amplified embryonic context-specific signalling cascades such as retinoic acid receptor (RAR), Wnt, IL-8, Hedgehog, FGF (Fibroblast Growth Factor), CXCL12 (chemokine C-X-C motif ligand) pathways amongst others [9, 21, 59-61] hijacked by cancer. This approach is thus distinct from utilising an agent to annull a single signalling cascade or the genetic approaches to target desmoplasia [17, 18].

1.3.4 Conclusions from studies on ATRA as a therapeutic stromal targeting agent in PDAC

These pre-clinical observations are of clinical relevance since All-Trans Retinoic Acid (ATRA), but not 9-cis- or 13-cis-retinoic acid is effective in reducing PSC proliferation by a G1 cell-cycle arrest and accumulation of lipid droplets, thus restoring their physiological role in the normal pancreas. The specificity of retinoid (RAR) and rexinoid (RXR) receptor isoforms, distinctly used and regulated by various RA, is vital in pancreatic embryogenesis and PSC biology [62]. Data from Professor Kocher's laboratory, Queen Mary University of London suggest a specific up-regulation of RARβ isoform by ATRA. This is pertinent, since 13-cisRA, has previously been found to be safe, albeit not effective, in PDAC patients in combination with either Gemcitabine [51], or Interferon [49]. This aspect is relevant to this phase I re-purposing study for ATRA, since only ATRA is relevant to PSC physiology and embryonic development of pancreas. ATRA has never been tested in PDAC clinical trials. This, therefore, provides sound clinical rationale and pre-clinical data to conduct innovative phase I/II trials as advocated by European and USA consensus reports [63, 64].

The recent advent of two new chemotherapy regimens: FOLFIRINOX [3] and Gemcitabine-nab-Paclitaxel [4, 22, 65], have demonstrated superior efficacy in OS over the existing Gemcitabine mono-therapy (NIHCE TA25, 2001[66]) for advanced PDAC. FOLFIRINOX is toxic and requires a number of dose modifications to enable other drugs / agents to be added on to the regimen. Consequently, Gemcitabine-nab-Paclitaxel has rapidly been adopted as the new backbone regimen for clinical trials (Table 1). Furthermore, separation of advanced PDAC to laPDAC and mPDAC has allowed development of targeted approaches. Whilst the dose-finding part of STARPAC includes all advanced PDAC, a subsequent phase II/III trial will restrict cohort to predictive biomarker, as identified by this phase I proposal, defined laPDAC. Our aim for a future phase II/III trial will be to downstage to enable curative surgical resection.

1.3.5 Clinical information of combining retinoic acid with gemcitabine or retinoic acid with paclitaxel in other tumours

Adding ATRA (20mg/m^2) to Paclitaxel in patients with Stage IIIB/IV non-small cell lung cancer increased response rate and progression free survival over those receiving Paclitaxel alone (with palcebo) with an acceptable toxicity profile warranting investigation in a Phase III trial [45]. In another pilot trial with breast

cancer patients combination of ATRA (45mg/m^2) to Paclitaxel improved response rates with acceptable toxicity [46].

In patients with AML above the age of 60 years, combination of cytosine arabinoside (another nucleoside analogue) and ATRA (45mg/m^2) demonstrated efficacy without additional toxicity [67].

1.4 Rationale

1.4.1 Scientific Rationale

- Unpublished work suggests that the reversal of PSC patho-biology leading to stromal collapse improves the vascularity of the otherwise hypo-vascular PDAC, and, potentially increases the delivery of chemotherapeutic agents, to maximise cancer cell death and necrosis.
- Pre-clinical obvservations have demonstrated that All-Trans Retinoic Acid (ATRA) is effective in reducing PSC proliferation by a G1 cell-cycle arrest, accumulation of lipid droplets, and a specific up-regulation of RARβ isoform.
- Pre-clinical work carried out within Professor Kocher's laboratory, Queen Mary University of London has for the first time shown that ATRA when combined with standard chemotherapy restores the physiological quiescent state of activated pancreatic stellate cells [68]. This facilitates changes in tumour cell characteristics such as reduced proliferation, increased apoptosis and necrosis, increased infiltrate of immune cells and alteration in tumour vasculature accompanied by collapse of the fibrotic stroma which together results in a substantial reduction of tumour size.
- This study therefore hypothesises that treatment with ATRA, at a dose achievable in humans, will alleviate Vitamin A deficiency in PDAC patients thus re-instating the pancreatic stellate cell physiology. This tissue level of ATRA has no direct effect on tumour cells, since stellate cells are the only known cellular stores for Vitamin A in the human body.

1.4.2 Combining ATRA with chemotherapy agents has deterimental effect on tumour growth.Rationale for Selection of Study Drugs and Schedules

The anticipated full (100%) dose, based on existing well-established schedules, of

- Gemcitabine is 1000mg/m2 i.v. on days 1,8,15 of a 28 day cycle (UK NIHCE guidelines TA25, [66]).
- nab-Paclitaxel is 125mg/m2 i.v. on days 1,8,15 of a 28 day cycle [4, 22].
- ATRA is 45 mg/m2 orally (in two divided doses) from days 1 to 15 of each cycle (UK MRC AML 15 trial [47, 48]).

However, in STARPAC the starting doses are below the recommended/licensed dose because:

- Phase 1 studies are typically designed to start at below recommended doses in order to minimise risks in treatments which have previously had limited or no testing in man. STARPAC also uses this rationalle as this combination of treatments has not previously been tested in man.Examples of lower doses of nab-Paclitaxel in phase I trials are: NCT02047500, NCT01934634, NCT01804530.
- Step-wise escalation in Bayesian Continuous Re-assessment Method (CRM) model would mean rapid enrolment and escalation to further dosing levels as compared to 3+3 design.
- In-depth study of DLTs for Gemcitabine-nab-Paclitaxel as well as ATRA were made based on published literature to give an estimate of accumulated toxicity as summarised below. Overlapping

toxicity is not anticipated by combining ATRA to the already accepted Gemcitabine-nab-paclitaxel regimen.

Further, Gemcitabine-nab-Paclitaxel was 80% of recommended/licensed dose because

1. Phase I/II Gemcitabine-nab-Paclitaxel [22] data suggested:
   - Two of six patients at dose level 1 (80% (100 mg/m2) nab-Paclitaxel dose, 100% Gemcitabine dose) had presumed DLT. This necessitated protocol modification to allow a total of 20 patients at dose level 1 rather than considering this dose level as having exceeded the MTD.
   - Across all nab-paclitaxel doses, patients received 81% of the planned dose and 85% of the planned gemcitabine dose.
   - Twenty-five percent of patients had a nab-paclitaxel dose reduction, with 20% in the 125mg/m2 cohort.
   - Thirty-one percent of patients had a gemcitabine dose reduction, with 43% in the 125 mg/m2 cohort.
   - For all patients and in the 125mg/m2 cohort, 72% and 70% of patients had a nab-paclitaxel dose delayed, respectively, mainly due to AEs.

2. Phase III Gemcitabine-nab-Paclitaxel [4] data suggested:
   - In the nab-paclitaxel–gemcitabine group, 41% of the patients had reductions in the nab-paclitaxel dose and 47% had reductions in the gemcitabine dose.
   - In total, 71% of all nab-paclitaxel doses administered during the study were at the full dose of 125 mg /m2.
   - The median relative dose intensity (the proportion of the administered cumulative dose relative to the planned cumulative dose) in the nab-paclitaxel–gemcitabine group was 81% for nab-paclitaxel and 75% for gemcitabine

1.4.3 Justification of selecting the target population

The efficacy of Gemcitabine alone is proven in mPDAC [66] as well as laPDAC (though it has not been separately studied). The efficacy of Gemcitabine-nab-Paclitaxel in mPDAC has been established in one Phase III trial [4]. A number of combination regimens are being tested in phase I/II with Gemcitabine-nab-Paclitaxel in laPDAC and mPDAC.

From the pre-clinical data (published [21] and unpublished [68]) it appears that the efficacy would be best demonstrated in laPDAC/brPDAC in a neo-adjuvant setting to test the hypothesis that stromal- and cancer-cell co-targeting can achieve tumour shrinkage to enable surgical resection.

However, as the frequency of brPDAC/laPDAC is low and there are other trials such as SCALOPII and ESPAC5F which may become available for these sub-groups, STARPAC intends to recruit patients with mPDAC as well as laPDAC.

1.4.4 Rationale for translational research and exploratory objectives

- Molecular analysis on archival or obtained tumour tissue will be performed based on earlier studies carried out by Professor Kocher's laboratory, Queen Mary University of London (e.g. PTX3, β-catenin, sFRP4, RARβ, FGF2, TGFβ) [9, 21, 60] or other reports (e.g. FABP5 and CRABP2 expression) [69] as possible pharmacodynamic (PD) and predictive biomarkers of response to ATRA.
- Normal surrogate tissue in the form of hair follicles and buccal smears, will be collected for additional PD studies. The rationale for the collection of hair follicles and buccal epithelial cells is based on their

relative ease of access, the demonstrated advantages for biomarker studies in phase I trials [70, 71] and the known importance of retinoic acid signalling in the development and maintenance of hair follicles and oral mucosa.[72-74] Several molecular markers will be tested in hair sheath cells and buccal epithelial cells and, if possible compared with data from tumour tissue.

- Serum biomarkers will be studied based on previous research carried out by Professor Kocher's laboratory, Queen Mary University of London (e.g. PTX3, sFRP4, CXCL12). In addition, peripheral blood mononuclear cells (PBMCs), in which expression levels of proteins of interest (e.g. RARβ) can be measured, have proven to be a valuable means to study the PD effects over time.[70, 71]

1.5 Benefit/risk and ethical assessment

1.5.1 Potential benefits

Based on the pre-clinical data [68], a slower tumour progression is anticipated as well as reduction in size of these tumours translating into prolonged survival without increased toxicity.

1.5.2 Potential risks

The risks and side-effects of all three drugs are well-established and summarised in section 4.8 of the current versions of their respective SmPCs. Gemcitabine in combination with nab-Paclitaxel is a licenced combination for the first-line treatment of adult patients with metastatic adenocarcinoma of the pancreas.

ATRA (also known as Tretinoin) is routinely dispensed from NHS pharmacies for the treatment of Acute Myeloid Leukemia. The side effects of ATRA are well documented through routine use. The STARPAC trial will use ATRA in combination with a licensed chemotherapy combination (Gemcitabine and nab-Paclitaxel) for the first time. The exact side effect profile of this combination of treaments has not yet been established. Therefore, as with the development of any new treatment regimen, we will evaluate if there is alteration in the toxicity profile as a result of this combination.

Based on *in silico* analysis, the risks of excess toxicity are minimal and safety plan has been put in place to protect the participants.

With the proposed Dose Level 1 we are unlikely to give 'sub-optimal' dose of chemotherapy agents Gemcitabine and nab-Paclitaxel. Therefore there are no risks from sub-optimal dosing of main backbone chemotherapy.

1.5.3 Safety Plan

Entry:

Eligibility criteria for this study were selected to enhance the safety of patients in this trial. A number of exclusion criteria are specifically based on the known safety profiles of the study drug treatments (see below), including nonclinical and clinical data for Gemcitabine, nab-Paclitaxel, ATRA. The complete list and description of eligibility criteria for this study are provided in section 4 of the protocol.

Within Trial:

There appear to be no overlapping toxicities for the three drugs. Guidelines for the management of the known toxicities are provided in the study protocol. All enrolled patients will be evaluated clinically and with standard

laboratory tests before and at regular intervals during their participation in this study. Safety evaluations will consist of medical interviews, recording of AEs, physical examinations, and laboratory measurements. Patients will be evaluated for AEs (all grades), SAEs, and any AEs requiring drug interruption or discontinuation throughout the course of the study. Any outcomes of these pre-specified early safety reviews that affect study conduct will be communicated in a timely manner to the investigators, the Ethics Committee (EC) and Competent Authority (CA) as required.

Dosing levels

Step-wise dose levels have been built into the trial to reduce the potential toxicities. The probability of frequency of toxicities at each dose level are summarised as:

**Anticipated dose levels**	**Gemcitabine & nab-Paclitaxel**		**ATRA**		**All three drugs (anticipated additive)**
**D1**	10%	Projected	0%	projected	10%
**D2**	10%	Projected	5%	projected	15%
**D3**	10%	Projected	10%	Various studies*	20%
**D4**	15%	**[22] [4]**	10%	Various studies*	25%
**D5**	20%		10%	Various studies*	30%

**Various studies* [31-48]

Main DLTs for Gem-nab-Paclitaxel are neutropenia (~20%), peripheral neuropathy (17%), fatigue (17%) and diarrohea (6%). [4, 22]. Whilst pnueomnitis is not common, it has been highlighted as a possible DLT for nab-Paclitaxel in the SIEGE trial (written communication from the SIEGE CI to all participating sites, dated 25 February 2015)

Main DLTs for ATRA are muco-cutaneous (5%) and triglyceridemia (10%). No correlation with PK or PD was observed. ***Various studies*

Please refer to sections 8.2 and 8.4 for further detail.

Dose reductions

Gemcitabine-nab-Paclitaxel had been well studied in a phase III trial with recommended dose reductions as described in SmPC. Dose modifications for neutropenia and/or thrombocytopenia and other adverse reactions at the start of a cycle or within a cycle for patients with pancreatic adenocarcinoma are well specified in the SmPC and section 6.3.

2 STUDY OBJECTIVES

2.1 Primary Objectives

- **Part 1:** To determine the Maximum Tolerated Dose (MTD) of the combination of Gemcitabine, nab-Paclitaxel and ATRA
- **Part 2:** To determine the optimal biological dose (OBD) of ATRA when given in combination with Gemcitabine and nab/paclitaxel

2.2 Secondary Objectives

Part 1

- To investigate the PK of ATRA when given in combination with Gemcitabine and nab-Paclitaxel.
- To assess the change in serum Vitamin A levels relative to baseline upon various dosing levels of ATRA.
- To assess the safety and tolerability of ATRA when given in combination with Gemcitabine and nab-Paclitaxel.
- To assess the efficacy of ATRA when given in combination with Gemcitabine and nab-Paclitaxel based on objective response rate (ORR).
- To assess the efficacy of ATRA when given in combination with Gemcitabine and nab-Paclitaxel based on disease control rate (DCR).
- To assess the efficacy of ATRA when given in combination with Gemcitabine and nab-Paclitaxel based on progression free survival (PFS).
- To assess the efficacy of ATRA when given in combination with Gemcitabine and nab-Paclitaxel based on overall survival (OS).

Part 2

- To assess the change in serum Vitamin A levels relative to baseline upon various dosing levels of ATRA.
- To assess the safety and tolerability of ATRA when given in combination with Gemcitabine and nab-Paclitaxel.
- To assess the efficacy of ATRA when given in combination with Gemcitabine and nab-Paclitaxel based on objective response rate (ORR).
- To assess the efficacy of ATRA when given in combination with Gemcitabine and nab-Paclitaxel based on progression-free survival (PFS).
- To assess the efficacy of ATRA when given in combination with Gemcitabine and nab-Paclitaxel based on disease control rate (DCR).
- To assess the efficacy of ATRA when given in combination with Gemcitabine and nab-Paclitaxel based on overall survival (OS).

2.3 Tertiary/Exploratory Objectives

- To explore potential serum derived biomarkers that may help predict response to ATRA in combination with Gemcitabine and nab-Paclitaxel
- To explore potential tumour derived biomarkers that may help predict response to ATRA in combination with Gemcitabine and nab-Paclitaxel
- To explore potential imaging biomarkers that may help predict response to ATRA in combination with Gemcitabine and nab-Paclitaxel

3 INVESTIGATIONAL PLAN

3.1 Overall design

This is an open-label, multicentre phase 1B study of ATRA administered with Gemcitabine and nab-Paclitaxel. There are two parts to this study:

Part 1

- A dose-escalation part in untreated advanced pancreatic cancer patients to determine the MTD. Treatment doses will be assigned at registration. Dose escalation will be carried out according to emerging DLTs. At the end of part 1, a recommended part 2 dose will be defined based on the MTD and taken forward to part 2. Please refer to sections 8.2 and 8.4 for further details on dose escalation.
- Part 2 A dose expansion part to determine the OBD. Patients will start treatment at the dose selected during part 1 of the study and doses may be adjusted based on the results of the serum Vitamin A levels measured at the end of each treatment Cycle.

Assessments

AE, PK studies, RECIST evaluations and survival analysis as well as research tumour/normal surrogate tissue/blood samples and DW_MRI (optional) will be carried out prospectively.

Duration of treatment

In both parts of the study patients will receive ATRA, Gemcitabine and nab-Paclitaxel in 28 day cycles. ATRA will be administered for 6 cycles whereas Gemcitabine/nab-Paclitaxel will be administered until disease progression. Treatment may be discontinued earlier due to unacceptable toxicities or death or because the patient requests to be withdrawn from study treatment. If treatment with Gemcitabine/nab-Paclitaxel is stopped prior to the patient completing 6 cycles of treatment with ATRA, the patient may continue on treatment with ATRA alone until the 6 cycles are completed, at the discretion of the treating physician.

Follow Up

On completion of study treatment patients will attend a safety visit 30 days (±7days) after the last dose of Gemcitabine/nab-paclitaxel. Patients will subsequently enter a Survival Follow-up Period. During this time data will be collected every three months regarding further anti-cancer therapy, disease status and survival status. A mimimum of 12 months follow-up from the date of registration is required for all surviving patients. As the terminal half life of ATRA is short, 45 minutes, a safety visit has not been incorporated in the study design for patients who discontinue Gemcitabine/nab-paclitaxel but remain on ATRA treatment.

3.2 Target Accrual

The number of patients to be enrolled in part 1 of the study (dose escalation part) will be dependent on the continuous reassessment of DLTs. A maximum of 24 patients will be enrolled in this part of the study.

A maximum of 10 patients will be enrolled into part 2 of the study.

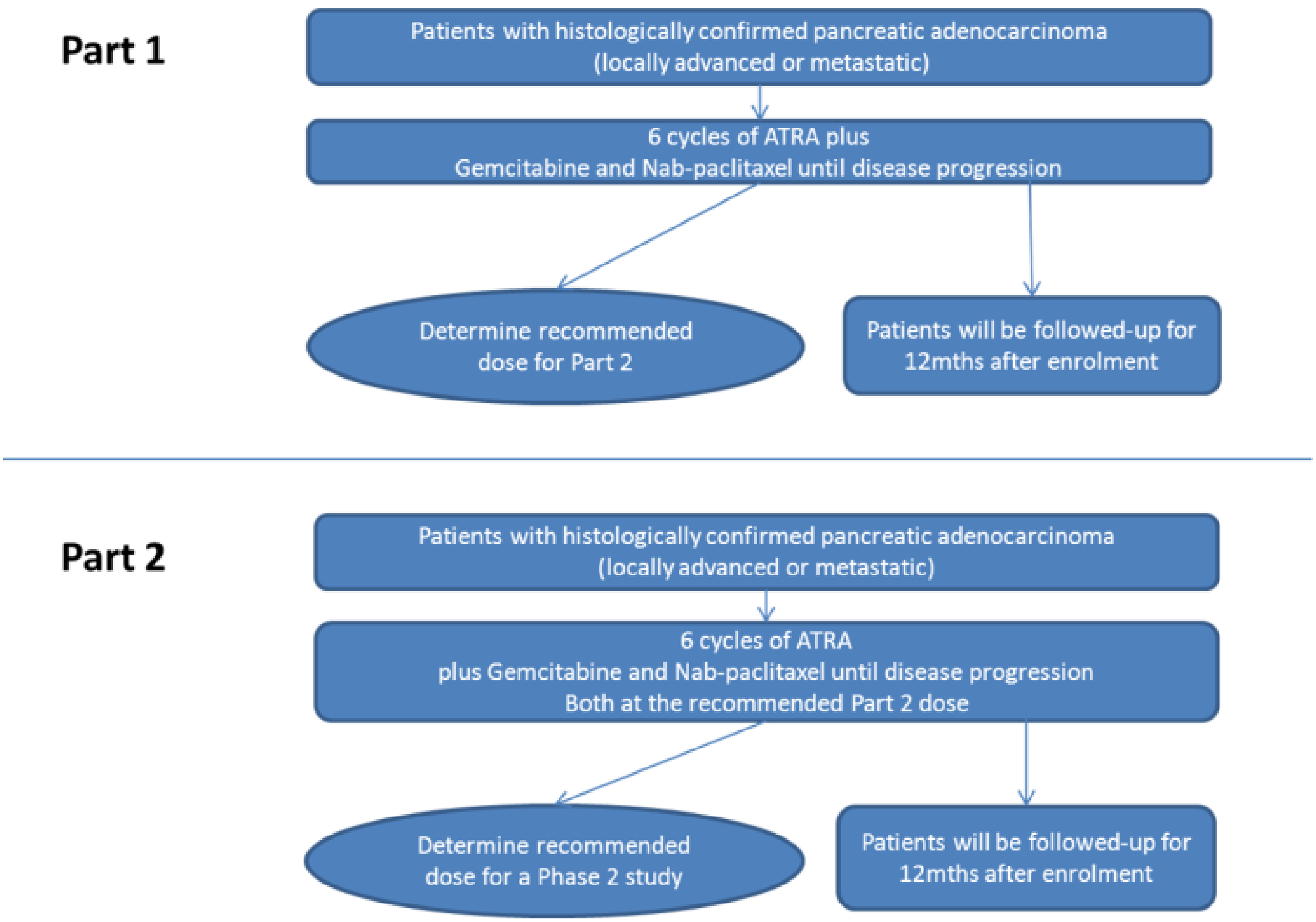

Figure 1: Overall Study Trial Schema (Phase 1B).

4 PATIENT SELECTION

4.1 Inclusion Criteria

Each patient must meet **all of the following inclusion criteria** to be enrolled in the study:

1. Written informed consent prior to admission to this study
2. Age ≥18 years. No upper age limit.
3. WHO performance status 0 or 1
4. Life expectancy ≥12 weeks
5. Histologically proven Pancreatic ductal adenocarcinoma (PDAC). Formalin fixed, paraffin embedded tumour sample from the primary cancer must be available for central testing. If not available or sufficient patients will be asked to undergo an US or CT guided biopsy prior to study entry to satisfy this eligibility criterion.
6. Locally advanced or metastatic disease which is measurable according to the Response Evaluation Criteria in Solid Tumours (RECIST v1.1)
7. Received no prior systemic therapy for metastatic or locally advanced disease. Prior adjuvant chemotherapy (with Gemcitabine or any other drug/s) is allowed if completed at least 6 months previously.

8. Adequate hematologic and end organ function, defined by the following laboratory results obtained within 14 days prior to the first study treatment:
    a. Absolute Neutrophil Count ≥ 1.5 x 10^9/l (without granulocyte colony-stimulating factor support within 2 weeks prior to the first study treatment)
    b. Platelet count ≥ 100 x 10^9/l (without transfusion within 2 weeks prior to the first study treatment)
    c. Haemoglobin ≥ 10 g/dl (transfusion permitted to establish target haemoglobin levels prior to the first study treatment)
    d. Calculated creatinine clearance (e.g. Cockcroft-Gault) ≥ 50 ml/min
    e. Bilirubin level ≤ 1.5 ULN (patients with known Gilbert disease who have bilirubin levels ≤ 3 x ULN may be enrolled). Patients must be able to undergo biliary stenting if required before or, if required, during the trial
    f. AST or ALT <2.5 x ULN or <5 x ULN in the presence of liver metastases
    g. Alkaline phosphatase (ALP) <2.5 x ULN or <5 x ULN in the presence of liver and/or bone metastases
    h. INR and aPTT ≤1.5 x ULN; this applies only to patients who are not receiving therapeutic anticoagulation; patients receiving therapeutic anticoagulation should be on a stable dose.
9. Female patients of child-bearing potential are eligible, provided they have a negative serum or urine pregnancy test within 7 days prior to the first dose of study treatment, preferably as close to the first dose as possible. All patients with reproductive potential must agree to use a medically acceptable method of contraception throughout the treatment period and for 1 month after discontinuation of ATRA and / or Gemcitabine/nab-Paclitaxel (whichever is the latest) and for 6 months after discontinuation for male patients. Acceptable methods of contraception include IUD, oral contraceptive, sub-dermal implant and double barrier (condom with a contraceptive sponge or contraceptive pessary). Micro-dosed progesterone preparations ("mini-pill") are an inadequate method of contraception during treatment with ATRA. If patients are taking this pill they should be instructed to stop and another form of contraceptive should be prescribed instead.
10. Able to follow protocol requirements as assessed by the Principal investigator.

4.2 Exclusion Criteria

A patient will not be eligible for inclusion in this study if any of the following criteria apply:

1. Patient has known brain metastases.
2. Patient has experienced a significant reduction in performance status between the screening/ baseline visit and within 72 hours prior to commencement of treatment as per trial protocol, and as per the Investigator's assessment.
3. Patients with pre-existing sensory neuropathy >grade 1
4. History of malignancy in the last 5 years, with the exception of:
   Patients with prior history of in situ cancer or basal or squamous cell skin cancer are eligible.
   Patients with other malignancies are eligible if they were cured by surgery alone or surgery plus radiotherapy and have been continuously disease-free for at least 5 years.
5. Patient has active, uncontrolled bacterial, viral, or fungal infection(s) requiring systemic therapy.
6. Patient has HIV, or active hepatitis B or C infection.
7. Patient has undergone major surgery, other than diagnostic surgery (i.e., surgery done to obtain a biopsy for diagnosis without removal of an organ), within 4 weeks prior to Day 1 of treatment in this study.
8. Patient has a history of allergy (including soya bean or peanut allergies) or hypersensitivity to any of the study drugs or any of their excipients, or the patient exhibits any of the events outlined in the

Contraindications or Special Warnings and Precautions sections of the products or comparator SmPC or Prescribing Information.

9. History of connective tissue disorders (e.g., lupus, scleroderma, arteritis nodosa).
10. Patient with a history of interstitial lung disease, history of slowly progressive dyspnoea and unproductive cough, sarcoidosis, silicosis, idiopathic pulmonary fibrosis, pulmonary hypersensitivity pneumonitis or multiple allergies.
11. Patient with high cardiovascular risk, including, but not limited to, recent coronary stenting or myocardial infarction in the past year.
12. History of Peripheral Artery Disease (e.g., claudication, Leo-Buerger's disease).
13. Patient has serious medical risk factors involving any of the major organ systems, or serious psychiatric disorders, which could compromise the patient's safety or the study data integrity.
14. Concurrent treatment with other experimental drugs or participation in another clinical trial with any investigational drug ≤30 days prior to study entry depending on the half-life of the investigational drug and/or guidance issued by the IMP manufacturer. Please contact the STARPAC Coordinating team for further information.
15. Patient is taking any prohibited concurrent medication, including Vitamin A supplements, and is unwilling to stop use prior to and during the trial. Refer to section 6.11.2.
16. Patient is pregnant, planning to become pregnant or breast feeding.
17. Patient has received a live vaccine within four weeks prior to receiving their first dose of study treatment.
18. Patient is unwilling or unable to comply with study procedures, as assessed by the Principal Investigator.

5 STUDY PROCEDURES AND SCHEDULE OF ASSESSMENTS

5.1 Patient Identification

Patients will be identified in multi-disciplinary team meetings or in out-patient clinics, usually by their clinical care team.

5.2 Informed consent procedure

Baseline: It is the responsibility of the Investigator, or a medically qualified and suitably trainned person delegated by the Principal Investigator to obtain written informed consent from each subject **prior** to participation in this study, following adequate explanation of the aims, methods, anticipated benefits and potential hazards of the study. Ample time must be given for consideration by the patient before taking part. Attempts will be made to arrange for an official hospital translator for any participant who is not competent or comfortable with communication in English. The translator will be asked to read through the Patient Information Sheet (PIS) and Consent Form and to translate each section for the participant. Written informed consent will only be obtained from those who the Investigator feels assured has understood the implications of participation in the study. The PI must also document the informed consent procedure in the patient’s medical notes.

New information during trial: If new safety information becomes available the CI will review the study, and update the PIS accordingly and resubmit for relevant regulatory approvals. The CI and the Safety Review Committee (SRC) will review the new safety information and assess whether an urgent meeting should be convened or whether this information can be reviewed at the next scheduled meeting. All subjects, including those already being treated, should be informed of the new information, given a copy of the revised PIS and asked give their consent to continue in the study. Patients will not be re-consented following amendments that do not affect safety or number of assessments / visits required.

5.3 Patient Registration

Principal Investigators (PIs) must keep a record of all patients screened for entry into this study, including those deemed ineligible after screening. Copies of the screening logs should be filed in the Site File. For each patient the primary reason for exclusion should be recorded. Diagnostic data obtained as part of the patient's standard care can be used to determine eligibility provided they fall within the protocol defined timelines. Written informed consent must be obtained prior to the patient undergoing any study specific procedures. After ensuring that a patient has consented to participate in the study an eCRF must be completed.

The STARPAC database will assign patients with a unique screening number. Once it is confirmed that a patient meets all eligibility criteria and the screening eCRF has been completed the CECM Coordinating Team will assign a Trial ID and provide details of the treatment allocation (dose level). To ensure patient confidentiality, patients will only be identified on eCRFs, other trial specific forms and all communication to CECM using their assigned Trial ID. It is the PI's responsibility to maintain a confidential record of the identity i.e. full name, date of birth and hospital number for the patients enrolled in this study and their assigned trial ID. At the end of the study this record should be archived along with the Investigator Site File (ISF). Full details of the patient enrolment procedure can be found in the STARPAC enrolment procedure manual.

Patients must receive their first dose of study drug within 7 days of being enrolled in the study.

5.4 Withdrawal of Patients / Discontinuation of Treatment

Patients must be withdrawn from study treatment for the reasons listed below:

- Disease progression
- Occurrence of intolerable side effects
- Intercurrent illness which prevents further treatment
- Patient choice
- Patient is pregnant
- Death
- Any alterations in the patient's condition which justifies the discontinuation of treatment in the investigator's opinion

Unless patients withdraw their consent, survival information and details of future anti-cancer treatments will continue to be collected as described in the table of assessments.

If the patient permanently discontinues study drug for a reason other than disease progression, such as toxicity, the state of disease in terms of progression should be noted at this time point. Ideally formal imaging should occur at this point to document the state of disease. Alternatively the previous scan should be used. Where possible scanning should continue as per normal practice until progression has been reached as per protocol. The date of progression of disease should be recorded in the CRF. Further treatment is at the treating doctor's discretion.

5.4.1 Withdrawal of Consent

Patients may withdraw their consent to participate in the trial at any time. If the patient explicitly states their wish not to contribute further data to the study, the investigator should inform the CECM co-ordinating centre in writing and the withdrawal of consent should be documented by the investigator in the relevant eCRF. However, data and samples collected up to the time of consent withdrawal will be included in the data reported for the study.

5.5 Study Assessments

Study assessments may be delayed or moved ahead of the window to accommodate holidays, vacations, and unforeseen delays. Please see Table 2 for schedule of assessments.

Table 2: Schedule of assessments.
Tests carried out every 8 weeks can be carried out with a ± 7 day window, whereas weekly tests can be carried out within a ± 3 day window.
Do not have to be repeated if carried out within 7 days from Cycle 1 Day 1. All assessments except PK to be carried out prior to treatment administration on the days that drug is given.

Day	Screening / Baseline (-28 to 0)	Cycle 1				Cycle 2 onwards			Safety visit[1]	PD[2]	Post Treatment FU[3]
		1	8	15	22	1	8	15			
**Informed consent**	X										
**Medical History, Demographics**	X										
**Weight (height screening only)**	X	*				X					
**Vital signs (HR, BP, temperature)**	X	*	X	X		X	X	X		X	
**ECOG, physical exam**[4]	X	*				X			X		
**AE assessment CTCAE v4.03**		X	X	X		X	X	X	X	X	X[5]
**Peripheral neuropathy assessment**	X										
**12-lead ECG**	X										
**Pregnancy test WOCBP**	X[6]					X[7]					
**Haematology & coagulation**[8 9]	X	*	X	X	X	X	X	X	X	X	
**Biochemistry** [6, 10]	X	*	X	X	X	X	X	X	X	X	
**HIV, HBV / HCV testing**	X										
**Vitamin A**	X	*				X[11]					
**CA19.9**	X	*				X					X[12]
**PK blood sample**[13]		X				C 2,3					
**Tumour assessment CT/MRI Scan**[14]	X	Every 8 weeks (±7 days) until PD								X	X[15]
**Concomitant medications**	X					X					
**Gem/nab-Paclitaxel administration**		X	X	X		X	X	X			
**ATRA dispensing**		X				X					
**ATRA Dosing adherence**				X				X			
**Buccal swab & hair follicle *(OPT)***		X		X		C3		C3		X	
**Archival Tumour Tissue (FFPE)**	X										
**Fresh tumour biopsy *(OPT)*** [16]	X					C3[17]				X	
**DW-MRI *(OPT)***[18]		X				C2,4					
**2 X 10 mL EDTA blood sample**	X	*	X	X		X					
**Survival and disease status data**											X

1 30days ± 7 days after the last dose of Gemcitabine/nab-paclitaxel.
2 Only if patient comes off treatment prior to disease progression. Can be via telephone if patient unable to attend hospital, in this case only AEs will be collected.
3 Patients should be followed up at 3 monthly visits as per standard care. Patients will be followed-up for up to 12months after enrolment.
4 Complete physical examination at screening, thereafter targetted physical examinations based on symptoms on D1 of each cycle.
5 Only for AEs unresolved after stopping IMP treatment
6 Within 7days prior to first study drug administration
7 Pregnancy test should be repeated on Day 1 of each cycle whilst receiving ATRA. Result should be confirmed negative prior to dosing.
8 Pre-dose samples may be drawn ≤ 7 days prior to IMP administration and do not have to be repeated as part of C1 D1 assessments if performed as part of screening assessments. C2 onwards pre-dose samples may be drawn ≤ 3 days prior to IMP administration.
9 Haematology: FBC (inc. Hb, WBC, ANC, and platelet count), and INR or PTT. FBC results must be obtained prior to treating the patient at all visits.
10 Biochemistry: D1 of each Cycle: UEs (Urea, Na, K, Cr), Ca, CRP, LFTs (ALT or AST, total bilirubin, ALP, albumin, total protein), GGT, Cholesterol, trigylcerides. UEs, LFTs, and GGT results must be obtained prior to starting treatment on Day 1 of each cycle.
Days 8 and 15 of each Cycle (and day 22 of Cycle 1): UEs (Urea, Na, K, Creatinine) and LFTs (ALT or AST, total bilirubin, ALP, albumin, total protein) only.
11 Vitamin A levels must be measured prior to ATRA dosing for that cycle.
12 Only if the patient attends hospital visits. If data collected via telephone contract then this assessment can be omitted.
13 Details on blood sampling regimen for PK are given in tables 4 & 5.
14 Screening CT Scan of chest, abdomen, and pelvis, with additional anatomy as clinically indicated by extent of disease ≤ 28 days D 1 C 1. Subsequent CT scans of the chest, abdomen and pelvis and other sites of disease every 8 weeks thereafter (± 7 days).
15 Only for patients who withdrew from IMP for reasons other than disease progression. These patients will be scanned until disease progression.
16 Optional except when the archival FFPE tumour sample is insufficient, then a biopsy is mandatory prior to study entry.
17 Tumour Biopsy from any disease site at D1 C3 (± 7 days) if tissue can be obtained without a general anaesthetic, FBC must confirm ANC>1.3x10^{9}/L, platelets>75x10^{9}/L, coagulation PT/APTT<1.5xULN to proceed with biopsy. If parameters not met, the biopsy will be not be done.
18 Optional DW-MRIs cans will have a 'coffee break' scan (two scans with 30min break) ≤14days prior to D1 C1, and single scans at the end of C1 and C3 (between days 22-28).

5.5.1 Medical History and Demography

A complete medical history will be obtained including details of any relevant medical conditions occurring prior to consent. Details will be collected on the patient's cancer diagnosis including site, date of diagnosis, radiological tumour size, grade, lymph node status, prior anti-tumour treatment (if any) and outcome, medications and their indications. Patients should be evaluated for familial, environmental or occupational exposure to opportunistic pathogens (see section 6.12.6). Demographic data collected will include sex, date of birth and race/ethnicity.

5.5.2 Weight, height, vital signs, ECOG performance score and physical exam

Height (cm) will only be measured at screening. Weight (kg) and temperature (degrees Celsius) will be measured at the time points indicated in the table of assessments. Heart rate, systolic and diastolic blood pressure should be measured while the patient is in a seating position. Performance status will be assessed at every visit using the ECOG performance score below and will be recorded in the e-CRF.

Grade	ECOG
0	Fully active, able to carry on all pre-disease performance without restriction
1	Restricted in physically strenuous activity but ambulatory and able to carry out work of a light or sedentary nature, e.g., light house work, office work
2	Ambulatory and capable of all self-care but unable to carry out any work activities. Up and about more than 50% of waking hours
3	Capable of only limited self-care, confined to bed or chair more than 50% of waking hours
4	Completely disabled. Cannot carry on any self-care. Totally confined to bed or chair
5	Dead

A complete physical examination will be carried out by a physician at screening. Thereafter, targetted physical examinations will be carried out based on patient symptoms at the timepoints indicated in the table of assessments. The outcome of the examination will be assessed as normal or abnormal, if abnormal then details should be recorded on the relevant visit.

5.5.3 Pregnancy test

Female patients who are of childbearing potential must have a negative serum or urine test within 7 days prior to and preferably as close as possible to the first dose of treatment. Pregnancy tests must be repeated on Day 1 of each cycle whilst receiving ATRA. The discussion about contraception should be recorded in the medical notes.

5.5.4 Blood tests

The following haematology and biochemistry assessments will be carried out at the local site's laboratory using standard methods for routine tests at the time points described in the table of assessments. Cycle 1 Day 1 pre-dose laboratory samples may be drawn up to 7 days prior to study drug administration and do not have to be repeated as part of Cycle 1 Day1 assessments if performed within 7 days of Day 1 Cycle 1 as part

of the screening assessments. For Cycle 2 onwards pre-dose laboratory samples may be drawn up to 3 days prior to the scheduled visit and study drug administration.

The following variables will be measured:

Haematology: Full blood count (including haemoglobin, WBC, ANC, and platelet count) and INR or PTT. <u>The results of the FBC must be obtained prior to treating the patient at all visits.</u>

Biochemistry:

<u>Day 1 of each Cycle:</u>

UEs (Urea, Na, K, Creatinine), Calcium, CRP, LFTs (ALT or AST, total bilirubin, ALP, albumin, total protein), GGT (Gamma glutamyl transferase), cholesterol, trigylcerides.

<u>Results for UEs, LFTs, and GGT must be obtained prior to starting treatment on Day 1 of each cycle.</u>

<u>Days 8 and 15 of each Cycle (and day 22 of Cycle 1):</u>

UEs (Urea, Na, K, Creatinine), LFTs (ALT or AST, total bilirubin, ALP, albumin, total protein)

- *<u>CA19.9:</u>* To be measured at Screening and Day 1 of each cycle. If patient is unable to attend hospital for their post treatment follow-up visit this measurement can be ommited.
- *<u>Vitamin A level</u>*: To be measured at Screening and prior to Day 1 of each cycle. Must be measured prior to beginning dosing for that cycle. ATRA dose for part 2 may be altered according to Vitamin A serum levels measured in order to achieve the required OBD of Vitamin A in the patient. Instructions for dosing adjustments will be established using data from part 1 of the study.

Immunology: HIV, HBV or HCV at screening / baseline only

5.5.5 ECG Measurements

Baseline 12-lead ECG readings will be taken during the screening period. It is important that patients are resting quietly for at least 5 minutes prior to recording ECGs. Blood draws and other procedures should be avoided during the period immediately before ECG measurement, and activity should be controlled as much as possible to minimize variability due to the effects of physiologic stress.

5.5.6 Adverse Event Assessment

Adverse Events (AEs) will be collected throughout the study, from the time the patient gives informed consent to the end of treatment visit and recorded in the relevant eCRF.

The following details will be collected:

- AE term,
- date of onset,
- date of resolution (or if ongoing),
- NCI CTCAE grade (maximum intensity),
- Seriousness,
- investigator causality rating against the study medication (yes or no),
- action taken with regard to study medication,
- outcome.

The Investigator should evaluate all adverse events and should make an immediate effort to determine their aetiology. Study medications may be interrupted for ≤28days(1 cycle) for an adverse event at the discretion of the Investigator. If treatment is delayed for >28 days then the patient must discontinue study treatment. Patients with an unresolved AE or SAE at treatment completion or study drug discontinuation will be contacted by the investigator or their designee to determine the status of the event until the event is resolved or stabilised, the patient is lost to follow up or it has been determined that the study treatment or participation is not the cause of the event. These details will also be recorded on the eCRF. If a patient experiences any of the following AEs, then the investigations outlined in Table 3 should be carried out. Details of these investigations should be recorded on the relevant eCRF.

Table 3: Additional assessments triggered by specific AEs

Adverse Event	Assessment
Febrile Neutropenia	Repeat blood tests on day 3 after discovery of the AE and if still present repeat again on day 7 to ascertain recovery pattern.
Grade 4 Neutropenia	Repeat blood tests on day 3 after discovery of the AE and if still present repeat again on day 7 to ascertain recovery pattern. If resolved within 8 days such a Grade 4 event, it will not be considered DLT.
Grade 4 anaemia	Repeat blood tests on day 3 after discovery of the AE and if still present repeat again on day 7 to ascertain recovery pattern. If resolved within 8 days such a Grade 4 event, it will not be considered DLT.
Grade 4 thrombocytopenia	Repeat blood tests on day 3 after discovery of the AE and day 7 to ascertain recovery pattern. If resolved within 8 days such a Grade 4 event, it will not be considered DLT.
>Grade 3 Neuropathy	Repeat neurological assessment examination on a weekly basis after discovery of the AE until resolution to Grade 2 or below
Suspected pneumonitis or any any new or worsening respiratory symptoms (eg. cough, dyspnoea, lower respiratory infection) persistant for >14 days without clinical symptoms of airway infection or disease progression	• Complete pulmonary function test including 3 forced expiratory volumes, forced vital capacity and carbon monoxide diffusing capacity (DLco% & DLco). A recent haemoglobin measurement should be available at the time of the DLco evaluation.<br>• CT scan of the chest (ideally a high-resolution CT scan) as per normal practice.

5.5.7 Pharmacokinetic Analysis (ATRA)

Frequency of PK analysis: Blood for the pharmacokinetic profiling of ATRA will be collected on patients enrolled into enrolled into both parts of the study, on day 1 of each of the first three cycles. In addition, blood sampling for ATRA PK analysis will be performed on part 2 patients for the first three cycles plus any cycles for which the ATRA dose has been modified (Table 4). Other metabolites of Gemcitabine and nab-paclitaxel (e.g., dFdU, dFDCTP, dFdC) may also be determined.

Exceptions and adjustments: On days and time points when blood samples for biomarkers and pharmacokinetics are to be performed, the pharmacokinetic sample must be drawn first. All pre-dose samples should be taken within 15 minutes before dosing.

Method: At the specified time points, 2 ml of venous blood should be collected. Please refer to the STARPAC Laboratory Manual for information on processing, storage and transport details. The exact date and time of each PK sample and dose on each PK day must be recorded on the CRF. Samples for the determination of ATRA concentrations in plasma will be analyzed by using an appropriate bioanalytical method. Full details of the analytical method used will be described in a separate bioanalytical report.

Table 4: Blood collection plan for PK profiling. Pre-dose samples should be taken within 15 minutes before ATRA dosing.

Schedule	Part 1	Part 2
Day 1, Cycle 1	Pre-dose, 0.5, 1, 2, 3, 4, 5h post-dose	Pre-dose, 0.5, 1, 2, 3, 4, 5h post-dose
Day 1, Cycle 2	Pre-dose, 0.5, 1, 2, 3, 4, 5h post-dose	Pre-dose, 0.5, 1, 2, 3, 4, 5h post-dose
Day 1, Cycle 3	Pre-dose, 0.5, 1, 2, 3, 4, 5h post-dose	Pre-dose, 0.5, 1, 2, 3, 4, 5h post-dose
Day1, any cycle where ATRA dose is altered		Pre-dose, 0.5, 1, 2, 3, 4, 5h post-dose

5.5.8 Tumour assessments

RECIST v1.1 criteria will be used to assess patient response to treatment by determining disease control rate, PFS, objective response rate (ORR), , percentage of patients without progressive disease at 8 weeks, and disease control rate. The RECIST v1.1 guidelines for measurable, non-measurable, target, and non-target lesions (NTL) and the objective tumour response criteria (CR, PR, stable disease [SD], or progressive disease [76]) are presented in Appendix 1.

Tumour assessments should be performed at screening (within 28 days prior to Day 1 of Cycle 1), every 8 weeks thereafter (+/-7 days), and when clinically indicated for all patients. This schedule is to be maintained and will not be shifted for treatment delays. A documented standard-of-care tumour assessment performed within 28 days before Day 1 of Cycle 1 may be used for the screening assessment provided it meets the above requirements. The same imaging method used to define disease sites at screening must be used throughout the study (e.g., the same contrast protocol for CT scans). Response assessments will be performed by the investigator, on the basis of physical examinations and imaging scans, through use of RECIST v1.1. Patients who discontinue study treatment for any reason other than disease progression will continue to undergo tumour-response evaluations until progressive disease or initiation of other anti-cancer therapy.

Screening assessments must include CT scans of the chest, abdomen, and pelvis, with additional anatomy as clinically indicated by extent of disease. Subsequent tumour assessments should include CT scans of the chest, abdomen and pelvis and other sites of disease. MRI scans may be substituted for CT scans but MRI of the chest may only be performed with approval from the STARPAC CECM Coordinating Team. Additional anatomy may be imaged at follow-up on suspicion of new lesions.

Additional scans: Any other sites at which new disease is suspected should also be appropriately imaged. If an unscheduled assessment was performed and the patient has not progressed, every attempt should be made to perform the subsequent assessments at their scheduled visits. CT scan of the chest (ideally a high-resolution CT scan) is required in case of suspected pneumonitis or any new or worsening significant

respiratory symptoms persistent for >14 days without clinical symptoms of airway infection or disease progression

Evaluation: Equivocal new lesions may be further evaluated by other modalities (biopsy, MRI, or CT scans, or plain radiographs), however, these are not study specific investigations. If the lesions remain equivocal, the investigator should use his or her judgment with regard to recording the finding as a new lesion on the Tumour Evaluation CRF. If a new lesion is recorded, the tumour response should be recorded as progressive disease. If the lesion remains equivocal and in the investigator's opinion is likely not reflective of progressive disease, the lesion should not be recorded as a new lesion, and the patient may remain on study drug. This applies to new lesions identified by any tumour assessment modality. If a lesion is seen in anatomical region which was not imaged at baseline, this should be considered as a new lesion.

Categorisation of objective tumour response assessment will be based on the RECIST v1.1 criteria of response: CR, PR (partial response), SD (stable disease) and PD (progression of disease). Target lesion (TL) progression will be calculated in comparison to when the tumour burden was at a minimum (i.e. smallest sum of diameters previously recorded on study). In the absence of objective progression, tumour response (CR, PR, SD) will be calculated in comparison to the baseline tumour measurements obtained before starting treatment.

If the Investigator is in doubt as to whether objective progression has occurred, particularly with response to NTL or the appearance of a new lesion, it is advisable to continue treatment until the next scheduled assessment and reassess the patient's status. Alternatively, repeat assessments might be scheduled earlier if clinically indicated. If repeat scans confirm progression, then the date of the initial scan should be declared as the date of objective progression.

To achieve 'unequivocal progression' on the basis of non-target disease, there must be an overall level of substantial worsening in non-target disease such that, even in presence of SD or PR in target disease, the overall tumour burden has increased sufficiently to merit discontinuation of therapy. A modest 'increase' in the size of one or more NTLs is usually not sufficient to quality for unequivocal progression status.

5.5.9 Concomitant medication

All medications (including prescription medications and over the counter preparations), taken by the patient from the day of consent to the end of study treatment visit will be documented as concomitant medications. Note all medication should be recorded to clearly to allow for identification of prohibited medications. If a patient is taking any of the medications defined as prohibited for use during the study according to section 6.12, then these will be documented during screening. Patients must stop taking any prohibited medications prior to starting study treatment within the specified wash out periods of the prohibited medications (see section 6.11.2). The following details will be collected at the beginning of each cycle: drug name, reason for treatment, dose/units, route of administration, frequency, start and end date of therapy.

5.5.10 Study drug Compliance (patient diaries)

Patients will keep a detailed record of all study medication taken in their patient diaries. Date and time of administration, drug name and drug dose will be collected.

5.6 Exploratory Research

All samples collected as part of the study will be used for exploratory research, and any samples remaining at the end of the study may be stored in Barts Pancreas Tissue Bank and used for future ethically approved research.

Biomarkers are objectively measured and evaluated indicators of normal biological processes, pathogenic processes and/or pharmacologic responses to a therapeutic intervention. All patients will be consented for the collection of archival FFPE tumour samples as well as research blood samples before and during treatment with ATRA and Gemcitabine and nab-paclitaxel as detailed in the table of assessments. This part of the study is mandatory.

Patients will also be given the option to donate additional tumour samples and normal, surrogate tissue (buccal mucosa epithelial cells and hair follicles) samples taken before and after treatment. This part of the study is optional.

These biomarker assessments will potentially aid in understanding the drug combination effect at both the molecular level and on clinical outcome. The obtained biomaterial may be stored for future research related to this study and other studies in pancreatic cancer. All samples will be linked anonymised and only identified by the trial ID and unique sample number allocated by the STARPAC CECM Coordinating Team. The samples will be stored according to Barts Pancreas Tissue Bank SOP. These results may be reported separately from the clinical study report.

Biomarker development is an exploratory goal in this study and two strategies will be employed: serum derived and imaging biomarkers. In a multi-centre cohort of over 280 patients and respective controls, work carried in Professor Kocher's laboratory, Queen Mary University of London has demonstrated that Serum Pentraxin3 (PTX3) had superior Area under Curve (0.91) and Positive Predictive value (0.98) over either CA19-9 or CEA, thus establishing it as a valid diagnostic and predictive marker [77]. Furthermore, in organotypic cultures and mice it was demonstrated that tissues expression of PTX3 remarkably decreases upon treatment with ATRA. In human PDAC patients, Vitamin A and PTX3 levels are inversely correlated. This lends to the hypothesis that serum PTX3 is a measure of stromal activity. Serum PTX3 may act as a surrogate marker for stromal activity in human PDAC. This exploratory hypothesis will be tested in STARPAC. Furthermore, other serum markers of PDAC stromal activity such as TGFβ and FGF2 will be developed within Professor Kocher's laboratory, Queen Mary University of London [60].

5.6.1 Archival Primary Tumour Samples (Mandatory)

Archival tissue samples (primary cancer excision/biopsy and any metastasis biopsy samples), which were obtained as part of normal clinical care, will be collected for each patient in the form of FFPE tumour blocks. If it is not possible to obtain the tumour tissue paraffin blocks, at least 10 slides of freshly prepared unstained 5 micron sections may be collected instead. These are archived samples and as such participating patients will not need to attend extra visits or undergo extra procedures. If archival tumour tissue is either insufficient (minimum of 2mm x 2mm in size) or unavailable, the patient will be required to give a fresh biopsy (US or CT guided) prior to starting study treatment in order to still be eligible for the study. This sample must be obtained prior to study entry.

5.6.2 Additional biopsies (Optional)

Patients will be offered the opportunity to have additional tissue taken from the primary tumour or a metastatic lesion (US or CT guided) as part of the trial, during screening, Day 1 Cycle 3 (+/- 7 days) independent of timing of taking ATRA and Gemcitabine and nab-paclitaxel and at disease progression, if applicable. These additional biopsies are optional. At each time-point, wherever feasible, two core biopsies should be obtained. If feasible, both FFPE and frozen biopsies should be obtained, but FFPE biopsies alone are also acceptable. The sample collection date, the exact time of collection, and the time of exposure to fixative (formalin) must be entered on the appropriate eCRF . Please refer to the STARPAC Laboratory Manual for further information on sample processing, handling and shipment.

5.6.3 Research Blood Samples (Mandatory)

All patients will be asked to donate 2 x 10mL whole blood samples in EDTA Vacutainers for future ethically approved research. This part of the study is not optional. Further details on sample processing, handling and shipment are provided in the study manual.

5.6.4 Buccal swab and hair follicles (Optional)

Buccal swab and hair follicle samples will be collected for biomarker studies, to examine the pharmacodynamic (PD) effects and changes that occur over time. Collection is optional and samples will be taken on Day 1 of cycle 1 prior to ATRA administration and 5 hours post ATRA administration (in parallel with PK studies) and on Day 15 of cycle 1 when final dose of ATRA has been taken. To assess changes occuring over time, further samples will be collected on Day 1 and Day 15 of cycle 3 and at disease progression, if applicable. Sample collection will include:

- Buccal swab (buccal mucosal epithelial cells)
- Hair follicles (hair sheath cells)

The outer root sheath of the hair follicle, which is composed of 5 specific layers of epithelial cells, is continuous with the basal layer of the epidermis and the importance of retinoic acid signalling in hair development and maintenance is well documented.[73] RARβ is weakly expressed in follicle keratinocytes and outer root sheath cells and changes upon treatment with ATRA will be measured. In addition to RARβ expression, changes in expression of other genes involved in RA metabolism and genes known to be affected by ATRA treatment based on laboratory research (e.g. CRABP2, sFRP4, FGF2, CXCL12) carried out by Prof Kocher's group at Queen Mary University of London will be assessed. As for buccal mucosal epithelial cells, 13-cis RA has been shown to increase the expression of RARβ in buccal mucosal cells and epithelial cells obtained from buccal smears may provide a useful means to assess the PD effects of ATRA and assess biological markers of response.[74, 78] This hypothesis will be tested in the current clinical trial and subsequent translational research.

Hairs will be collected from the scalp (head hair) or eyebrows using a blunt-nosed forceps. Buccal smears will be obtained by brushings. The sample collection date and the exact time of collection must be entered on the appropriate eCRF. Please refer to the STARPAC Laboratory Manual for further information on sample processing, handling and shipment.

5.6.5 Biosample Assays

In keeping with the study objectives, multiple assays may be performed with the material derived from each patient (if available). It is likely that not all assays will be performed on samples provided by each patient (possibly because of insufficient tumour material or inadequate sample quality). Exploratory analysis may include, but will not be limited to, the following:

- Tissue ATRA level
- Immunohistochemistry (IHC) or immunofluorescence (IF) for PTX3 (unpublished data), FGF2, FGFR1[60], FABP5, CRABP2[69], sFRP4, β-catenin, RARβ [21].
- Blood samples will be used for measurement of candidate biomarkers, using quantitave measurement by ELISA for PTX3, FGF2 and other biomarkers of interest. In addition, PBMCs will be collected for PD analyses, using various techniques including Western immunoblotting and/or IHC or IF.
- Mutational analysis using using PCR (hotspot) and/or sequencing techniques from tissue specimens and/or from circulating cell-free DNA (cfDNA). DNA will be extracted from tumour samples and subjected to quantitative RT-PCR (qRT-PCR) assays that detect the wild-type allele as well as assays for nucleotide substitutions. Following histopathological review, samples with <20% tumour content will be enriched for tumour content by macro- or micro-dissection. In addition to DNA extraction from tumour samples, cfDNA will be extracted from plasma samples collected from patients and will be used for the detection of oncogenic mutations.

5.6.6 Chain of Custody of Biological Samples

In all cases, patients will be consented for the collection and use of their biological samples and a full chain of custody will be maintained for all samples throughout their lifecycle. The Investigator at each site is responsible for maintaining a record of full traceability of biological samples collected from patients while these are in storage at the site, either until shipment or disposal. Any sample receiver e.g. sub-contracted service provider will keep full traceability of samples from receipt of arrival to further shipment or disposal (as appropriate).

In the event that a patient withdraws their consent from the study all samples and data collected up to that date will be used in the study but no further data will be collected.

Barts Health NHS Trust as the Sponsor will keep overall oversight of the entire lifecycle through internal procedures and monitoring of study sites, the Trial Chairman will be the custodian of the samples. Diagnostic tumour blocks will be returned to source at the end of the study or, upon request, earlier if required for the patient's clinical management. Research tumour samples and cut sections will be retained by the study team. Diagnostic tumour blocks which are not required to be returned to the source at the end of the study will also be retained by the study team. Samples retained for further ethically approved research use will be registered with the Barts Pancreas Tissue Bank (REC Ref: 13/SC/0592, HTA Licensing number: 12199), Queen Mary University of London. Samples may be transferred to organisations (including commercial organisations) outside the EU for analysis.

PK samples will be disposed of after the Bioanalytical Report finalization or six months after issuance of the draft Bioanalytical Report (whichever is earlier), unless requested for future analyses. PK samples may be disposed of or destroyed and anonymised by pooling. Additional analyses may be conducted on the anonymised, pooled PK samples to further evaluate and validate the analytical method. Any results from such analyses may be reported separately from the CSR. Anonymised PK samples will be retained for no more

than 5 years after the study report is finalized. Incurred sample reproducibility analysis, if any, will be performed alongside the bioanalysis of the test samples. The results from the evaluation will not be reported in the CSR but separately in a Bioanalytical Report.

5.6.7 DW-MRI assessments

Patients will be requested to participate in the DW-MRI translational part of this study. In this part, the utility of DW-MRI (including ivim) to detect treatment response at an early time point after (one cycle) and late (three cylces) compared to baseline (which includes a reproducibility scan for ADC and b-values) will be investigated. DW-MRI scans will be carried out at each participating site in accordance with the DW-MRI Imaging Manual. Each site will perform a test scan transfer prior to activation. This test will include transfer and quality scan elements. Patients will undergo a 'coffee break' scan (consisting of two scans to check reproducibility) within 14 days prior to starting treatment on Day1 of Cycle 1 (baseline) and single scans at the end of cycles 1 and 3 (days 22-28). Scans will be carried out at the local institution and copies of the scans will be transfered to the Institure of Cancer Research/ Royal Marsden Hospital Foundation NHS Trust for central assessment. Further details of the imaging procedure and transfer of the scans can be found in the STARPAC MRI Imaging Manual.

5.7 Follow-up, Survival and Anti-cancer therapy

Survival information and details of future anti-cancer treatments will be collected for each patient 3 monthly over a 12month period from the time of registration..

6 INVESTIGATIONAL MEDICINAL PRODUCT

Refer to STARPAC Pharmacy Manual for further details.

6.1 Definition of the IMP

ATRA, Gemcitabine and nab-Paclitaxel are all considered to be IMPs in this study. ATRA will be used outside of it's licensed indication for this study. Gemcitabine and nab-Paclitaxel in combination is licensed for first-line treatment of adult patients with metastatic adenocarcinoma of the pancreas, however, as the doses administered to patients in the study may be below the recommended therapeutic dose as per the SmPC, these are also considered to be IMPs.

Refer to the relevant current version of the SmPCs for information regarding the physical and chemical properties and the lists of excipients of ATRA, Gemcitabine and nab-paclitaxel.

6.2 Product Sourcing, Manufacture and Supply

Commercial Gemcitabine and ATRA (also known as Tretinoin) will be obtained locally by the investigating sites in keeping with standard local practice. Nab-paclitaxel will be supplied by Celgene Sarl Inc.and labelled and distributed to participating sites by Fisher Clinical Services.

6.3 Packaging and Labelling

IMP label templates will be provided to participating sites for local labeling by the respective Pharmacy departments.

Nab-Paclitaxel will be packaged and labelled in accordance with local regulations and Good Manufacturing Practice Annex 13, stating that the drug is for clinical trial use only and should be kept out of the reach of children. Nab-paclitaxel will be distributed by Fisher Clinical Services and supply will be managed by them in conjunction with the Centre for Experimental Cancer Medicine.

6.4 Receipt of IMP Supplies

Nab-paclitaxel will be received by the pharmacy department at the participating site. The Pharmacy department should acknowledge receipt of the Nab-Paclitaxel as per the STARPAC Pharmacy Manual. Receipt of ATRA and Gemcitabine will be carried out and documented as per local practice.

Drug accountability for all study drugs will be recorded on a study-specific accountability log. The accountability log will be kept in the Pharmacy Site File.

6.5 Drug Storage

All investigational products must be kept in a secure place under appropriate storage conditions. A description of the appropriate storage and shipment conditions are specified on the investigational labels. Study treatment must be kept out of the reach of children. The PI at each participating site or a delegated person e.g. pharmacist, is responsible for ensuring that the IMPs are stored in a secure place and under the recommended storage conditions.

Gemcitabine and ATRA will be stored at room temperature and in line with local & SmPC requirements.

Nab-Paclitaxel will be stored at 15-25°C.

Please refer to the pharmacy manual for further details.

6.6 Drug Accountability

The investigator or a delegated individual (e.g. pharmacist), must ensure that the study drugs are stored and dispensed in accordance with hospital standard operating procedures and applicable regulatory requirements. Full drug accountability records must be maintained for all three IMPs using the IMP accountability logs provided. Sites may amend the IMP accountability logs provided or use their own documentation if it captures all the information required by the Sponsor. The medication provided for this study is for use only as directed in the protocol. Drug distribution and accountability logs will be provided to the site in a pharmacy pack. It is the investigator’s responsibility to establish a system for handling the investigational product to ensure that:

- Deliveries of investigational products are correctly received by a responsible person (e.g., pharmacist or suitable pharmacy designee) and are handled and stored correctly and safely
- Investigational products are dispensed only to study participants, and in accordance with the protocol
- Participants return any unused investigational product and all empty containers to the investigator/pharmacy
- A dispensing record (which will include the identification of the participant to whom the investigational product was dispensed, the date of dispensing, the quantity of investigational product dispensed, and the date and quantity of any unused investigational product returned to the pharmacy) is accurately maintained. Any discrepancies must be accounted for on the appropriate form. This record is in addition to any drug accountability information recorded in the CRF.

In the case that nab-Paclitaxel is damaged, please contact the Centre for Experimental Cancer Medicine for reconciliation and replacement. Patients will be given a diary card to keep track of ATRA. At the termination of the study or at the request of the sponsor, all unused drugs will be accounted for and destroyed locally at the study sites as per local policies. Certificates of delivery and destruction or return must be signed and copies retained in the Pharmacy Site File. Further guidance will be provided in the STARPAC Pharmacy Manual.

6.7 Dispensing of IMP

ATRA, Gemcitabine and nab-Paclitaxel will be dispensed by the pharmacy at the participating site in accordance with the trial-specific prescription. A prescription will be provided in the pharmacy file, although sites will be permitted to use their own clinical trial template prescription if suitable. Prior approval of the final prescription template by the Sponsor is required. Dose banding according to local institution policy is acceptable for Gemcitabine, to a variance of +/- 5%. ATRA will be dispensed on Day 1 of each cycle of treatment. Gemcitabine and nab-Paclitaxel will be dispensed on Days 1, 8, and 15 of each cycle of treatment. Patients will be supplied with sufficient ATRA for that cycle of treatment (for Days 1-15).

6.8 Treatment regimens and dosing levels

6.8.1 Part 1

STARPAC will investigate the following dosing schedules:

**Dose level**	**Gemcitabine**		**Nab-Paclitaxel**		**ATRA**	
**D1**	80%	800 mg/m^2	80%	100 mg/m^2	33%	15 mg/m^2
**D2**	80%	800 mg/m^2	80%	100mg/m^2	66%	30 mg/m^2
**D3**	80%	800 mg/m^2	80%	100 mg/m^2	100%	45 mg/m^2
**D4**	100%	1000 mg/m^2	80%	100 mg/m^2	100%	45 mg/m^2
**D5**	100%	1000 mg/m^2	100%	125 mg/m^2	100%	45 mg/m^2

Treatment doses will be assigned at registration. Patients will receive ATRA, Gemcitabine and nab-Paclitaxel in 28 day cycles. ATRA will be administered for 6 cycles whereas Gemcitabine/nab-Paclitaxel will be administered until disease progression. Treatment may be discontinued earlier due to unacceptable toxicities or death or because the patient requests to be withdrawn from study treatment. If treatment with Gemcitabine/nab-Paclitaxel is stopped prior to the patient completing 6 cycles of treatment with ATRA, the patient may continue on treatment with ATRA alone until the 6 cycles are completed, at the discretion of the treating physician.

Doses and the dose escalation scheme may be modified according to emerging data from SAE monitoring for DLT as well as the PK analysis.

6.8.2 Part 2

At the end of part 1, a recommended part 2 dose will be defined based on the MTD, but might also consider the toxicity profile beyond cycle 1 and pharmacodynamics and pharmacokinetic markers. ATRA doses may

also be altered according to measurement of serum Vitamin A levels of patients at the end of each Cycle. If serum Vitamin A is not at OBD then ATRA dose may be altered accordingly to achieve the required Vitamin A level. Patients will receive ATRA, Gemcitabine and nab-Paclitaxel in 28 day cycles. ATRA will be administered for 6 cycles whereas Gemcitabine/nab-Paclitaxel will be administered until disease progression. Treatment may be discontinued earlier due to unacceptable toxicities or death or because the patient requests to be withdrawn from study treatment. If treatment with Gemcitabine/nab-Paclitaxel is stopped prior to the patient completing 6 cycles of treatment with ATRA, the patient may continue on treatment with ATRA alone until the 6 cycles are completed, at the discretion of the treating physician.

6.9 Study Drug Administration

Patients in the trial should begin their allocated treatments within 7 days from the date of enrolment. Administration of the chemotherapy drugs, Gemcitabine and nab-Paclitaxel will be performed on an outpatient basis (chemotherapy day-unit) on Days 1,8 and 15 of each cycle of treatment. The study is open labelled.

Oral ATRA will be self-administered by the patient at dose of 15-45 mg/m^2 on day one to fifteen (inclusive) of each of the treatment cycles in two divided doses rounded to nearest 10 mg and taken 12 hours apart. Please refer the STARPAC Pharmacy manual for further details on ATRA dosing. The first dose of each cycle will be administered in hospital for pharmacokinetic assays. It is recommented that ATRA capsules are taken with a meal or shortly thereafter. If vomiting occurs within 30 minutes after ATRA dosing, or later if the tablet(s) can be identified in the vomit content, the patient can re-take new capsules(s).

Should a patient miss a scheduled dose, the patient will be allowed to take the dose up to a maximum of 6 hours after the scheduled dose time. If greater than 6 hours after the scheduled dose time, the following recommendations apply:

- For the first dose in a cycle, the missed dose should not be taken and the patient should take the next allotted dose on the same day. Since this dose is monitored for first three cycles in Day-care for PK analysis, this is unlikely to occur.
- For the intervening doses in a cycle, the missed dose should not be taken on the scheduled day and patients should continue treatment with the next alloted dose. The missed dose should be taken at the end of the cycle.
- For the last dose of a cycle, the missed dose may be taken upto 24 hours after the allotted time.

If a patient needs to take the dose earlier for whatever reason, the patient can take the dose up to 2 hours earlier than the scheduled dose time, except for the dose for PK analysis (first dose of each cycle).

Intravenous (IV) nab-Paclitaxel 100-125 mg/m2 30-minute infusion followed immediately by IV Gemcitabine 800-1000 mg/m2 30-minute infusion will be administered on days 1, 8 and 15 of a 28 day cycle. Body surface area (BSA) will be calculated as per local practice. BSA should be recalculated on D1 of each cycle in the case of weight variation of greater than 5%. Safety assessments will be carried out as per schedule of assessments and on ad hoc basis if required clinically. All medications are in routine clinical use. It is the responsibility of the investigator to ensure that recommendations in the UK Summaries of Product Characteristics (SmPCs) are followed. Sites will be supplied with the current version of the SmPC for each study IMP. Any updates will be provided to sites.

6.10 Return or Destruction of IMP

Any study drug returned by patients, expired or remaining at the end of the trial must be destroyed according to the sites local standard operating procedures and only following permission by the Sponsor. Certificates of destruction must be provided by the site and copies retained in the Pharmacy Site File.

6.11 Concomitant Therapies

Patients must be instructed not to take any medications, including all over-the-counter products such as vitamins, minerals, and other dietary supplements, without first consulting with their site Principal Investigator.

Patients should receive full supportive care during and after the administration of chemotherapy. Over the course of this trial, additional medications may be required to manage aspects of the disease state of the patients, including side effects from trial treatments or disease progression. Details of the concomitant medication given, including blood and blood products, must be recorded in the patient's medical records and the eCase Report Form (eCRF). Supportive care, including but not limited to anti-emetic medications, may be administered at the discretion of the site Principal Investigator, as medically indicated.

6.11.1 Recommended concurrent therapy

The following therapies are recommended during study participation, as applicable:

- All non-cancer treatments that the responsible physician feels are appropriate are allowed.
- Standard anti-emetic therapy including a 5-HT3-antagonist can be given as needed on a prophylactic and treatment basis in compliance with the standards of the centre.
- Loperamide for symptomatic treatment of diarrhoea $\geq$grade 2 (details provided in section 6.13.6.)
- Blood transfusions are allowed at any time during the study. Patients already receiving erythropoietin at the time of screening for the study may continue it providing they have been receiving it for more than one month at the time study treatment is started.
- Patients may receive bisphosphonate or denosumab therapy for the treatment of bone metastases, but initiation of bisphosphonate or denosumab therapy or modification of the pre-study bisphosphonate or denosumab treatment regimen for bone metastasis requires the approval of the Medical Monitor.
- Patients may take low dose corticosteroids (<10 mg prednisolone or an equivalent dose of other anti-inflammatory corticosteroids), however, increased vigilance is recommended on electrolyte and/or glucose levels due to the potential for corticosteroid-related metabolic disturbance.
- Supportive care and other medication, which is considered necessary for the patient's safety and well being, may be given at the discretion of the investigator and recorded in the appropriate sections of the CRF.
- Growth factors and prophylactic antibiotics to prevent infection should be offered to patients continuing on treatment following a first episode of febrile neutropaenia.
- Prophylactic antibiotics are recommended for patients following any first febrile episode, especially if they have indwelling biliary stent(s).
- Administration of prophylactic antibiotics to otherwise uncomplicated patients with biliary stents will be at the discretion of the treating physicians. Biliary stents should be monitored closely to determine need for replacement.
- Additionally, any diagnostic, therapeutic or surgical procedure performed during the study period, should be recorded including the date, indication, description of the procedures(s) and any clinical findings.

- If medically feasible, patients taking regular medication should be maintained on it throughout the study period with the exception of potent or moderate inhibitors or inducers of CYP3A4/5 or CYP2C8 (See section 6.11.2).

6.11.2 Prohibited concurrent therapy

- Radiotherapy should only be offered to patients while chemotherapy is ongoing for eg. Symptomatic bone metastasis at principal invesitgator's discretion and will be recorded in eCRF.
- As with other retinoids, ATRA must not be administered in combination with vitamin A because symptoms of hypervitaminosis A could be aggravated.
- Systemic treatment with retinoids may cause elevation of the intracranial pressure. As tetracyclines may also cause elevation of the intracranial pressure, patients must not be treated with ATRA and tetracyclines at the same time
- Patient cannot take any other IMPs whilst on this protocol.
- Patients should not take Vitamin A supplements.
- Patients may not take warfarin or a coumarin preparation. LMWH should be used where possible.
- Chronic systemic corticosteroid use (≥10 mg of prednisone or an equivalent dose of other anti-inflammatory corticosteroids) for ≥28 days or use of other immunosuppressants.
- Patients may not receive any live vaccines starting from four weeks prior to receiving their first dose of study treatment until one month after receiving their last dose of ATRA and/or gemcitabine/nab-paclitaxel (whichever is last).

6.11.3 Medications to be used with caution

- The metabolism of paclitaxel is catalysed, in part, by cytochrome P450 isoenzymes CYP2C8 and CYP3A4. Therefore, in the absence of a PK drug-drug interaction study, caution should be exercised when administering paclitaxel concomitantly with medicines known to inhibit (e.g. ketoconazole and other imidazole antifungals, erythromycin, fluoxetine, gemfibrozil, cimetidine, ritonavir, saquinavir, indinavir, and nelfinavir) or induce (e.g. rifampicin, carbamazepine, phenytoin, efavirenz, nevirapine) either CYP2C8 or CYP3A4.
- Paclitaxel and gemcitabine do not share a common metabolic pathway. Paclitaxel clearance is primarily determined by CYP2C8 and CYP3A4 mediated metabolism followed by biliary excretion, while gemcitabine is inactivated by cytidine deaminase followed by urinary excretion. Pharmacokinetic interactions between paclitaxel and gemcitabine have not been evaluated in humans.
- As ATRA is metabolised by the hepatic P450 system, there is the potential for alteration of pharmacokinetics parameters in patients administered concomitant medications that are also inducers or inhibitors of this system. Medications that generally induce hepatic P450 enzymes include rifampicin, glucocorticoids, phenobarbital and pentobarbital. Medications that generally inhibit hepatic P450 enzymes include ketoconazole, cimetidine, erythromycin, verapamil, diltiazem and ciclosporin. There are no data to suggest that co-use with these medications increases or decreases either efficacy or toxicity of ATRA.
- During the first month of treatment with ATRA, there is a risk of thrombosis (both venous and arterial) which may involve any organ system. Therefore, caution should be exercised when treating patients with the combination of ATRA and anti-fibrinolytic agents, such as tranexamic acid, aminocaproic acid or aprotinin (refer to ATRA SmPC section 4.4).

6.12 Dose Modification and Delay

Toxicities are graded according to the NCI Common Terminology Criteria for Adverse Events (CTCAE), v4.03. Dose reductions are to be made according to the system showing the greatest degree of toxicity. Patients' safety will be monitored on an ongoing basis during this study. Dose increases are not permitted. If a patient experiences a clinically significant and/or unacceptable toxicity felt to be possibly related to ATRA or Gemcitabine and nab-paclitaxel, dosing may be interrupted at the investigators discretion for up to 28days (1 cycle), and supportive therapy administered as required. Given the non-overlapping toxicity profiles it is generally advised to interrupt/dose-reduce either ATRA or Gemcitabine or nab-paclitaxel, as deemed appropriate by the principal investigator. Listed below are recommendations to assist with toxicity management of ATRA and Gemcitabine and nab-paclitaxel.

6.12.1 General recommendations for dose reductions and delays

In the event that dose modifications are required at the beginning of a cycle due to haematological or non-haematological AEs, doses may be adjusted as per below.

If a patient experiences toxicities that are CTCAE ≥Grade 3 (see exceptions for primary endpoint) or clinically significant and/or experiences unacceptable toxicity not attributable to the disease or disease-related processes under investigation, ATRA/Gemcitabine and nab-paclitaxel dosing may be withheld for up to 28 days until toxicity improves to CTCAE Grade <2 or becomes clinically tolerable. Supportive therapy should be administered as required.

If toxicity improves to CTCAE Grade 2 within 28 days of ATRA/Gemcitabine and nab-paclitaxel dose interruption, reinstate ATRA/Gemcitabine and nab-paclitaxel at a reduced dose (1 dose level) maintaining treatment for toxicity as necessary (Table 5).

Where a CTCAE Grade >3 or clinically significant or intolerable toxicity does not improve to a lower CTCAE Grade within 28 days of ATRA/Gemcitabine and nab-paclitaxel dose interruption, ATRA/Gemcitabine and nab-paclitaxel should be permanently discontinued. In cases of nab-paclitaxel induced neuropathy, both ATRA and Gemcitabine can be continued.

Re-escalation is not permitted. Specific dose management for myelosuppression, elevation of liver enzymes, and pneumonitis are detailed below.

In the event that patients must have treatment delayed within a treatment cycle due to toxicities, those doses omitted during a cycle will not be made up.

Table 5: Dose modification levels for ATRA and Gemcitabine and nab-paclitaxel

**ATRA**			**Gemcitabine**			**Nab-Paclitaxel**		
Starting Dose	Reduction Levels		Starting Dose	Reduction Levels		Starting Dose	Reduction Levels	
mg/m^2	1	2	mg/m^2	1	2	mg/m^2	1	2
15	0	n/a	800	600	0	100	75	0
30	15	0	1000	800	600	125	100	75
45	30	15						

6.12.2 Dose modifications: Haematological AEs

Where possible dose modificiations for Gemcitabine / nab-Paclitaxel should follow the guidance as per the current version of the SmPc for nab-Paclitaxel, as detailed below:

Table 6: Gemcitabine/Nab-Paclitacel dose modifications for Haematological Adverse Events (AE)s. Please see Table 5 for dose modification levels.

**Cycle Day**	**ANC count (cells/mm^3)**		**Platelet count (cells/mm^3)**	**Nab-Paclitaxel Dose**	**Gemcitabine Dose**
**Day 1**	< 1500	OR	< 100,000	Delay doses until recovery	
**Day 8**	≥ 500 but < 1000	OR	≥ 50,000 but < 75,000	Reduce doses 1 dose level	
	< 500	OR	< 50,000	Withhold doses	
**Day 15: If Day 8 doses were given without modification:**					
**Day 15**	≥ 500 but < 1000	OR	≥ 50,000 but < 75,000	Treat with Day 8 dose level and follow with WBC Growth Factors<br>OR<br>Reduce doses 1 dose level from Day 8 doses	
	< 500	OR	< 50,000	Withhold doses	
**Day 15: If Day 8 doses were reduced:**					
**Day 15**	≥ 1000	AND	≥ 75,000	Return to the Day 1 dose levels and follow with WBC Growth Factors<br>OR<br>Treat with same doses as Day 8	
	≥ 500 but < 1000	OR	≥ 50,000 but < 75,000	Treat with Day 8 dose levels and follow with WBC Growth Factors<br>OR<br>Reduce doses 1 dose level from Day 8 doses	
	< 500	OR	< 50,000	Withhold doses	
**Day 15: IF Day 8 doses were withheld:**					
**Day 15**	≥ 1000	AND	≥ 75,000	Return to Day 1 dose levels and follow with WBC Growth Factors<br>OR<br>Reduce doses 1 dose level from Day 1 doses	
	≥ 500 but < 1000	OR	≥ 50,000 but < 75,000	Reduce 1 dose level and follow with WBC Growth Factors<br>OR	

				Reduce doses 2 dose levels from Day 1 doses
	< 500	OR	< 50,000	Withhold doses

6.12.3 Dose Modifications: Non-Haematological AEs

Where possible dose modificiations for Gemcitabine / nab-Paclitaxel should follow the guidance as per SmPc for nab-Paclitaxel, as detailed below:

Table 7: Dose modification of Gemctiabine/Nab-Paclitaxel for Non-Haematological Adverse Events (AE)s as per SmPC. Please see table 5 for dose modification levels.

**Adverse Event**	**Nab-Paclitaxel Dose**	**Gemcitabine Dose**
**Febrile Neutropenia**: Grade 3 or 4	Withhold doses until fever resolves and ANC ≥ 1500; resume at next lower dose level[a]	
**Peripheral Neuropathy**: Grade 3 or 4 *(see Table 8 and 9 for Grade 2)*	Withhold dose until improves to ≤ Grade 1; resume at next lower dose level	Treat with same dose
**Cutaneous Toxicity:** Grade 2 or 3	Reduce to next lower dose level discontinue treatment if adverse drug reaction persists	
**Gastrointestinal Toxicity:** Grade 3 mucositis or diarrhoea	Withhold doses until improves to ≤ Grade 1; resume at next lower dose level	

For any AEs which fall outside this range, the below guidance should be followed:**Please see table 5 for dose modification levels.**

Table 8: Dose modifications of Gemcitabine/ Nab-Paclitaxel for Non-Haem AEs not listed on SmPC on Day 1 of a cycle

**CTCAE Grade**	**Gemcitabine / Nab- Paclitaxel dose**
Grade 0-2 AE	Same as Day 1 previous cycle (except for Grade 2 peripheral neuropathy, and Grade 2 cutaneous toxicity: please refer to SmPC guidance as per Table 7)
Grade 2 peripheral neuropathy	Decrease nab-Paclitaxel to the next lowest dose and treat on time and no change to Gemcitabine administration
Grade 3 AE[1]	Decrease Gemcitabine and nab-Paclitaxel to next lower dose level
Grade 4 AE[2]	STOP and withdraw from protocol treatment[2]
Dose omitted in 2 previous consecutive cycles	Decrease dose to next lower dose level and continue throughout the rest of treatment

[1]If the toxicity only affects neuropathy, then only nab-Paclitaxel should be reduced.

[2]Pulmonary embolism (a Grade 4 toxicity in the CTCAE tables), if asymptomatic, will be exempt from this requirement.

If both day 8 and 15 treatments are withheld, commence subsequent cycle at next lower dose level

Table 9: Dose modifications of Gemcitabine/Nab-Paclitaxel for Non-Haem AEs not listed on SmPC within a Cycle

**CTCAE Grade**	**Gemcitabine / Nab- Paclitaxel dose**
Grade 0-2 AE, or Grade 3 nausea/vomiting and alopecia	Continue with same dose[1]; for nausea/vomiting, escalate anti-emetics as per local practice

Grade 2 peripheral neuropathy	Decrease doses of both drugs by 1 level, treat on time
Grade 3 AE (except as above)	Hold either or both drugs[1], dependent on investigator opinion regarding cause of toxicity, until resolution to < Grade 1. Then resume treatment at next lower dose level
Grade 4	Discontinue treatment[2]

[1]Except for peripheral neurotoxicity and cutaneous toxicity

[2] Pulmonary embolism (a Grade 4 toxicity in the CTCAE tables) if asymptomatic, will be exempt from this requirement.

6.12.4 Myelosuppression

ATRA is unlikely to cause myelosuppresion and does not require dose modification. Gemcitabine and nab-paclitaxel can cause myelosuppresion and dose modifications are required for both these drugs.

6.12.5 Elevation of Liver function tests (LFTs)

Hepatoxicity is more common with ATRA. However patients with stents should have stent bloackage ruled out by ultrasound and if present, it should be treated before declaring it AE. If a patient exhibits an aspartate aminotransferase (AST) or alanine aminotransferase (ALT) result in excess of 10 x ULN, or AST or ALT in excess of 8 x ULN in combination with a doubling of bilirubin from baseline, which is considered to be related to study drug, they will not be permitted to restart ATRA.

6.12.6 Pneumonitis

Pneumonitis is likely to be associated with nab-Paclitaxel and dose modifications are required for that drug. Before enrollment patients should be evaluated for familial, environmental or occupational exposure to opportunistic pathogens. Patients with a history of slowly progressive dyspnoea and unproductive cough or of conditions such as sarcoidosis, silicosis, idiopathic pulmonary fibrosis, pumonary hypersensitivity pneumonitis or multiple allergies will not be enrolled in the trial. During study treatment, episodes of transient or repeated dyspnoea with unproductive persistent cough or fever should be paid attention to. Radiographic evaluation with chest X-rays and CT scans (normal or high resolution) may be indicated to look for infiltrates, ground-glass opacities or honeycombing patterns. Pulse oximetry and pulmonary function tests can show respiratory and ventilation compromise.Infections should be ruled our with routine immunological / microbiological methods, Transbronchial lung biopsy is not recommended, given its limited value and risk of pheumothorax and haemorrhage, and should be reserved for cases with unclear aetiology. Nab-Paclitaxel administration should be interrupted upon a provisional diagnosis of interstitial pneumonitis and patients permanently discontinued from further nab-Paclitaxel treatment, if the diagnosis is confirmed. After rulling out an infectious aetiology, intravenous high-dose corticosteroid therapy should be instituted without delay, as per local guidelines.

6.12.6.1 Treatment Discontinuation

Treatment with the study drugs must be discontinued if it is considered to be in the best interest of the patient. Reasons for treatment discontinuation may include:

- Disease Progression
- Occurrence of intolerable side effects
- Intercurrent illness which prevents further treatment
- Patient choice

- Patient is pregnant
- Death
- Any alterations in the patient's condition which justifies the discontinuation of treatment in the investigator's opinion

Unless patients withdraw their consent, survival information and details of future anti-cancer treatments will continue to be collected as described in the table of assessments.

If the patient permanently discontinues study drug for a reason other than disease progression, such as toxicity, the state of disease in terms of progression should be noted at this time point. Ideally formal imaging should occur at this point to document the state of disease. Alternatively the previous scan should be used. Where possible scanning should continue as per normal practice until progression has been reached as per protocol. The date of progression of disease should be recorded in the CRF. Further treatment is at the treating doctor's discretion.

6.12.6.2 Management of Overdose

An overdose is defined as deliberate or accidental administration of study medication at a dose above that which is assigned to that individual patient. In the event of drug overdose, the principal investigator should be notified immediately and the patient observed closely for adverse effects. The patient should be treated symptomatically as appropriate and the incident of overdose and related AEs and/or treatment documented in the patient's medical record.

6.12.6.3 Precautions Regarding Contraception

ATRA causes serious birth defects when administered during pregnancy. There is a very high risk for any exposed foetus that a deformed infant will result if pregnancy occurs while taking ATRA, irrespective of the dose or duration of the treatment. Patients should use effective methods of contraception through defined periods during and after study treatment as specified below.

All patients with reproductive potential must agree to use a medically acceptable method of contraception throughout the treatment period and for 1 month after discontinuation of ATRA and / or Gemcitabine/nab-Paclitaxel (whichever is the latest) and for 6 months after discontinuation for male patients. Acceptable methods of contraception include IUD, oral contraceptive, sub-dermal implant and double barrier (condom with a contraceptive sponge or contraceptive pessary). Micro-dosed progesterone preparations ("mini-pill") are an inadequate method of contraception during treatment with ATRA. If patients are taking this pill they should be instructed to stop and another form of contraceptive should be prescribed instead.

Male patients will be given the opportunity to seek advice on cryopreservation of sperm prior to beginning study treatment because of the possibility of irreversible infertility due to therapy with gemcitabine and nab-paclitaxel.

If a patient, or the partner of a male patient, becomes pregnant during the male patient's participation in this study and during the 6 months after the last dose of Gemcitabine/nab-Paclitaxel, this must be reported to the Sponsor immediately. Where deemed appropriate after birth for instance in cases of congenital abnormalities or birth defects further follow up information will be collected. Time of follow-up will be decided on a case-by-case basis.

7 PHARMACOVIGILANCE

7.1 Definition of an AE

An AE is any untoward medical occurrence in a subject (including deterioration of a pre-existing medical condition) in a subject administered a medicinal product and which does not necessarily have a causal relationship with this treatment. An AE can therefore be any unfavourable and unintended sign (including an abnormal laboratory finding), symptom or disease temporarily associated with the use of an Investigational Medicinal Product (IMP), whether or not considered related to the IMP.

7.2 Recording of Adverse Events

AEs will be collected throughout the study, from informed consent until the safety visit; they will be followed up according to local practice until the event has stabilised or resolved, or the safety visit, whichever is the sooner. Any unresolved AEs at the patient's last visit, should be followed up for as long as medically indicated, but without further recording in the eCRF. The following details will be collected in the eCRF for each AE: AE term, date of onset, date of resolution, NCI-CTCAE grade maximum intensity, seriousness, Investigator causality rating against the study medication, action taken with regards to study medication and outcome.

7.3 Severity of Adverse Events

Severity is a measure of intensity whereas seriousness is defined by the criteria in section 7.6. Severity will be assessed using the grading scales found in the National Cancer Institute CTCAE version v 4.03 (June 14, 2010) for all AEs with an assigned NCI-CTCAE term. For those events without assigned NCI-CTCAE grades, the recommendation on page 1 of the NCI-CTCAE that converts mild, moderate and severe into NCI-CTCAE grades should be used. A copy of the NCI-CTCAE version 4.03 can be downloaded from the Cancer Therapy Evaluation Program website (http://ctep.cancer.gov).

7.4 Causality of Adverse Events

The Investigator will assess causal relationship between the IMPs and each AE.

7.5 Abnormal Laboratory Test Results

All clinically important abnormal laboratory test results occurring during the study will be recorded as AEs at the corresponding study visit. The clinically important abnormal laboratory tests will be repeated at appropriate intervals until they return either to baseline or to a level deemed acceptable by the investigator, and the Chief Investigators (CI), or until a diagnosis that explains them is made.

7.6 Serious Adverse Event (SAE) or Serious Adverse Reaction (SAR)

An SAE fulfils at least one of the following criteria:

- Is fatal – results in death (NOTE: death is an outcome, not an event)
- Is life-threatening[1]
- Requires inpatient hospitalisation or prolongation of existing hospitalisation[2]
- Results in persistent or significant disability/incapacity
- Is a congenital anomaly/birth defect
- Other important medical events[3]

7.7 Reporting of SAEs

Rapid reporting, within 24 hours of the PI or designee becoming aware of the event, of all SAEs occurring during the study or within 30 days following the completion of the study treatment by the subject, must be performed as detailed in the "SAE reporting instructions". If the investigator becomes aware of safety information that appears to be drug related, involving a subject who participated in the study, even after an individual subject has completed the study, this should also be reported to the Sponsor. All SAEs should be reported to the CECM STARPAC coordinating team using the SAE form and will be reviewed by the CI or designated representative to confirm relatedness and expectedness. Following documented assessment by the CI, the completed SAE form will be forwarded to Celgene and the Sponsor by the study team at CECM within the pre-specified timelines.

All SAEs must be reported to the CECM STARPAC coordinating team using the STARPAC SAE form via fax: +44(0)20 7882 8409 and within 24 hours of the site becoming aware of the event.

Please note all events should also be recorded in the relevant sections of the case report forms and patient medical records.

7.8 Non-Reportable Events

Disease progression or death as a result of disease progression will not be reported in an expedited manner in this study. However they will need to be reported on the relevant CRF.

In addition, due to the nature and stage of the disease in this study, the following situations that fulfil the definition of an SAE are excluded from recording/reporting on an SAE form however they should be recorded on the CRF and in the medical records.

- Elective hospitalisation and surgery for treatment of PDAC or its complications.
- Elective hospitalisation to make treatment or procedures easier.
- Elective hospitalisation for pre-existing conditions that have not been exacerbated by trial treatment

---

[1] *The term 'life threatening' in the definition of 'serious' refers to an event in which the patient was at risk of death at the time of the event; it does not refer to an event which hypothetically might have caused death if it were more severe.*

[2] *"Hospitalisation" means any unexpected admission to a hospital. It does not apply to scheduled admissions that were planned before study inclusion or visits to casualty (without admission). Elective admissions are also excluded.*

[3] *Medical judgement should be exercised in deciding whether an adverse event/reaction is serious in other situations. Important adverse events/reactions that are not immediately life-threatening, or do not result in death or hospitalisation but may jeopardise a subject, or may require intervention to prevent one of the other outcomes listed in the definition above should also be considered serious.*

Any of the following AEs that may fulfil the definition of an SAE should not be reported as an SAE but should be recorded in the medical notes and on the relevant eCRF. These are common features of pancreatic cancer and include:

- Lethargy
- Weight Loss
- Abdominal pain
- Weakness
- Nausea

However, if any of the above events are more intensive or excessive than expected as a feature of pancreatic cancer, as assessed by the Principal Investigator, then they will be reported as an SAE.Definition of an Adverse Reaction (AR)

An AR is any untoward and unintended response in a subject to an Investigational Medicinal Product (IMP) which is related to any dose administered to that subject. All adverse events judged by either the reporting investigator or the Sponsor as having a reasonable causal relationship to a medicinal product qualify as adverse reactions. The expression of reasonable causal relationship means to convey, in general, that there is evidence or argument to suggest a causal relationship.

7.9 Serious Adverse Reaction (SAR)

A SAR is an adverse reaction that is classed as serious and judged by either the reporting investigator or the Sponsor as having a reasonable causal relationship to a medicinal product.

7.10 Suspected Unexpected Serious Adverse Reaction (SUSAR)

The definition of a SUSAR is any SAR that is NOT consistent with the reference safety information as set out in section 4.8 of the current version of the ATRA , Gemcitabine or nab-Paclitaxel SmPC.

7.11 Reporting of SUSARs

Research sites will report SUSARs to the CECM STARPAC Coordinating team within 24-hours of the PI or co-investigator becoming aware of the event. Receipt of SUSAR by the CECM STARPAC Coordinating team is considered to be day 0 for CA reporting. The CECM coordinating team are responsible for rapid reporting of SAEs/SARs/SUSARs to the Sponsor. The Sponsor, in conjunction with the CI, is responsible for reporting UK-relevant SUSARs to the MHRA within the competent authority's reporting timelines.

It is the CI's responsibility to report SUSARs to the REC and to disseminate SUSARs to participating sites to inform all investigators involved in the trial that a SUSAR has been reported. The PIs are responsible for the follow-up of patients who have experienced a SUSAR, which should continue until recovery is complete or the condition has stabilised. Follow-up will be documented in the patient's notes.

7.12 Pregnancy

All pregnancies and outcomes of pregnancy should be reported to CECM by the investigator.

7.12.1 Maternal exposure

If a patient becomes pregnant while on study treatment all IMPs must be discontinued immediately. The Investigator must inform the CECM STARPAC Coordinating Office within 24 hours of becoming aware of the pregnancy using the Pregnancy eCRF.

Pregnancy itself is not regarded as an AE unless there is a suspicion that the IMPs may have interfered with the effectiveness of a contraceptive medication. Congenital abnormalities/birth defects and spontaneous miscarriages should be reported and handled as SAEs. Elective abortions without complications should not be handled as AEs. The outcome of all pregnancies (spontaneous miscarriage, elective termination, ectopic pregnancy, normal birth or congenital abnormality) should be followed up and documented even if the patient was discontinued from the study. The Investigator must provide the CECM STARPAC Coordinating Office with outcome information within 24 hours of becoming aware of the pregnancy outcome. Where deemed appropriate after birth for instance in cases of congenital abnormalities or birth defects further follow up information will be collected. Time of follow-up will be decided on a case-by-case basis.

7.12.2 Paternal exposure

Pregnancy of a male patient's partner is not considered to be an AE. Male patients may continue to receive IMP. However, the outcome of all pregnancies (spontaneous miscarriage, elective termination, ectopic pregnancy, normal birth or congenital abnormality), occurring from the date of the first dose until 6 months after discontinuing study treatment should, if possible, be followed up and documented even if the patient was discontinued from the study. The Investigator must provide the CECM STARPAC Coordinating Office with outcome information within 24 hours of becoming aware of the pregnancy outcome. Where deemed appropriate after birth for instance in cases of congenital abnormalities or birth defects further follow up information will be collected. Time of follow-up will be decided on a case-by-case basis.

7.13 Annual Reporting

Annual reports will be submitted to the main competent authority and the REC by the CECM STARPAC Coordinating team according to current requirements and copies will be forwarded to the Sponsor. The CI will carry out a risk benefit analysis of the IMPs encompassing all events having arisen on the trial. The Development Safety Update Report (DSUR) will be sent within the timelines set by the competent authorities. A copy of the annual report and any associated correspondence with the Competent Authority will also be sent to the Sponsor and to all participating sites.

The Annual Progress Report (APR) will be sent by the CI to the sponsor and REC, using the NRES template. The APR will be submitted on the anniversary date of the "favourable opinion" letter from the REC. A copy of the APR and an associated correspondence with REC will also be sent to participating sites.

7.14 Urgent Safety Measures

The CI may take urgent safety measures to ensure the safety and protection of the clinical trial subjects from any immediate hazard to their health and safety, in accordance with Regulation 30. The measures should be taken immediately. In this instance, the approval of the Licensing Authority Approval prior to implementing these safety measures is not required. However, it is the responsibility of the CI to inform the sponsor, Research Ethics Committee (REC) and the MHRA (via telephone for discussion with the medical assessor at the clinical trials unit) of this event immediately. The CI has an obligation to inform both the Competent

Authority and REC in writing within **3 days**, in the form of a substantial amendment. The sponsor must be sent a copy of the correspondence with regards to this matter.

8 STATISTICAL CONSIDERATIONS

8.1 Definition of study endpoints

To meet the objectives of this study, data on the following endpoints will be collected:

- Dose limiting toxicities
- Serum vitamin A levels
- ATRA PK parameters
- Investigator assessment of patient disease status
- Exploratory biomarkers
- Pharmacodynamic biomarkers

Derivations, calculations and analysis plans for each of these endpoints are presented in the sections below.

8.2 Determination of sample size

Part 1: The number of patients that will be enrolled in the first part of the study will be dependent on the number of dose levels required to ultimately determine the MTD (Please refer to section 1.5.3 for details of dose levels.). As a result, the sample size can only be estimated prior to carrying out the study based on several assumptions. It is expected that a maximum of of 24 patients will be enrolled into Part 1of the study.

MTD will be defined as the highest dose level of ATRA and Gemcitabine / Nab-Paclitaxel for which the probability of a DLT is closest to 30%.

Part 2: A maximum of 10 patients will be recruited in the second part of the study. OBD will be defined as the lowest dose level of ATRA for which a post cycle serum Vitamin A level between 1.5 and 2.5 µM can be achieved. As STARPAC is the first study to explore this combination of treatments on these patients, a sample size of 10 has been considered reasonable to provide pilot data on optimal biological dose (OBD).

8.3 Populations for Analyses

Table 10: Populations for analysis

Endpoints	Population
DLT	All patients in part 1 of the study who completed the first 28 days of treatment without dose modifications (delays, dose reductions) or who experienced dose-limiting toxicities (DLT) during the first 28 days of treatment
OBD	All patients in part 2 of the study who completed the first 28 days of treatment without dose modifications (delays, dose reductions) or who experienced dose-limiting toxicities (DLT) during the first 28 days of treatment.
Disease Control Rate	All patients who meet the eligibility criteria and who completed the first 2 cycles of treatment or progressed within the first 2 cycles of treatment. Disease control is defined as complete or partial response or stable disease maintained ≥8 weeks (as assessed by the site radiologist and/or investigator, using RECIST 1.1).
ORR	All patients who meet the eligibility criteria and who completed the first 2 cycles of treatment or progressed within the first 2 cycles of treatment.
AEs	All patients who received at least one administration of the investigational medicinal product. Safety data will be reported separately for patients within the two separate parts of this phase 1B study.
PK	PK analysis will include all patients with at least one full set of pre- and post-treatment samples.
PFS	All patients in the study who meet the eligibility criteria and who completed the first 2 cycles of treatment or progressed within the first 2 cycles of treatment.
OS	All patients in the study who meet the eligibility criteria and who received study medication drug.

8.4 Endpoints

8.4.1 Primary endpoint

Part 1: The primary endpoint of this part of the study is the occurrence of any of the following toxicities, during the first 28 days of treatment as assessed by Adverse Events (AEs) (CTCAE, v4.03) and which can be attributed as <u>possibly, probably or definitely related to the study treatment</u>.

<u>Haematological DLTs</u>:

- Grade 4 neutropenia > 7 days duration (*see note).
- Febrile neutropenia (fever of unknown origin without clinically or microbiologically documented infection) with Grade 3 or 4 neutropenia (absolute neutrophil count [ANC] <1.0 x 10^9/L and fever >38.5ºC)
- Grade 4 anaemia related to chemotherapy which requires transfusion therapy
- Grade 4 thrombocytopenia: (a) for >7 days or more (*see note), or (b) associated with active bleeding, or (c) which requires transfusion therapy

*Note: In the event of a Grade 4 neutropenia or Grade 4 thrombocytopenia, a full blood count must be performed at least on day 3 and 7 after the onset of the event to determine if a DLT has occurred and continue to monitor the patient closely until resolution to Grade 3 or less. If resolved within 8 days such a Grade 4 event, it will not be considered DLT. Leukopenia or lymphopenia in the absence of neutropenia is NOT considered dose limiting.

Non-hematologic DLT is defined as any grade 3 or 4 non-haematologic toxicity lasting for more than 5 days that occurs during the first cycle. The specific exemptions are:

- Grade 3 or 4 anorexia, nausea, vomiting or diarrhoea. However, Grade 3 or 4 anorexia, nausea, vomiting or diarrhoea despite optimal treatment with anti-emetics or anti-diarrheal medication, as appropriate, for five days will qualify as a DLT.
- Grade 3 electrolyte abnormalities
- Grade 3 biochemical AEs

The MTD will be calculated as the highest dose with an estimated probability of a DLT of less than 30%. The 5 dose combinations specified in section 6.8.1 will be used to estimate the MTD with a DLT rate of 30% by a 2 stage process with the first stage a rule-based escalation from which on occurrence of the first DLT there is a switch to the second stage, use of the continual reassessment method (CRM, O'Quigley, 2000). The CRM method estimates toxicity at each dose using Bayesian methodology to update a dose-toxicity curve. This is known to generally have a superior operating performance compared to the traditional 3+3 approach as it bases estimation on all prior dose data rather than just the last 3 patients usually resulting in more accurate estimation of the MTD, fewer toxicities and more patients treated at assumed efficacious doses close to the MTD (Iasonos 2008). This has resulted in its increasing use in phase 1 trials including cancer (Iasonos 2014). A modified CRM will be implemented via the R package "bcrm" (Sweeting 2013) with prior toxicity estimates of 10%, 15%, 20%. 25% and 30%, using a hyperbolic tangent form of the dose-toxicity curve, restricting dose escalations to a single level and we shall terminate the phase at 24 patients or when a dose level is selected 6 times at which point the current dose estimate is declared the MTD.

The 2 stage type of design with an initial rule based escalation and a switch to CRM on occurrence of a DTL is discussed elsewhere (Iasonos, 2012).

Stage 1: The first three patients will be recruited at dose level D2 unless a DLT occurs in which case the next patient will be recruited in stage 2 using a modifled CRM. If no DLTs occur the 4th and, until a DLT occurs, subsequent patients will be recruited at the same dose (as the previous patient aka current dose) if no previous patient on current dose has passed 2 weeks DLT-free follow up, otherwise if a patient on the current dose has already passed 2 weeks DLT-free follow up then the next patient will be recruited at one dose higher (unless current dose is D5). The initial rapid escalation (i.e. starting at D2 not D1 and escalating on 2 weeks DLT-free follow-up) is taking place because Gemcitabine and Nab-Paclitaxel are given at established doses and ATRA is thought to be non-toxic. This rapid escalation is balanced by the first 3 patients being recruited at D2 (or possibly lower in the event of a DLT). On the recruitment of the 24th patient or if 6 patients are recruited at D5 the trial will stop (NO GO to stage 2) and the MTD will be estimated based on a CRM of the recruited patients.

Stage 2: The study will progress to this stage if a DLT occurs in stage 1 and stage 1 did not have 24 patients or 6 patients are recruited at D5. For the next patient CRM will be used for dose assignment using (1) all patients with DLTs known by the next patients recruitment time plus (2) all patients who have survived 3

weeks DLT-free plus. Escalation by more than 1 level will not be not allowed. If the number of patients on the current dose is 6 or we recruit 24 patients in total then the trial will stop and the MTD will be estimated based on a CRM of the recruited patients.

The properties of the 2 stage dose escalation + modified CRM procedure were assessed using the R programming language. Hypothetical data was simulated with patients recruited randomly, with average arrival 1 a week, and 3 scenarios of simulated toxicity rates applied where toxicity rates at the 5 doses are lower, higher or equal (aka medium) to the prior estimates used in the CRM analysis. The DLT times, for those simulated to have DLTs using the specified toxicity rates for each dose level, were simulated as occurring uniformly between 1 and 3 weeks after recruitment date (note patients with 3 weeks DLT-free followup are treated as not having a DLT on the assumption that DLTs occur within 3 weeks – in the simulations, but not in the actual study, none of these patients would have a later DLT after 3 weeks).

2000 datasets were simulated for each toxicity scenario and the table below compares the performance in each circumstance in terms of total number of patients recruited (over 2000 simulated studies) with average number per study, the total and average number of DLTs, the proportion of times each dose level is selected as MTD and the proportion of total patients (over 2000 studies) treated at each dose.

**True DLT rates**	**Prior use**	**Total patients (average / simulation) Total DLTs (average / simulation)**	**MTDs selected (percent)**	**Patients at each dose (percent)**
High<br>D1:10%<br>D2:20%<br>D3:25%<br>**D4:30%**<br>D5:40%	D1:10%<br>D2:15%<br>D3:20%<br>D4:25%<br>D5:30%	24,561 (12.3)<br><br>5726 (2.86)	D1: 244 (12%)<br>D2: 440 (22%)<br>D3: 534 (27%)<br>**D4: 322 (16%)**<br>D5: 460 (23%)	D1: 3912 (16%)<br>D2: 8699 (35%)<br>D3: 5399 (22%)<br>**D4: 3558 (14%)**<br>D5: 2993 (12%)
Medium<br>D1:10%<br>D2:15%<br>D3:20%<br>D4:25%<br>**D5:30%**	D1:10%<br>D2:15%<br>D3:20%<br>D4:25%<br>D5:30%	26,238 (13.1)<br><br>5213 (2.61)	D1: 137 (7%)<br>D2: 286 (14%)<br>D3: 470 (24%)<br>D4: 327 (16%)<br>**D5: 780 (39%)**	D1: 2657 (10%)<br>D2: 8471 (32%)<br>D3: 5620 (21%)<br>D4: 4487 (17%)<br>**D5: 5003 (19%)**
Low<br>D1:5%<br>D2:10%<br>D3:12%<br>D4:15%<br>**D5:20%**	D1:10%<br>D2:15%<br>D3:20%<br>D4:25%<br>D5:30%	30,268 (15.1)<br><br>4150 (2.08)	D1: 47 (2%)<br>D2: 131 (7%)<br>D3: 305 (15%)<br>D4: 192 (10%)<br>**D5: 1325 (66%)**	D1: 1544 (5%)<br>D2: 8040 (27%)<br>D3: 5687 (19%)<br>D4: 5302 (18%)<br>**D5: 9695 (32%)**

Part 2: The primary endpoint of this part of the trial is the determination of OBD based on serum Vitamin A levels measured at the end of each treatment cycle. The OBD will be estimated for patients recruited at the MTD as determined by Part 1 and at ATRA doses higher and lower to ensure that the OBD can be identified. The OBD will obtain a post cycle serum Vitamin A at end of first cycle level of 2μM. Each patient has 4 cycles and for subsequent cycles intra-patient dose de-escalation or escalation for ATRA dependent on the vitamin levels at end of each previous cycle being above 2.5μM or below 1.5 μ M. Please see section 1.5.3 for dosing levels.The range of ATRA levels can be expanded based on assessing accumulating part 1 and part 2 Vitamin

A levels. The main interest will be in the levels of final cycle ATRA dosing and in the heterogeneity of the final applied dose. Regression to the mean will be assessed.

8.4.2 Secondary endpoints

8.4.2.1 PK Paramaters

Single dose ATRA PK parameters including T_{max}, C_{max}, and AUC will be determined in samples collected Cycles 1, 2 and at 0-30 minutes before ingesting ATRA and at 30, 1hr, 2hrs, 3hrs, 4hrs and 5hrs post ATRA administration. PK parameters will be derived using non-compartmental analysis for ATRA and in Part 1 of the study may include single dose and multiple dose tmax, Cmax, AUC(0-t), AUC, and dose normalised Cmax, AUC and AUC(0-t).

If the ATRA PK data is highly variable, he assessment of the impact on ATRA PK on exposure to Gemcitabine and nab-paclitaxel by co-administration will be made by comparing the Gemcitabine and nab-paclitaxel PK data in combination with ATRA in this study with historic data from previous Gemcitabine and nab-paclitaxel studies when dosed alone (this would be an exploratory endpoint).

8.4.2.2 Change in serum Vitamin A levels

Serum Vitamin A levels will be measured in samples collected at the end of Cycles 1 and 2. Correlation with the 5 dose levels of the change in serum Vitamin A levels relative to baseline at the end of cycles 1 and 2 will be assessed by a Spearman rank correlation.

8.4.2.3 Objective response rate

ORR is defined as the percentage of patients measurable disease at baseline who have at least one visit response of CR or PR prior to any evidence of progression (as defined by the site radiologist using CT scans (RECIST v1.1)). The denominator for ORR will be all patients at risk. The ORR will be assesed as a comparison of proportions between the 5 dose levels using a Fisher's Exact test. Patients without a post-baseline tumour assessment will be considered to be non-responders.

8.4.2.4 Progression-Free Survival

PFS is defined as the the time from the date of registration to the date of first documented tumour progression (as assessed by the site radiologist and/or investigator, using RECIST v1.1) or death from any cause, whichever occurs first. The effect of dose on progression survival will be assessed by log-rank test. For patients who have not died or experienced disease progression at the end of study, PFS will be censored onat 12 month follow up or the last date the patient was known to be progression free, which ever is the earliest.

8.4.2.5 Overall Survival

Overall survival is defined as the time from registration to death from any cause or 12 months follow up whichever occurs first. All deaths will be included, whether they occur on study or following treatment discontinuation. For patients who have not died, OS will be censored at the date of last contact or 12 month follow up which ever is the earliest. Analysis methods are the same as those described for PFS.

8.4.2.6 Safety outcome measures

- Incidence of serious adverse events
- Incidence of grade 3 and 4 adverse events (CTCAE, version 4.03)
- Incidence of all adverse events of all grades
- Adverse events leading to discontinuation of the study medication

- Clinically significant changes in vital signs and clinical laboratory results during and following study drug administration

8.4.3 Safety Parameters

Safety will be assessed through summaries of adverse events, laboratory test results, and changes in vital signs. Safety analyses will include all patients who receive any amount of study treatment.

The adverse events occurring during cycle 1 in patients within Part 1 of the study will be used to define the MTD. Dose-limiting toxicities (DLT) are defined as described in section 10.1.1.1. Safety data will be reported separately for patients in Part 1 and for patients treated at the recommended Part 2 dose (regardless of whether they are within Part 1 or Part 2). The worst toxicity during each cycle and the worst toxicity during the entire treatment will be determined separately for each patient according to the criteria specified above.

Vital signs, temperature, weight and ECOG performance status data will be listed or summarized by study site, patient ID number, and scheduled measurement time. Laboratory data will be tabulated with values outside normal ranges identified and summarized by NCI CTCAE (Version 4.0) grade.

Verbatim descriptions of treatment-emergent adverse events will be mapped to the appropriate thesaurus terms and summarized by mapped term, appropriate thesaurus level, and NCI CTCAE grade. For each patient's adverse event, the maximum severity reported will be used in the summaries. Serious adverse events, including deaths, will be summarized separately.

8.5 End of Study Definition

The 'end of study' is defined as the last patient's last follow-up visit (LPLV). It is the responsibility of the Sponsor to inform the MHRA and REC within 90 days of the 'end of trial' that the study has closed. In cases of early termination of the trial (e.g. due to toxicity) or a temporary halt, the coordinating centre will notify the MHRA and REC within 15 days of the decision and a detailed, written explanation for the termination/halt will be given.

8.6 Statistical presentation

Data will be presented in a descriptive fashion. Variables will be analysed to determine whether the criteria for the study conduct are met. This will include a description of patients who did not meet all the eligibility criteria, an assessment of protocol violations, study drug accountability and other data that impact on the general conduct of the study.

Baseline characteristics will be summarised for all patients in Part 1 and separately for patients in Part 2. Patients who died or withdrew before treatment started or do not complete the required safety observations will be described and evaluated separately. Treatment administration will be described for all cycles. Dose administration, dose modifications or delays and the duration of therapy will be described.

All patients will be assessed for toxicity. Safety variables will be summarised by descriptive statistics. AEs will be reported for each dose level, summarised by incidence rates and classified by the worst observed severity grade, and also for the recommended part 2 dose level (including part 1 and 2 data). Laboratory data will be presented by dose level at each observation time. Values outside normal limits will be identified and

summarised by frequency distribution. Laboratory variables will be described using the NCI CTC (Version 3.0).

Individual patient data will be presented for each of the pharmacokinetic parameters. When appropriate for the number of patients at a given dose level, data will also be summarised and presented by dose level. The pharmacodynamic data will be summarised by descriptive statistics.

Documenting anti-tumour activity is a secondary objective in this trial. Patients must receive at least two cycles to be evaluable for response. Disease control rate, and objective response rate will be calculated separately for dose expansion cohort.

9 DATA HANDLING AND RECORD KEEPING

9.1 Confidentiality

All information which is generated in the trial will be kept strictly confidential. The researchers conducting the trial will abide by the Data Protection Act 1998, and the rights the patient has under this act.

Once a patient consents to participate in the trial, his GP will be informed of their involvement in the trial as long as the patient consents that his GP can be informed. Parts of the patients' medical records and the data collected for the trial will be looked at by authorised personnel from the sponsor of this trial Barts Health NHS Trust. It may also be looked at by representatives of regulatory authorities and other authorised personnel from the patient's NHS Trust, to check that the trial is being carried out correctly. This is clearly stated on the consent form.

All of the above bodies have a duty of confidentiality to the patient as a research participant and nothing that could reveal their identity will be disclosed outside the research site. All data will be stored in a locked and dedicated room only accessed by authorised personnel.

9.2 Study Documents

All trial related documents should be filed in the Trial Master File (TMF), the Investigator's Site File (ISF) and the Pharmacy Site File (PSF). The ISF and PSF should contain essential documents as per the contents page provided to the Investigator by the STARPAC Coordinating team.

The Centre for Experimental Cancer Medicine will inform the PI, and their staff, of any regulatory updates and forward on any relevant documentation. It is the participating PI's responsibility to maintain the ISF and PSF and keep all records up to date.

9.3 Case Report Forms (CRFs)

This trial uses electronic case report forms (eCRFs). Sites will receive training for appropriate CRF completion. CRFs will be submitted electronically to the STARPAC Coordinating team within the Centre for Experimental Cancer Medicine (CECM), Barts Cancer Institute, Queen Mary University London. and should be handled in accordance with the CECM's SOPs. Any data queries arising from initial review will be sent to the relevant centre for resolution.

All CRFs should be completed by designated, trained examining personnel or the study coordinator as appropriate. The CRF should be reviewed and electronically signed and dated by the principal investigator. In addition, at the end of the study, the principal investigator will receive patient data for his or her site in a readable format on a compact disc that must be kept with the study records. Originals are retained at site until end of the study to ensure that all Source Data Value (SDV) is completed (this prevents generation or multiple 'original' documents') and then at the end of the study, the site a provided with paper copies and originals are taken by CECM.

The Trial Management Group reserves the right to amend or add to the CRFs as appropriate. Revised or additional forms should be used by centres in accordance with the guidelines provided by the sponsor.

CECM will be responsible for monitoring transfer and receipt of biological specimens. Tracking forms will be sent by centres to CECM to monitor the transfer of all biological samples. All data will be handled, computerised and stored in accordance with the Data Protection Act 1998.

9.4 Record Retention and Archiving

At the end of the trial all documentation, as defined by GCP, should be stored by each individual site's archiving facility, until written notification, for destruction, from the Sponsor. The location of the archiving facility must be provided to the Centre for Experimental Cancer Medicine.

In addition, source documentation (medical notes, images, results etc) should be retained, as per local policy, for the duration of the archiving period.

These will be stored for a minimum of 20 years or the maximum period required by the country and Institution in which the trial will be conducted, whichever is longer. The Centre for Experimental Cancer Medicine should be contacted prior to destruction.

The Centre for Experimental Cancer Medicine will arrange a 'close out' visit where all trial documentation will be prepared for archiving by that site. Records will be retained at each individual site. All records relating to the trial should be stored together, including the ISF, PSF and CRFs; patient case report forms (CRFs). It is the responsibility of the Principal Investigator to ensure a full set of records is collated and documented.

9.5 Compliance

This trial will be conducted in accordance with the principles of Good Clinical Practice (GCP) as laid out in the EU directive and The Medicines for Human Use (Clinical Trials) Regulation 2004, and its amendments.

In addition, sponsor auditors and Competent Authority inspectors will be allowed access to CRFs, source documents and other trial files to evaluate the trial.

10 STUDY MANAGEMENT

The reporting relationships between the committees will be described in the relevant committee charters.

10.1 Trial Management Group (TMG)

The TMG will consist of members of the CECM coordinating centre (CI, Trial Chairman, Trial Coordinators, Project Manager, Senior Research Pharmacist and Statistician). The role of the TMG will be to monitor all

aspects of the conduct and progress of the trial, ensure that the protocol is adhered to and take appropriate action to safeguard participants and the quality of the trial itself. The TMG will meet at least twice a year.

10.2 Safety Review Committee

The Safety Review Committee (SRC) will consist of the Chief Investigator, Trial Chairman, the Trial Coordinator, Project Manager, study statistician and local investigators or delegates from each actively recruiting site. A Celgene Representative may also be invited to attend. The committee will evaluate safety data acquired during the 1Bi part of the study and make recommendations on dose escalation / modification decisions in the 1Bi and confirm the dose to take forward to the 1Bii part of the study. The frequency of SRC meetings will be based on the occurrence of DLTs recorded during the trial, but as a minimum, will be held every fortnight. For the 1Bii part of the study the SRC will meet continue to meet to review safety data on a monthly basis.

11 CLINICAL GOVERNANCE ISSUES

11.1 Ethical Considerations

The trial will be conducted in accordance with ethical principles founded in the Declaration of Helsinki. A Research Ethics Committee (REC) will review all appropriate trial documentation in order to safeguard the rights, safety and well-being of patients.

The trial will only be conducted at sites where appropriate approval has been obtained.

The Centre for Experimental Cancer Medicine will inform the REC of any changes to the conduct of the trial and seek approval for these changes and any amended patient materials. The Chief investigator will maintain an accurate and complete record of all written correspondence to and from the REC and will agree to share all such documents and reports with the sponsor.

The informed consent and any other documentation provided to subjects will be revised if important new information becomes available that is relevant to the subject’s consent. Amended documents will be approved by the REC before distribution to patients.

11.2 Summary Monitoring Plan

Monitoring for the STARPAC study will consist of a combination of activities performed by the study team, and trial committee members. This includes review of data and progress of the trial by trial oversight committees, daily monitoring at CECM, central monitoring and on-site monitoring. Refer to STARPAC Monitoring Plan for further details.

Monitoring will involve regular on site reviews of the Investigator Site File (ISF) and Pharmacy Site File (PSF) as well as a proportion of Source Data Verification (SDV). This will involve direct access to patient notes at the participating hospital sites which will include the review of consent forms and other relevant investigational reports. Missing data will be sought, unless confirmed as not available.

During these visits the sites activity will be monitored to verify that:

- Source data transcribed onto eCRFs is authentic accurate and complete
- Safety, rights and well-being of the participants are being protected
- The study is being conducted in accordance with the currently approved protocol
- Any other study agreements, GCP and all applicable regulatory requirements are met

11.3 Audit and Inspection

This study may be audited by representatives from the coordinating centre and sponsor. The investigator and institution will be informed of the audit outcome. Investigators are obliged to cooperate in any audit allowing the auditor direct access to all relevant documents and allocate his/her time and the time of his/her staff to the auditor to discuss any findings or issues. Audit may occur at any time during or after completion of the study.

Inspections may be carried out by the Competent Authority at any time and the investigator should notify the sponsor immediately if there are any such plans for an inspection.

11.4 Reporting of Serious Breaches in GCP or the Trial Protocol

All investigators participating in the trial will promptly notify the Chief Investigator or Sponsor of a serious breach (as defined in Regulation 29A of the Medicines for Human Use (Clinical Trials) Regulations 2004 [Statutory Instrument 2004/1031], as amended by Statutory Instrument 2006/1928) that they become aware of. The CI is responsible for notifying the sponsor within 24 hours of becoming aware of a serious breach.

The Sponsor is responsible for notifying the licensing authority in writing of any serious breach of:

- The conditions and principles of GCP in connection with that trial; or
- The protocol relating to that trial, as amended from time to time in accordance with regulations 22 to 25, within 7 days of becoming aware of that breach.

A “serious breach” is a breach which is likely to effect to a significant degree:

- The safety or physical or mental integrity of the subjects of the trial; or
- The scientific value of the trial.

Participating centres should contact the Centre for Experimental Cancer Medicine or CI for further information.

12 STUDY FINANCES

12.1 Funding Sources

This trial is investigator designed and led. It is funded by the Medical research Council and Celgene Sarl Inc.

12.2 Patient expenses / payments

There are no participant study payments in this study and travel expenses will only be covered for study visits related to the imaging aspect (DW_MRI) for this study.

13 SPONSORSHIP AND INDEMNITY

Dr David Propper, Barts Health NHS Trust is the Chief Investigator.

Prof Hemant Kocher, Queen Mary University of London is the Trial Chairman.

Barts Health NHS Trust is sponsoring the study.

14 PUBLICATION POLICY

This is an investigator-led study sponsored by the CI’s substantive employer, Barts Health NHS Trust. The data collected will not be used to licence/register any pharmaceuticals. Authorship of the final manuscript(s), interim publications, or abstracts will be decided according to active participation in the statistical design and

analysis, Trial Management Group and accrual of eligible patients by the individual principal investigators and the contributing centres. The correct designation for this site is ‘Centre for Experimental Cancer Medicine, Barts Cancer Institute, Queen Mary University of London’. No participant may present data from his/her centre separately from the rest of the study results unless approved by the Trial Management Group and the sponsor. The sponsor will be notified of any outputs of the research such as guidelines, publications, presentation, changes in service delivery etc. prior to external submission or presentation.

In the event that research misconduct or data integrity concerns have been raised, the JRMO, as sponsor, with senior management of the affected organisation, reserves the right to review, request a hold on publication submission or to refuse permission to publish in discussion.

Responsibility for ensuring accuracy of any publication from this study is delegated to the Chief Investigator and Trial Chairman.

The full study report will be accessible via Eudra CT and Clinical trials.gov as applicable.

15 APPENDIX 1: RECIST Criteria (Version 1.1)

1.1 Introduction

This appendix details the implementation of RECIST 1.1 Guidelines (Eisenhauer et al 2009) with regards to Investigator assessment of tumour burden including protocol-specific requirements for this study.

1.2 Definition of measurable, non-measurable, target and non-target lesions

1.2.1 Measurable

A lesion, not previously irradiated, that can be accurately measured at baseline as ≥10 mm in the longest diameter (except lymph nodes which must have short axis ≥15 mm) with CT or MRI with slice thickness no greater than 5 mm, and which is suitable for accurate repeated measurements.

1.2.2 Non-measurable

- All other lesions, including small lesions (longest diameter <10 mm or pathological lymph nodes with 10 to <15 mm short axis at baseline*); Lymph nodes with <10 mm short axis are considered non-pathological and should not be recorded or followed as NTL.
- Truly non-measurable lesions include the following: leptomeningeal disease, ascites, pleural / pericardial effusion, inflammatory breast disease, lymphangitic involvement of skin or lung, abdominal masses/abdominal organomegaly identified by physical examination that is not measurable by reproducible imaging techniques.
- Previously irradiated lesions[1]
- Skin lesions assessed by clinical examination [2]
- Brain metastasis [2]

1.2.3 Special Cases

- <u>Lymph nodes</u>: lymph nodes are measured in the short axis (perpendicular to longest diameter) as this is more reproducible and predictive of malignancy. Lymph nodes with <10 mm short axis are considered non-pathological and should not be recorded or followed as NTL. Lymph nodes with a short axis ≥15 mm are considered measureable. Lymph nodes with short axis of 10 to <15 mm are considered pathological but non-measurable and should not be recorded or followed as NTL.
- <u>Bone lesions</u>: Lytic bone lesions or mixed lytic–blastic lesions, with identifiable soft tissue components, can be considered measurable if the soft tissue component meets the definition of measurability. Blastic lesions are considered non-measurable.
- <u>Cystic lesions</u>: Lesions that meet radiographic criteria for simple cysts should not be considered as malignant lesions (neither measurable nor non-measurable). Radiographically indeterminate, complex "cystic" lesions should be considered non-measurable lesions. Cystic metastases thought to be cystic metastases can be considered measurable lesions if they meet the criteria for measurability from a

[1] Localised post-radiation changes which affect lesion sizes may occur. Therefore, lesions that have been previously irradiated will not be considered measurable and must be selected as Non-Target Lesions (NTL) at baseline and followed up as part of the NTL assessment.

[2] Skin lesions assessed by clinical examination and brain lesions are considered as NTL.

radiological point of view, but if non-cystic lesions are present in the same patient these should be selected as target lesions.

1.2.4 Target lesions

A maximum of 5 measurable lesions (with a maximum of 2 lesions per organ), representative of all lesions involved suitable for accurate repeated measurement, should be identified as target lesions (TL) at baseline.

1.2.5 Non-Target lesions

All other lesions (or sites of disease) not recorded as TL should be identified as NTL at baseline.

1.3 Methods of assessment

The same method of assessment and the same technique should be used to characterize each identified and recorded lesion at baseline and during follow-up visits. A summary of the methods to be used for RECIST assessment is provided below and those excluded from tumour assessments for this study are highlighted, with the rationale.

Target Lesions	Non-Target Lesions	New Lesions
CT (preferred)<br>MRI	CT (preferred)<br>MRI<br>Clinical examination<br>X-ray, Chest x-ray	CT (preferred)<br>MRI<br>Clinical examination<br>X-ray, Chest x-ray<br>Ultrasound

Table 1: Summary of Methods of Assessment

1.3.1 CT and MRI

CT and MRI are generally considered to be the best currently available and reproducible methods to measure TL selected for response assessment and to assess NTL and identification of any new lesions.

In the STARPAC study it is recommended that CT examinations of the chest and abdomen will be used to assess tumour burden at baseline and follow-up visits. CT examination with intravenous (i.v.) contrast media administration is the preferred method.

MRI should be used where CT is not feasible or it is medically contra-indicated. For brain lesion assessment, MRI is the preferred method.

1.3.2 Clinical examination

In the STARPAC study, clinical examination will not be used for assessment of TL. Clinically detected lesions can be selected as target lesions if they are assessed by CT or MRI scans. Clinical examination can be used to assess NTL and to identify the presence of new lesions.

1.3.3 X-ray

In the STARPAC study, chest x-ray assessment will not be used for assessment of TL as they will be assessed by CT examination or MRI examination. Chest X-ray can, however, be used to assess NTL and to identify the presence of new lesions.

In the STARPAC study plain x-ray may be used as a method of assessment for bone NTL and to identify the presence of new bone lesions.

1.3.4 Ultrasound

In the STARPAC study, ultrasound examination will not be used for assessment of TL and NTL as it is not a reproducible method, does not provide an accurate assessment of tumour size and it is subjective and operator dependent. Ultrasound examination can, however, be used to identify the presence of new lesions. If new clinical symptoms occur and an ultrasound is performed then new lesions should be confirmed by CT or MRI examination.

1.3.5 Endoscopy and laparoscopy

In the STARPAC study, endoscopy and laparoscopy will not be used for tumour assessments as they are not validated in the context of tumour assessment.

1.3.6 Tumour markers

In the STARPAC study tumour markers will not be used for tumour response assessments as per RECIST 1.1.

1.3.7 Cytology and histology

In the STARPAC study histology will not be used as part of the tumour response assessment as per RECIST 1.1.

Cytological confirmation of the neoplastic origin of any effusion that appears or worsens during treatment is required when the measurable tumour has met criteria for response or stable disease. In such circumstances, the cytology is necessary to differentiate between response / stable disease (an effusion may be a side effect of the treatment) and progressive disease (if the neoplastic origin of the fluid is confirmed). Where cytology findings are not available, any effusion that significantly worsens (from trace to large) or appearance of clinically significant effusion (requiring change in drug therapy) during the study treatment will be considered to be progression of NTL, or disease progression due to new lesions.

1.3.8 Isotopic bone scan

In the STARPAC study isotopic bone scans will not be used for tumour response assessments as per RECIST 1.1.

1.3.9 FDG-PET scan

In the STARPAC study FDG-PET scans will not be used for tumour response assessments as per RECIST 1.1.

1.4 Tumour response evaluation

1.4.1 Schedule of evaluation

Baseline/screening assessments will be performed using contrast-enhanced CT scans of the chest, abdomen and pelvis, with additional anatomy as clinically indicated by extent of disease[1]. Subsequent tumour assessments should include CT scans of the chest abdomen, pelvis and other sites of disease.

MRI scans may be substituted for CT scans but MRI of the chest may only be performed with approval from the Sponsor. Additional anatomy may be imaged at follow-up on suspicion of new lesions. A documented standard-of-care tumour assessment performed within 28 days before Day 1 of Cycle 1 may be used for the screening assessment provided it meets the above requirements. The same imaging method used at screening must be used throughout the study.

Baseline assessments should be performed no more than 28 days before the start of study treatment and ideally should be performed as close as possible to the start of study treatment. The same radiographic procedure used to define measurable disease sites at screening must be used throughout the study (e.g., the same contrast protocol for CT scans).

Tumour assessments will be performed at screening and every 6 weeks (±7 days) thereafter until objective disease progression as defined by RECIST 1.1. If a patient discontinues treatment prior to progression then the patient should still continue to be followed until objective disease progression as defined by RECIST 1.1.If an unscheduled assessment was performed and the patient has not progressed, every attempt should be made to perform the subsequent assessments at their scheduled visits. This schedule is to be followed in order to minimise any unintentional bias caused by some patients being assessed at a different frequency than other patients.

1.4.2 Target lesions (TL)

Documentation of target lesions

A maximum of 5 measurable lesions, with a maximum of 2 lesions per organ (including lymph nodes), representative of all lesions involved should be identified as TL at baseline.

Target lesions should be selected on the basis of their size (longest diameter for non-nodal lesions or short axis for nodal lesions), but in addition should be those that lend themselves to reproducible repeated measurements. It may be the case that, on occasion, the largest lesion does not lend itself to reproducible measurement in which circumstance the next largest lesion, which can be measured reproducibly, should be selected.

The site and location of each TL should be documented as well as the longest diameter for non-nodal lesions (or short axis for lymph nodes). All measurements should be recorded in millimetres. At baseline the sum of the diameters for all TL will be calculated and reported as the baseline sum of diameters. At follow-up visits the sum of diameters for all TL will be calculated and reported as the follow-up sum of diameters.

Special cases:

- For TL measurable in 2 or 3 dimensions, always report the longest diameter. For pathological lymph nodes measurable in 2 or 3 dimensions, always report the short axis.
- If the CT/MRI slice thickness used is >5 mm, the minimum size of measurable disease at baseline should be twice the slice thickness of the baseline scan.

---

[1] Patients who cannot tolerate CT with contrast, despite pre-medications, may undergo a non-contrast CT scan of the chest and MRI of the abdomen (including pelvis).

- If a lesion has completely disappeared, the diameter should be recorded as 0 mm.
- If a TL splits into two or more parts, then record the sum of the diameters of those parts.
- If two or more TL merge then the sum of the diameters of the combined lesion should be recorded for one of the lesions and 0 mm recorded for the other lesion(s).
- If a TL is believed to be present and is faintly seen but too small to measure, a default value of 5mm should be assigned. If an accurate measure can be given, this should be recorded, even if it is below 5 mm.
- If a TL cannot be measured accurately due to it being too large, provide an estimate of the size of the lesion.
- When a TL has had any intervention e.g. radiotherapy, 71mbolization, surgery etc., during the study, the size of the TL should still be provided where possible.

Evaluation of target lesions

This section provides the definitions of the criteria used to determine objective tumour visit response for TL.

**Best Response**	**Criteria**
Complete Response (CR)	Disappearance of all target lesions since baseline determined by 2 observations not less than 4 weeks apart. Any pathological lymph nodes selected as target lesions must have a reduction in short axis to <10 mm.
Partial Response (PR)	At least a 30% decrease in the sum of diameters (SD) of TL determined by 2 observations not less than 4 weeks apart and taking as reference the baseline sum SD. (the scheduled tumour evaluation is 9 weeks apart) It is not necessary for all lesions to have regressed to qualify for partial response, but no lesions must have progressed and not one additional new lesion should appear.
Stable Disease (SD)	Neither sufficient shrinkage to qualify for PR nor sufficient increase to qualify for PD determined by 2 observations not less than 4 weeks apart.
Progression (PD)	At least a 20% increase in the SD of target lesions taking as reference the smallest SD recorded since the treatment started and minimum 5 mm increase over the nadir, or the appearance of one or more new lesions. Assignment to the progression category is done after 6 weeks from study entry. When the progression is observed before 6 weeks after entry in the study, the patient will be considered as an "early progression".
Not Evaluable (NE)	Only relevant if any of the target lesions were not assessed or not evaluable or had a lesion intervention at this visit. Note: If the sum of diameters meets the progressive disease criteria, progressive disease overrides not evaluable as a target lesion response

Table 2: Evaluation of Target-Measurable Lesions

1.4.3 Non-Target lesions (NTL)

Evaluation of non-target lesions

All other lesions (or sites of disease) not recorded as TL should be identified as NTL at baseline. Measurements are not required for these lesions, but their status should be followed at subsequent visits. At

each visit an overall assessment of the NTL response should be recorded by the Investigator. This section provides the definitions of the criteria used to determine and record overall response for NTL at the investigational site at each visit.

Best Response	Criteria
Complete Response (CR)	Disappearance of all NTLs determined by 2 observations not less than 4 weeks apart. All lymph nodes must be non-pathological in size (< 10 mm short axis).
Non-CR/Non-PD	Persistence of one or more NTLs
Progression (PD)	Appearance of one or more new lesions or unequivocal progression of existing NTLs.<br>Unequivocal progression of existing NTLs is defined as<br>• Overall level of substantial worsening in non-target disease such that, even in presence of SD or PR in target disease, the overall tumor burden has increased sufficiently to merit discontinuation of therapy<br>• In the absence of measurable disease, change in non-measurable disease comparable in magnitude to the increase that would be required to declare PD for measurable disease; examples include an increase in a pleural effusion from 'trace' to 'large', an increase in lymphangitic disease from localized to widespread<br>In all cases the progression MUST be clinically significant for the physician to consider changing (or stopping) therapy.<br>NB: In the case of radiological evidence of progression in bone at cycle 2, the tumour flare phenomenon should be considered and excluded prior to assigning progression "PD" to the patient's disease.
Not Evaluable (NE)	Only relevant when one or some of the NTLs were not assessed and, in the Investigator's opinion, they are not able to provide an evaluable overall non-target lesion assessment at this visit.<br>Note: For patients without target lesions at baseline, this is relevant if any of the non-target lesions were not assessed at this visit and the progression criteria have not been met

Table 3: Evaluation of Non Target Lesions

To achieve 'unequivocal progression' on the basis of non-target lesions, there must be an overall level of substantial worsening in non-target disease such that, even in presence of SD or PR in target lesions, the overall tumour burden has increased sufficiently to merit discontinuation of therapy. A modest 'increase' in the size of one or more non-target lesions is usually not sufficient to qualify for unequivocal progression status

1.4.4 New lesions

Details of any new lesions will also be recorded with the date of assessment. The presence of one or more new lesions is assessed as progression.

A lesion identified at a follow up assessment in an anatomical location that was not scanned at baseline is considered a new lesion and will indicate disease progression. The finding of a new lesion should be unequivocal: i.e. not attributable to differences in scanning technique, change in imaging modality or findings thought to represent something other than tumour.

If a new lesion is equivocal, for example because of its small size, the treatment and tumour assessments should be continued until the new lesion has been confirmed. If repeat scans confirm there is a new lesion, then the progression date should be declared using the date of the initial scan.

1.4.5 Symptomatic deterioration

Symptomatic deterioration is not a descriptor of an objective response: it is a reason for stopping study therapy. Patients with ‘symptomatic deterioration’ requiring discontinuation of treatment without objective evidence of disease progression at that time should continue to undergo tumour assessments where possible until objective disease progression is observed.

1.4.6 Evaluation of Overall Visit Response

The overall visit response will be derived using the algorithm shown in Table 14

Target Lesions	Non Target Lesions	New Lesions	Overall Response
CR	CR	No	CR
CR	NA	No	CR
NA	CR	No	CR
CR	Incomplete Response / SD	No	PR
CR	NE	No	PR
PR	Non-PD	No	PR
SD	Non-PD or NE	No	SD
NA	Incomplete Response / SD	No	SD
NE	Non-PD or NE	No	NE
NA	NE	No	NE
PD	Any	Yes or No	PD
Any	PD	Yes or No	PD
Any	Any	Yes	PD

Table 4: Determination of the overall response

CR = complete response, PR = partial response, SD = stable disease, PD = progressive disease, NE = not evaluable, NA = not applicable (only relevant if there were no TL/NTLs at baseline).

1.5 Specifications for radiological imaging

These notes are recommendations for use in clinical studies. The use of standardized protocols for CT and MRI allows comparability both within and between different studies, irrespective of where the examination has been undertaken.

1.5.1 CT Scan

CT scans of the chest abdomen and pelvis should be contiguous throughout all the anatomic region of interest. The most critical CT image acquisition parameters for optimal tumour evaluation using RECIST 1.1 are anatomic coverage, contrast administration, slice thickness, and reconstruction interval.

a. Anatomic coverage: Optimal anatomic coverage for most solid tumours is chest and abdomen (including pelvis). Coverage should encompass all areas of known predilection for metastases in the disease under evaluation and should additionally investigate areas that may be involved based on signs and symptoms of individual patients. Because a lesion later identified in a body part not scanned at baseline would be considered as a new lesion representing disease progression, careful consideration should be given to the extent of imaging coverage at baseline and at subsequent follow-up time points. This will enable better consistency not only of tumour measurements but also identification of new disease.

b. IV contrast administration: Optimal visualisation and measurement of metastases in solid tumours requires consistent administration (dose and rate) of IV contrast as well as timing of scanning. Typically, most abdominal imaging is performed during the portal venous phase and (optimally) about the same time frame after injection on each examination. An adequate volume of a suitable contrast agent should be given so that the metastases are demonstrated to best effect and a consistent method is used on subsequent examinations for any given patient. It is very important that the same technique be used at baseline and on follow- up examinations for a given patient. For patients who develop contraindications to contrast after baseline contrast CT is done, the decision as to whether non-contrast CT or MRI (enhanced or non-enhanced) should be performed should also be based on the tumour type, anatomic location of the disease a should be optimised to allow for comparison to the prior studies if possible. Each case should be discussed with the radiologist to determine if substitution of these other approaches is possible and, if not, the patient should be considered not evaluable from that point forward. Care must be taken in measurement of target lesions on a different modality and interpretation of non-target disease or new lesions, since the same lesion may appear to have a different size using a new modality. Oral contrast is recommended to help visualise and differentiate structures in the abdomen.

If iodine contrast media is medically contraindicated at baseline or at any time during the course of the study then the recommended methods are: CT thoracic examination without contrast and abdominal MRI with contrast. If MRI cannot be performed then CT without i.v. contrast is an option for the thorax and abdomen examination. For brain lesions assessment, MRI is the preferred method.

c. Slice thickness and reconstruction interval: It is recommended that CT scans be performed at 5mm contiguous slice thickness and this guideline presumes a minimum 5 mm thickness in recommendations for measurable lesion definition. Exceptionally, particular institutions may perform medically acceptable scans at slice thicknesses greater than 5 mm. If this occurs, the minimum size of measurable lesions at baseline should be twice the slice thickness of the baseline scans.

All window settings should be included in the assessment, particularly in the thorax where lung and soft tissue windows should be considered. When measuring lesions, the TL should be measured on the same window setting for repeated examinations throughout the study. All images from each examination should be included in the assessment and not “selected” images of the apparent lesion.

1.5.2 MRI Scan

MRI has excellent contrast, spatial and temporal resolution; however, there are many image acquisition variables involved in MRI, which greatly impact image quality, lesion conspicuity and measurement. Furthermore, the availability of MRI is variable globally. The modality used at follow-up should be the same as was used at baseline and the lesions should be measured/assessed on the same pulse sequence.

Generally, axial imaging of the abdomen and pelvis with T1 and T2 weighted imaging along with gadolinium-enhanced imaging should be performed. The field of view, matrix, number of excitations, phase encode steps, use of fat suppression and fast sequences should be optimised for the specific body part being imaged as well as the scanner utilised. It is beyond the scope appendix to prescribe specific MRI pulse sequence parameters for all scanners, body parts and diseases. Ideally, the same type of scanner should be used and the image acquisition protocol should be followed as closely as possible to prior scans. Body scans should be performed with breath-hold scanning techniques if possible. For these reasons, CT is the imaging modality of choice.

16 REFERENCES

1. Rahib L, Smith BD, Aizenberg R et al. Projecting cancer incidence and deaths to 2030: the unexpected burden of thyroid, liver, and pancreas cancers in the United States. Cancer Res 2014; 74: 2913-2921.
2. Hidalgo M. Pancreatic cancer. N Engl J Med 2010; 362: 1605-1617.
3. Conroy T, Desseigne F, Ychou M et al. FOLFIRINOX versus gemcitabine for metastatic pancreatic cancer. N Engl J Med 2011; 364: 1817-1825.
4. Von Hoff DD, Ervin T, Arena FP et al. Increased survival in pancreatic cancer with nab-paclitaxel plus gemcitabine. N Engl J Med 2013; 369: 1691-1703.
5. Jacobetz MA, Chan DS, Neesse A et al. Hyaluronan impairs vascular function and drug delivery in a mouse model of pancreatic cancer. Gut 2013; 62: 112-120.
6. Provenzano PP, Cuevas C, Chang AE et al. Enzymatic targeting of the stroma ablates physical barriers to treatment of pancreatic ductal adenocarcinoma. Cancer Cell 2012; 21: 418-429.
7. Beatty GL, Chiorean EG, Fishman MP et al. CD40 agonists alter tumor stroma and show efficacy against pancreatic carcinoma in mice and humans. Science 2011; 331: 1612-1616.
8. Beatty GL, Torigian DA, Chiorean EG et al. A phase I study of an agonist CD40 monoclonal antibody (CP-870,893) in combination with gemcitabine in patients with advanced pancreatic ductal adenocarcinoma. Clin Cancer Res 2013; 19: 6286-6295.
9. Ene-Obong A, Clear AJ, Watt J et al. Activated pancreatic stellate cells sequester CD8+ T cells to reduce their infiltration of the juxtatumoral compartment of pancreatic ductal adenocarcinoma. Gastroenterology 2013; 145: 1121-1132.
10. Feig C, Jones JO, Kraman M et al. Targeting CXCL12 from FAP-expressing carcinoma-associated fibroblasts synergizes with anti-PD-L1 immunotherapy in pancreatic cancer. Proc Natl Acad Sci U S A 2013; 110: 20212-20217.
11. Pylayeva-Gupta Y, Lee KE, Hajdu CH et al. Oncogenic Kras-induced GM-CSF production promotes the development of pancreatic neoplasia. Cancer Cell 2012; 21: 836-847.
12. Olive KP, Jacobetz MA, Davidson CJ et al. Inhibition of Hedgehog Signaling Enhances Delivery of Chemotherapy in a Mouse Model of Pancreatic Cancer. Science 2009; 324: 1457-1461.
13. Neesse A, Frese KK, Bapiro TE et al. CTGF antagonism with mAb FG-3019 enhances chemotherapy response without increasing drug delivery in murine ductal pancreas cancer. Proc Natl Acad Sci U S A 2013; 110: 12325-12330.
14. Borad MJ, Reddy SG, Bahary N et al. Randomized Phase II Trial of Gemcitabine Plus TH-302 Versus Gemcitabine in Patients With Advanced Pancreatic Cancer. J Clin Oncol 2014.
15. Plentz R, Park JS, Rhim AD et al. Inhibition of gamma-secretase activity inhibits tumor progression in a mouse model of pancreatic ductal adenocarcinoma. Gastroenterology 2009; 136: 1741-1749 e1746.
16. Wong PP, Demircioglu F, Ghazaly E et al. Dual-Action Combination Therapy Enhances Angiogenesis while Reducing Tumor Growth and Spread. Cancer Cell 2015; 27: 123-137.
17. Ozdemir BC, Pentcheva-Hoang T, Carstens JL et al. Depletion of carcinoma-associated fibroblasts and fibrosis induces immunosuppression and accelerates pancreas cancer with reduced survival. Cancer Cell 2014; 25: 719-734.
18. Rhim AD, Oberstein PE, Thomas DH et al. Stromal elements act to restrain, rather than support, pancreatic ductal adenocarcinoma. Cancer Cell 2014; 25: 735-747.
19. Apte MV, Wilson JS, Lugea A, Pandol SJ. A starring role for stellate cells in the pancreatic cancer microenvironment. Gastroenterology 2013; 144: 1210-1219.
20. Sherman MH, Yu RT, Engle DD et al. Vitamin d receptor-mediated stromal reprogramming suppresses pancreatitis and enhances pancreatic cancer therapy. Cell 2014; 159: 80-93.
21. Froeling FE, Feig C, Chelala C et al. Retinoic acid-induced pancreatic stellate cell quiescence reduces paracrine Wnt-beta-catenin signaling to slow tumor progression. Gastroenterology 2011; 141: 1486-1497, 1497 e1481-1414.

22. Von Hoff DD, Ramanathan RK, Borad MJ et al. Gemcitabine plus nab-paclitaxel is an active regimen in patients with advanced pancreatic cancer: a phase I/II trial. J Clin Oncol 2011; 29: 4548-4554.

23. Alvarez R, Musteanu M, Garcia-Garcia E et al. Stromal disrupting effects of nab-paclitaxel in pancreatic cancer. Br J Cancer 2013; 109: 926-933.

24. Frese KK, Neesse A, Cook N et al. nab-Paclitaxel Potentiates Gemcitabine Activity by Reducing Cytidine Deaminase Levels in a Mouse Model of Pancreatic Cancer. Cancer Discov 2012; 2: 260-269.

25. Yardley DA. nab-Paclitaxel mechanisms of action and delivery. J Control Release 2013; 170: 365-372.

26. Mukherjee S, Hurt CN, Bridgewater J et al. Gemcitabine-based or capecitabine-based chemoradiotherapy for locally advanced pancreatic cancer (SCALOP): a multicentre, randomised, phase 2 trial. Lancet Oncol 2013; 14: 317-326.

27. Marchetti MN, Sampol E, Bun H et al. In vitro metabolism of three major isomers of retinoic acid in rats. Intersex and interstrain comparison. Drug Metab Dispos 1997; 25: 637-646.

28. Marill J, Idres N, Capron CC et al. Retinoic acid metabolism and mechanism of action: a review. Curr Drug Metab 2003; 4: 1-10.

29. Rhinn M, Dolle P. Retinoic acid signalling during development. Development 2012; 139: 843-858.

30. Ross AC, Zolfaghari R. Cytochrome P450s in the regulation of cellular retinoic acid metabolism. Annu Rev Nutr 2011; 31: 65-87.

31. Adamson PC. Clinical and pharmacokinetic studies of all-trans-retinoic acid in pediatric patients with cancer. Leukemia 1994; 8 Suppl 3: S22-25.

32. Adamson PC, Bailey J, Pluda J et al. Pharmacokinetics of all-trans-retinoic acid administered on an intermittent schedule. J Clin Oncol 1995; 13: 1238-1241.

33. Adamson PC, Reaman G, Finklestein JZ et al. Phase I trial and pharmacokinetic study of all-trans-retinoic acid administered on an intermittent schedule in combination with interferon-alpha2a in pediatric patients with refractory cancer. J Clin Oncol 1997; 15: 3330-3337.

34. Conley BA, Egorin MJ, Sridhara R et al. Phase I clinical trial of all-trans-retinoic acid with correlation of its pharmacokinetics and pharmacodynamics. Cancer Chemother Pharmacol 1997; 39: 291-299.

35. Sutton LM, Warmuth MA, Petros WP, Winer EP. Pharmacokinetics and clinical impact of all-trans retinoic acid in metastatic breast cancer: a phase II trial. Cancer Chemother Pharmacol 1997; 40: 335-341.

36. Trump DL, Smith DC, Stiff D et al. A phase II trial of all-trans-retinoic acid in hormone-refractory prostate cancer: a clinical trial with detailed pharmacokinetic analysis. Cancer Chemother Pharmacol 1997; 39: 349-356.

37. Adedoyin A, Stiff DD, Smith DC et al. All-trans-retinoic acid modulation of drug-metabolizing enzyme activities: investigation with selective metabolic drug probes. Cancer Chemother Pharmacol 1998; 41: 133-139.

38. Budd GT, Adamson PC, Gupta M et al. Phase I/II trial of all-trans retinoic acid and tamoxifen in patients with advanced breast cancer. Clin Cancer Res 1998; 4: 635-642.

39. Escudier B, Ravaud A, Berton D et al. Phase II study of interferon-alpha and all-trans retinoic acid in metastatic renal cell carcinoma. J Immunother 1998; 21: 62-64.

40. Weiss GR, Liu PY, Alberts DS et al. 13-cis-retinoic acid or all-trans-retinoic acid plus interferon-alpha in recurrent cervical cancer: a Southwest Oncology Group phase II randomized trial. Gynecol Oncol 1998; 71: 386-390.

41. Estey EH, Thall PF, Pierce S et al. Randomized phase II study of fludarabine + cytosine arabinoside + idarubicin +/- all-trans retinoic acid +/- granulocyte colony-stimulating factor in poor prognosis newly diagnosed acute myeloid leukemia and myelodysplastic syndrome. Blood 1999; 93: 2478-2484.

42. Park SH, Gray WC, Hernandez I et al. Phase I trial of all-trans retinoic acid in patients with treated head and neck squamous carcinoma. Clin Cancer Res 2000; 6: 847-854.

43. Seiter K, Feldman EJ, Dorota Halicka H et al. Clinical and laboratory evaluation of all-trans retinoic acid modulation of chemotherapy in patients with acute myelogenous leukaemia. Br J Haematol 2000; 108: 40-47.

44. Dutcher JP, Lee S, Gallagher RE et al. Phase II study of all-trans retinoic acid in the accelerated phase or early blastic phase of chronic myeloid leukemia: a study of the Eastern Cooperative Oncology Group (E1993). Leuk Lymphoma 2005; 46: 377-385.

45. Arrieta O, Gonzalez-De la Rosa CH, Arechaga-Ocampo E et al. Randomized phase II trial of All-trans-retinoic acid with chemotherapy based on paclitaxel and cisplatin as first-line treatment in patients with advanced non-small-cell lung cancer. J Clin Oncol 2010; 28: 3463-3471.

46. Bryan M, Pulte ED, Toomey KC et al. A pilot phase II trial of all-trans retinoic acid (Vesanoid) and paclitaxel (Taxol) in patients with recurrent or metastatic breast cancer. Invest New Drugs 2011; 29: 1482-1487.

47. Burnett AK, Grimwade D, Solomon E et al. Presenting white blood cell count and kinetics of molecular remission predict prognosis in acute promyelocytic leukemia treated with all-trans retinoic acid: result of the Randomized MRC Trial. Blood 1999; 93: 4131-4143.

48. Burnett AK, Hills RK, Grimwade D et al. Inclusion of chemotherapy in addition to anthracycline in the treatment of acute promyelocytic leukaemia does not improve outcomes: results of the MRC AML15 trial. Leukemia 2013; 27: 843-851.

49. Brembeck FH, Schoppmeyer K, Leupold U et al. A phase II pilot trial of 13-cis retinoic acid and interferon-alpha in patients with advanced pancreatic carcinoma. Cancer 1998; 83: 2317-2323.

50. Moore DF, Jr., Pazdur R, Sugarman S et al. Pilot phase II trial of 13-cis-retinoic acid and interferon-alpha combination therapy for advanced pancreatic adenocarcinoma. Am J Clin Oncol 1995; 18: 525-527.

51. Michael A, Hill M, Maraveyas A et al. 13-cis-Retinoic acid in combination with gemcitabine in the treatment of locally advanced and metastatic pancreatic cancer--report of a pilot phase II study. Clin Oncol (R Coll Radiol) 2007; 19: 150-153.

52. Pili R, Salumbides B, Zhao M et al. Phase I study of the histone deacetylase inhibitor entinostat in combination with 13-cis retinoic acid in patients with solid tumours. Br J Cancer 2012; 106: 77-84.

53. Recchia F, Sica G, Candeloro G et al. Chemoradioimmunotherapy in locally advanced pancreatic and biliary tree adenocarcinoma: a multicenter phase II study. Pancreas 2009; 38: e163-168.

54. Segawa K, Nakazawa S, Tsukamoto Y et al. Determination of plasma retinol by high performance liquid chromatography, and its significance in digestive diseases. Jpn J Med 1986; 25: 20-24.

55. Rocchi E, Casalgrandi G, Ronzoni A et al. Retinol and tocopherol content in primary and metastatic digestive neoplasms. Anticancer Res 2003; 23: 5049-5054.

56. Stafford D, Prince VE. Retinoic acid signaling is required for a critical early step in zebrafish pancreatic development. Curr Biol 2002; 12: 1215-1220.

57. Kobayashi H, Spilde TL, Bhatia AM et al. Retinoid signaling controls mouse pancreatic exocrine lineage selection through epithelial-mesenchymal interactions. Gastroenterology 2002; 123: 1331-1340.

58. Middleton G, Silcocks P, Cox T et al. Gemcitabine and capecitabine with or without telomerase peptide vaccine GV1001 in patients with locally advanced or metastatic pancreatic cancer (TeloVac): an open-label, randomised, phase 3 trial. Lancet Oncol 2014.

59. Watt J, Kocher HM. The desmoplastic stroma of pancreatic cancer is a barrier to immune cell infiltration. Oncoimmunology 2013; 2: e26788.

60. Coleman SJ, Chioni AM, Ghallab M et al. Nuclear translocation of FGFR1 and FGF2 in pancreatic stellate cells facilitates pancreatic cancer cell invasion. EMBO Mol Med 2014; 6: 467-481.

61. Kadaba R, Birke H, Wang J et al. Imbalance of desmoplastic stromal cell numbers drives aggressive cancer processes. J Pathol 2013; 230: 107-117.

62. Micallef SJ, Janes ME, Knezevic K et al. Retinoic acid induces Pdx1-positive endoderm in differentiating mouse embryonic stem cells. Diabetes 2005; 54: 301-305.

63. Philip PA, Mooney M, Jaffe D et al. Consensus report of the national cancer institute clinical trials planning meeting on pancreas cancer treatment. J Clin Oncol 2009; 27: 5660-5669.

64. Van Laethem JL, Verslype C, Iovanna JL et al. New strategies and designs in pancreatic cancer research: consensus guidelines report from a European expert panel. Ann Oncol 2012; 23: 570-576.

65. Von Hoff DD. Actionable targets in pancreatic cancer detected by immunohistochemistry

(IHC), microarray (MA) fluorescent in situ hybridization (FISH), and
mutational analysis. (Abstract #4013). ASCO Proceedings 2012.

66. The use of gemcitabine for the treatment of pancreatic cancer (TA25). In. National Institute for Health and Clinical Excellence 2001.

67. Di Febo A, Laurenti L, Falcucci P et al. All-trans retinoic acid in association with low dose cytosine arabinoside in the treatment of acute myeoid leukemia in elderly patients. Am J Ther 2007; 14: 351-355.

68. Elisabete Carapuça EG, Christine Feig, David A. Tuveson, Hemant Kocher. Developing Drug Combinations to Co-Target Pancreatic Cancer and Its Supporting Stroma. Gastroenterology 2014; 146: S498. (abstract)

69. Gupta S, Pramanik D, Mukherjee R et al. Molecular determinants of retinoic acid sensitivity in pancreatic cancer. Clin Cancer Res 2012; 18: 280-289.

70. Tan DS, Thomas GV, Garrett MD et al. Biomarker-driven early clinical trials in oncology: a paradigm shift in drug development. Cancer J 2009; 15: 406-420.

71. Yap TA, Sandhu SK, Workman P, de Bono JS. Envisioning the future of early anticancer drug development. Nat Rev Cancer 2010; 10: 514-523.

72. di Masi A, Leboffe L, De Marinis E et al. Retinoic acid receptors: From molecular mechanisms to cancer therapy. Mol Aspects Med 2015; 41C: 1-115.

73. Everts HB. Endogenous retinoids in the hair follicle and sebaceous gland. Biochim Biophys Acta 2012; 1821: 222-229.

74. Lotan R, Xu XC, Lippman SM et al. Suppression of retinoic acid receptor-beta in premalignant oral lesions and its up-regulation by isotretinoin. N Engl J Med 1995; 332: 1405-1410.

75. Iasonos A, O'Quigley J. Adaptive dose-finding studies: a review of model-guided phase I clinical trials. J Clin Oncol 2014; 32: 2505-2511.

76. Andrei G, van den Oord J, Fiten P et al. Organotypic epithelial raft cultures as a model for evaluating compounds against alphaherpesviruses. Antimicrob Agents Chemother 2005; 49: 4671-4680.

77. Jennifer Watt IS, Tomas Dowe, Tatjana Crnogorac-Jurcevic, Paola Allavena, Hemant Kocher. Role of PTX3 in pancreatic cancer. The Lancet 2014. (abstract)

78. Loprevite M, Tiseo M, Chiaramondia M et al. Buccal mucosa cells as in vivo model to evaluate gefitinib activity in patients with advanced non small cell lung cancer. Clin Cancer Res 2007; 13: 6518-6526.
