# Supplementary material for: Phase I clinical trial repurposing all-trans retinoic acid as a stromal targeting agent for pancreatic cancer
Source: Nat Commun. 2020 Sep 24;11:4841. doi: 10.1038/s41467-020-18636-w (PMC7518421; doi:10.1038/s41467-020-18636-w)
Supplement: Supplementary file 3 — Reporting Summary [file 41467_2020_18636_MOESM3_ESM.pdf]

nature research

Corresponding author(s): Hemant Kocher

Last updated by author(s): Feb 26, 2020

Reporting Summary

Nature Research wishes to improve the reproducibility of the work that we publish. This form provides structure for consistency and transparency in reporting. For further information on Nature Research policies, see Authors & Referees and the Editorial Policy Checklist .

Statistics

For all statistical analyses, confirm that the following items are present in the figure legend, table legend, main text, or Methods section.

n/a	Confirmed	
☐	☒	The exact sample size (*n*) for each experimental group/condition, given as a discrete number and unit of measurement
☐	☒	A statement on whether measurements were taken from distinct samples or whether the same sample was measured repeatedly
☐	☒	The statistical test(s) used AND whether they are one- or two-sided<br>*Only common tests should be described solely by name; describe more complex techniques in the Methods section.*
☐	☒	A description of all covariates tested
☐	☒	A description of any assumptions or corrections, such as tests of normality and adjustment for multiple comparisons
☐	☒	A full description of the statistical parameters including central tendency (e.g. means) or other basic estimates (e.g. regression coefficient) AND variation (e.g. standard deviation) or associated estimates of uncertainty (e.g. confidence intervals)
☐	☒	For null hypothesis testing, the test statistic (e.g. *F*, *t*, *r*) with confidence intervals, effect sizes, degrees of freedom and *P* value noted<br>*Give P values as exact values whenever suitable.*
☐	☒	For Bayesian analysis, information on the choice of priors and Markov chain Monte Carlo settings
☒	☐	For hierarchical and complex designs, identification of the appropriate level for tests and full reporting of outcomes
☒	☐	Estimates of effect sizes (e.g. Cohen's *d*, Pearson's *r*), indicating how they were calculated

Our web collection on statistics for biologists contains articles on many of the points above.

Software and code

Policy information about availability of computer code

Data collection	All clinical data were collected on an in-house built Electronic Case Report Form (eCRF) designed using ORACLE v11.2.0. Sample size calculations were performed using the software package PASS version 12.0.<br>Diffusion-weighted images were analyzed by a board-certified radiologist in OsiriX version 9.0<br>In the laboratory images were visualized using Zeiss Zen 2.3 software
Data analysis	All clinical efficacy endpoints were analyzed using STATA version 13.1. Laboratory data were analyzed using PRISM (GraphPad Inc) version 8. Statistical tests are described as used.

For manuscripts utilizing custom algorithms or software that are central to the research but not yet described in published literature, software must be made available to editors/reviewers. We strongly encourage code deposition in a community repository (e.g. GitHub). See the Nature Research guidelines for submitting code & software for further information.

Data

Policy information about availability of data

All manuscripts must include a data availability statement. This statement should provide the following information, where applicable:

- Accession codes, unique identifiers, or web links for publicly available datasets
- A list of figures that have associated raw data
- A description of any restrictions on data availability

'The data supporting this Article are available within the Article, Supplementary Information or available from the authors upon request.

Field-specific reporting

Please select the one below that is the best fit for your research. If you are not sure, read the appropriate sections before making your selection.

☒ Life sciences ☐ Behavioural & social sciences ☐ Ecological, evolutionary & environmental sciences

For a reference copy of the document with all sections, see nature.com/documents/nr-reporting-summary-flat.pdf

Life sciences study design

All studies must disclose on these points even when the disclosure is negative.

Sample size	This is described in BMC Cancer. 2019 Jun 26;19(1):632. doi: 10.1186/s12885-019-5801-3.
Data exclusions	No data were excluded. Where data is not available (reduced n), the correct 'n' is mentioned in each figure.
Replication	All coefficient of variation are produced for DW-MRI, pharmacokinetic and biomarker assays. All data were reproducible with low coefficient of variation. These are reported in respective sections. Briefly two board certified radiologist assessed DW-MRI images. Two laboratory scientists assessed immunoflourescene images with inter-day reproducibility using standards. PTX3 assays were replicated in duplicate with overalpping standard curves across assays conducted on different days.
Randomization	Not applicable, as it was a phase I clinical trial. In this dose finding and expansion study, randomisation was not required but allocation to a dose level was done by a two-step adaptive Bayesian continual reassessment method .
Blinding	Not applicable for patients, as it was an open label trial. However, for pharmacokinetic, pharmacodynamic and biomarker analysis the respective laboratories were blinded to patient dose level or outcome.

Reporting for specific materials, systems and methods

We require information from authors about some types of materials, experimental systems and methods used in many studies. Here, indicate whether each material, system or method listed is relevant to your study. If you are not sure if a list item applies to your research, read the appropriate section before selecting a response.

Materials & experimental systems

n/a	Involved in the study	
☐	☒	Antibodies
☒	☐	Eukaryotic cell lines
☒	☐	Palaeontology
☒	☐	Animals and other organisms
☐	☒	Human research participants
☐	☒	Clinical data

Methods

n/a	Involved in the study	
☒	☐	ChIP-seq
☒	☐	Flow cytometry
☒	☐	MRI-based neuroimaging

Antibodies

Antibodies used	Listed in extended data table 11.<br><br>For immunoflourescence: Rat FABP5 (R&D Systems, MAB3077), Mouse CRABP2 (Santacruz biotechnology, sc-159411), rabbit cytokeratin (DAKO, Z0622), Mouse a-SMA (Sigma Aldrich F3777), Rabbit IgG-488 (Thermofisher A11034), Rat IgG (Thermofisher A11081), Mouse IgG-546 (Thermofisher A11030).<br><br>For ELISA: monclonal antibody MNB4 (Enzo Life Sciences ALX-804-464-C100). biotinylated PTX3 (Enzo life Sciences, cat. ALX-210-365B)
Validation	Described in methods and datasheet on websites of each of the products.<br><br>For staining of tissue sections: Organotypic sections, as previously described (Carapuca E et al, J Pathol 2016), were used for positive and negative staining controls. Controls were uniformly negative with appropriate isotype-specific immunoglobulin at matching dilutions. Antibody and staining scoring validation is described in Hughes et al Ann Diagno Pathol 2020.<br><br>For ELISA: Validated protocol is described in Latini, Circulation 2004.

Human research participants

Policy information about studies involving human research participants

Population characteristics	Described in extended data, table 1.<br>Briefly median age was 66 (range 27-78) years of 27 patients, of which 11 were women, and 20 Caucasians. 4 had locally advanced pancreatic cancer and 23 had metastatic pancreatic cancer. 16 had performance status 0 and 11 had performance status of 1.
Recruitment	Described in methods.<br>STARPAC was an investigator-initiated open-label, multicentre, phase Ib study of ATRA administered with gemcitabine and nab-paclitaxel in patients with locally advanced or metastatic pancreatic cancer, who had not received prior systemic therapy for their disease. Additional eligibility criteria included World Health Organisation (WHO) performance status 0 or 1, life expectancy ≥12 weeks and adequate haematologic and end-organ function within 14 days prior to the first study treatment. Major exclusion criteria were known brain metastases, pre-existing sensory neuropathy (>grade 1) and serious medical risk factors involving any major organ systems, or serious psychiatric disorders, which could compromise the patient's safety or the study data integrity.<br>Study was sponsored by Barts Health NHS Trust.<br><br>Patients were invited to participate in this clinical trial from outpatient clinics at the first visit for receiving palliative chemotherapy for advanced pancreatic cancer. Self-selection bias into clinical trial may occur with the most interested and keen willing to participate rather than those uninterested, and there is no way to eliminate this bias as with all clinical trials. However this potential self-election bias is unlikely to impact results. There were no other biases as the inclusion and exclusion criteria were robust.
Ethics oversight	All patients provided written informed consent. Ethical approval for STARPAC clinical trial; South Central - Berkshire Research Ethics Committee; 15/SC/0548 dated 13 October 2015.<br><br>The study was sponsored by Barts Health NHS Trust. The Centre for Experimental Cancer Medicine (CECM), Barts Cancer Institute, Queen Mary University of London had overall responsibility for trial management. The Trial Management Group (TMG) was responsible for day-to-day running of the trial. Safety data was reviewed regularly by the Safety Review Committee (SRC).<br><br>All samples had a valid chain of custody throughout procurement, temporary storage at site, shipping, and permanent storage at the Barts Pancreas Tissue Bank (BPTB, REC Ref: 13/SC/0592, HTA License number: 12199) and were given to laboratory staff via a traceable database.

Note that full information on the approval of the study protocol must also be provided in the manuscript.

Clinical data

Policy information about clinical studies

All manuscripts should comply with the ICMJEguidelines for publication of clinical research and a completedCONSORT checklist must be included with all submissions.

Clinical trial registration	https://clinicaltrials.gov/ct2/show/NCT03307148
Study protocol	Full Protocol can be accessed in Supplementary Material.
Data collection	Patients were recruited from four sites (Barts Health NHS Trust, Cambridge University Hospitals Foundation NHS Trust, Imperial College Healthcare NHS Trust, Guy's and St Thomas' NHS Foundation Trust) to the study between February 2016 and February 2018, with a final data collection cut off on the 1 April 2019. eCRF database is located at CECM, Barts Cancer Institute, Queen Mary University of London, UK
Outcomes	Details are accessible from the full Protocol.<br>It was expected that a maximum of 24 evaluable patients would be enrolled into Part 1 of the study based on CRM. For Part 1, the primary objective was to determine the MTD of the combination of gemcitabine-nab-paclitaxel and ATRA, measured by the occurrence of DLTs during the first 28 days of treatment that were attributed as possibly, probably or definitely related to the study treatment. For Part 2, a sample size of 10 was considered reasonable to provide indicative data on OBD.<br><br>Secondary endpoints included analyses of PK parameters, response rates, progression-free survival (PFS), overall survival (OS), and safety. For all time-to-event analyses performed, patients who did not have an event were right censored:- PFS censored on the last date the patient was known to be progression-free; OS censored at the date of last contact within 12 months of enrolment into trial. Post-hoc OS analysis was carried out for data beyond 12 months as there were exceptional survivors. Survival endpoints were shown graphically with Kaplan-Meier plots.<br><br>All efficacy analyses were performed on the evaluable population which included all patients receiving at least two cycles of the combination or progressing within the first two cycles, regardless of whether they were later found to be ineligible or a protocol violator. Safety analyses included all patients who received at least one dose of study treatment. The worst grade of each adverse event (AE) for each patient during study treatment was reported. Cumulative dose intensity over the first 6 cycles was calculated as the actual amount of study drug received over the first 6 cycles divided by the expected amount of study drug received over the first 6 cycles. The expected amount of study drug was calculated based on the dose and schedule specified in the study protocol.
