# Supplementary material for: Phase I clinical trial repurposing all-trans retinoic acid as a stromal targeting agent for pancreatic cancer
Source: Nat Commun. 2020 Sep 24;11:4841. doi: 10.1038/s41467-020-18636-w (PMC7518421; doi:10.1038/s41467-020-18636-w)
Supplement: Supplementary file 2 — Peer Review File [file 41467_2020_18636_MOESM2_ESM.pdf]

Editorial Note: This manuscript has been previously reviewed at another journal that is not operating a transparent peer review scheme. The manuscript was considered suitable for publication without further review at *Nature Communications*.
